# A Prospective, Open-Label Pilot Study of Concurrent Male Partner Treatment for Bacterial Vaginosis

**DOI:** 10.1128/mBio.02323-21

**Published:** 2021-10-19

**Authors:** Erica L. Plummer, Lenka A. Vodstrcil, Michelle Doyle, Jennifer A. Danielewski, Gerald L. Murray, Glenda Fehler, Christopher K. Fairley, Dieter M. Bulach, Suzanne M. Garland, Eric P. F. Chow, Jane S. Hocking, Catriona S. Bradshaw

**Affiliations:** a Central Clinical School, Monash University, Melbourne, Australia; b Melbourne Sexual Health Centre, Alfred Health, Melbourne, Australia; c Melbourne School of Population and Global Health, The University of Melbourne, Melbourne, Australia; d Molecular Microbiology Research Group, Murdoch Children’s Research Institute, Melbourne, Australia; e Centre for Women’s Infectious Diseases, The Royal Women’s Hospital, Melbourne, Australia; f Department of Obstetrics and Gynaecology, The University of Melbourne, Melbourne, Australia; g Microbiological Diagnostic Unit Public Health Laboratory, The Peter Doherty Institute for Infection and Immunity, The University of Melbourne, Melbourne, Australia; h Melbourne Bioinformatics, The University of Melbourne, Melbourne, Australia; Brigham and Women's Hospital

**Keywords:** bacterial vaginosis, genital microbiota, partner treatment

## Abstract

Up to 50% of women receiving first-line antibiotics for bacterial vaginosis (BV) experience recurrence within 12 weeks. Evidence suggests that reinfection from an untreated regular sexual partner contributes to recurrence. We conducted a pilot study of 34 heterosexual couples to describe the impact of concurrent partner treatment on the composition of the genital microbiota over a 12-week period. We also determined the acceptability and tolerability of concurrent partner treatment and obtained preliminary estimates of the efficacy of the intervention to inform a randomized controlled trial (RCT). Women received first-line antibiotic treatment for BV (i.e., oral metronidazole or intravaginal clindamycin), and their male partner received oral metronidazole, 400 mg, and 2% clindamycin cream applied topically to penile skin, both twice daily for 7 days. The genital microbiota was characterized at three anatomical sites (women, vaginal; men, cutaneous penile and first-pass urine [representing the urethra]) using 16S rRNA gene sequencing. Immediately posttreatment, concurrent partner treatment significantly reduced the abundance of BV-associated bacteria (false-discovery rate [FDR] corrected *P* value < 0.05) and altered the overall microbiota composition of all three anatomical sites (*P* = 0.001). Suppression of BV-associated bacteria was sustained in the majority (81%) of women over the 12-week period (FDR *P* value < 0.05), despite BV-associated bacteria reemerging at both genital sites in men. In this cohort of women at high risk for recurrence, five recurred within 12 weeks of treatment (17%; 95% confidence interval [CI], 6 to 34%). Importantly, men tolerated and adhered to combination therapy. Our findings provide support for an RCT of combined oral and topical male partner treatment for BV.

## INTRODUCTION

Bacterial vaginosis (BV) is a highly prevalent vaginal condition that is associated with obstetric and gynecological sequelae and has significant implications for health care expenditure (1). The vaginal microbiota in women with BV is characterized by a reduction in *Lactobacillus* spp. and an increase in obligate and facultative anaerobes collectively termed BV-associated bacteria (including *Gardnerella* spp., Atopobium vaginae, *Prevotella* spp., and *Sneathia* spp. among others) (2). Recurrence is frustratingly common, and treatments that achieve long-term cure are lacking. Up to 50% of women experience recurrence within the 12 weeks following first-line antibiotic treatment (3, 4), and epidemiological studies have shown that women who have the same regular sexual partner (RSP) pre- and posttreatment for BV are at 2- to 3-fold increased risk of recurrence (3, 5, 6). Despite a growing body of evidence that sexual transmission plays an important role in the pathogenesis of BV acquisition and recurrence, current treatments for BV only target the affected woman (7).

Our group is conducting a program of research investigating the acceptability, tolerability, and efficacy of concurrent partner treatment for improving BV cure. We previously published an exploratory study of 22 heterosexual couples receiving concurrent antimicrobial therapy for BV (8). Women received first-line BV-treatment (oral metronidazole or intravaginal clindamycin), and males received combined topical and oral antimicrobial therapy (oral metronidazole and topical clindamycin applied to the penile skin). As BV-associated bacteria have been detected on the coronal sulcus/glans penis and in the distal urethra, as well as in urine and semen samples (9–12), both oral and topical antibiotics were used in males to address multisite carriage. In this exploratory study, we followed couples for 3 weeks posttreatment and found that suppression of BV-associated bacteria was sustained in the majority of women. These data are encouraging, as women in this cohort were at high risk of recurrence, all were having unprotected sex with their regular male partner, who were predominantly uncircumcised, and the majority had a history of BV (8). Although this earlier exploratory study provided support for continued investigation into concurrent partner treatment for BV, it was limited by a short duration of follow-up and suboptimal sampling of the urethral site in males. This affected our ability to assess the durability and long-term impact of partner treatment on the genital microbiota, and particularly at the male urethral site. This is relevant because a recent study found that both the urethral and cutaneous penile microbiota were accurate predictors of incident BV in female sexual partners, with the composition of the urethral site having slightly higher prediction accuracy than the cutaneous site (12).

The objectives of the present study were to (i) assess the impact of concurrent partner treatment on three anatomical sites (the vagina, cutaneous penile site, and male urethra) over a 12-week period, (ii) determine the adherence to, and acceptability and tolerability of, concurrent male partner treatment, and (iii) provide preliminary estimates of the efficacy of the intervention in order to inform a randomized controlled trial (RCT).

## RESULTS

### Participant flow and recruitment.

**FIG 1:**
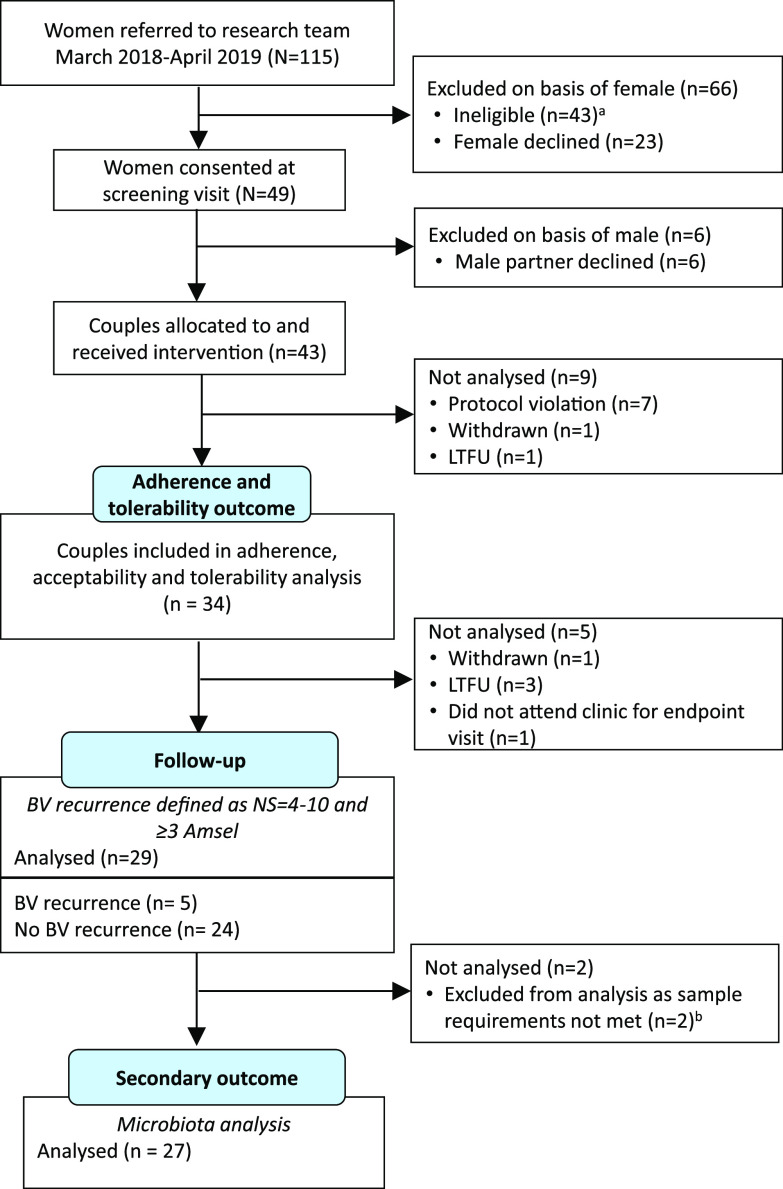
CONSORT diagram of participant flow through the study. LTFU, loss to follow up. *^a^*Reasons for ineligibility were no BV by the study criteria (*n* = 12), the couple was unable to comply with study procedures (*n* = 11), the woman was unable to stay and discuss the study (*n* = 7), one or both partners reported other sexual partners (*n* = 6), the relationship was <2 months in duration (*n* = 4), the woman was diagnosed with current pelvic inflammatory disease (PID) (*n* = 3). *^b^*Endpoint specimens for microbiota analysis were missing from one female (who experienced BV recurrence) and from one male (whose female partner was cured).

From March 2018 to March 2019, 115 women attending the Melbourne Sexual Health Centre (MSHC) who were diagnosed with BV were referred to the research team and screened for eligibility (Fig. 1); 43 women were ineligible and 23 declined. Of note, 11 women were excluded because they felt their partner would not be interested in participating or they did not want to discuss the study with their partner. Of the 49 women who consented, 43 male partners (88%) agreed to participate. Of the 43 couples who received the intervention, 7 were protocol violations (male did not return any study packs) and two completed baseline procedures only. A total of 34 couples (79%) provided both baseline (day 0) and day 8 data and contributed to adherence and tolerability analyses.

### Baseline data.

Baseline demographic and behavioral characteristics for the 34 couples are provided in Table 1. Most women reported a history of BV (*n* = 27, 79%), 18 (53%) reported current hormonal contraceptive use, and 12 (35%) had an intrauterine device (IUD) *in situ* (6 reported using a levonorgestrel IUD and 6 reported using a copper IUD). Most men were uncircumcised (*n* = 29, 85%). The median duration of their relationship was 18 months (interquartile range [IQR], 8 to 36 months), and all couples reported condomless penile-vaginal sex in the month prior to enrollment.

**TABLE 1:** Demographic and behavioral characteristics of couples at baseline^*a*^

Characteristic	Data for:	
	Female (*n* = 34)	Male (*n* = 34)
Age in yrs [median (IQR)]	30 (27–34)	31 (27–37)
Country of Birth		
Australia	18 (53)	23 (70)
Other	16 (47)^*b*^	10 (30)^*c*^
Current smoker		
No	26 (79)	20 (61)
Yes	7 (21)	13 (39)
History of BV		
No	7 (21)	
Yes	27 (79)	
Mos since last BV [median (IQR)]	3 (2–12)	
Current method of contraception		
None	6 (18)	
Condoms only	4 (12)	
Copper intrauterine device	6 (18)	
Oral contraceptive pill	8 (24)	
Hormonal intrauterine device	6 (18)	
Other hormonal method of contraception^*d*^	4 (12)	
Current douching^*e*^		
No	31 (91)	
Yes	3 (9)	
Circumcised		
No		29 (85)
Yes		5 (15)
No. of sexual partners in last 3 mos^*f*^		
1	25 (74)	22 (71)
≥2	9 (26)	9 (29)
No. of lifetime sexual partners		
1–7	11 (34)	6 (19)
8–20	10 (31)	10 (32)
≥21	11 (34)	15 (48)
Duration of current partnership in mos [median (IQR)]^*g*^	18 (8–36)	21 (9–36)
Any condomless vaginal sex in last mo		
No	0 (0)	0 (0)
Yes	34 (100)	32 (100)
Any condomless anal sex in last mo		
No	25 (74)	24 (75)
Yes	9 (26)	8 (25)
Any oral sex received in the last mo		
No	7 (21)	3 (10)
Yes	27 (79)	28 (90)
Antibiotics taken in last mo		
No	28 (82)	29 (88)
Yes	6 (18)^*h*^	4 (12)^*i*^
Vaginal treatments used in last mo		
No	32 (94)	
Yes	2 (6)^*j*^	
Treatments on penis used in last mo		
No		30 (94)
Yes		2 (6)^*k*^

a Data are presented as *n* (%) unless otherwise specified. SD, standard deviation. Data are missing from up to 2 women and 3 men for some questions.

b Country of birth for females not born in Australia: WHO European region (*n* = 8), WHO Western Pacific region (*n* = 3), WHO Americas region (*n* = 3), WHO South-East Asian region (*n* = 1), WHO Eastern Mediterranean region (*n* = 1).

c Country of birth for males not born in Australia: WHO European region (*n* = 8), WHO Region of the Americas (*n* = 1), WHO Western Pacific region (*n* = 1).

d Three women reported using a contraceptive implant and one reported using a hormonal injection (Depo Provera).

e The three women who reported current douching reported douching daily.

f Includes the partner they enrolled with.

g Discrepancies are a result of independent reporting by female and male partners.

h Metronidazole (*n* = 3), tinidazole (*n* = 1), doxycycline (*n* = 1), azithromycin (*n* = 1).

i Doxycycline (*n* = 1), amoxicillin (*n* = 1), amoxicillin-clavulanic acid (*n* = 1), azithromycin (*n* = 1).

j Clotrimazole (*n* = 2).

k Clotrimazole (*n* = 1), daivobet 50/500 gel (contains calcipotriol; betamethasone dipropionate, *n* = 1).

Clinical and laboratory characteristics are in Table 2. A total of 33 (97%) women had ≥3 Amsel criteria and a Nugent score (NS) of 4 to 10 at enrollment. The final woman had BV by Nugent criteria (NS = 9), as well as presence of clue cells and vaginal pH of >4.5. However, the clinician was unable to accurately assess vaginal discharge and vaginal malodor on examination, as the woman had undertaken intravaginal cleaning immediately prior to examination. Self-reported penile symptoms were reported by 2 males (6%).

**TABLE 2:** Clinical and laboratory characteristics at baselines^*a*^

Characteristic	Data for:	
	Females (*n* = 34)	Males (*n* = 34)
Self-reported vaginal discharge		
No	6 (19)	
Yes	26 (81)	
Self-reported vaginal malodor		
No	6 (18)	
Yes	27 (82)	
Nugent score		
4–6	6 (18)	
7–10	28 (82)	
Amsel criteria		
2	1 (3)^*b*^	
≥3	33 (97)	
Days since LNMP ended [median (IQR)]^*c*^	14 (6–17)	
Self-reported penile discharge		
No		31 (97)
Yes		1 (3)
Self-reported penile malodor		
No		30 (94)
Yes		2 (6)

a Data presented as *n* (%) unless otherwise specified. LNMP, last known menstrual period.

b This woman had BV by Nugent criteria (NS = 9), as well as presence of clue cells and a vaginal pH of >4.5. However, the clinician recorded that vaginal discharge and vaginal malodor (i.e., amine test) were not able to be accurately reported, as the woman had undertaken intravaginal cleaning immediately prior to clinical examination.

c LNMP missing for *n* = 3 women; *n* = 5 women report not menstruating due to hormonal contraception; *n* = 3 women were menstruating at time of BV diagnosis.

### Adherence and tolerability.

All 34 women provided adherence and tolerability data; 32 were prescribed oral metronidazole (400 mg twice daily [BID] for 7 days), and two received 2% intravaginal clindamycin cream (one applicator vaginally for 7 nights; Table 3). All males received both oral metronidazole (400 mg BID for 7 days) and 2% clindamycin cream (applied topically to the penis BID for 7 days). Self-reported adherence to metronidazole was high; 29 women (91%) and 30 men (88%) took all tablets. Both women who received intravaginal clindamycin reported applying all doses, and 24 males (71%) reported applying all clindamycin doses. A total of 18 women and 11 men reported at least one adverse event in the day-8 questionnaire, with nausea (*n* = 13) and metallic taste (*n* = 11) being the most common. Three men reported penile irritation and/or redness, which was mild and not treatment limiting.

**TABLE 3:** Treatment adherence and adverse effects^*a*^

	Female (*n* = 34)	Male (*n* = 34)
Prescribed metronidazole (oral)^*b*^	32 (94)	34 (100)
Self-reported adherence to metronidazole		
Took all tablets	29 (91)	30 (88)
Missed 1–4	1 (3)	3 (9)
Missed >4	2 (6)	1 (3)
Prescribed clindamycin (topical)^*b*^	2 (6)	34 (100)
Self-reported adherence to clindamycin		
Applied all doses	2 (100)	24 (71)
Missed 1–4	0	8 (23)
Missed >4	0	2 (6)
Self-reported adverse effect^*c*^		
Nausea	9 (26)	4 (12)
Vomiting	0 (0)	0 (0)
Metallic taste	7 (21)	4 (12)
Headache	6 (18)	2 (6)
Vaginal irritation	2 (6)	
Irritation of penile skin		2 (6)
Redness of penile skin		2 (6)
Other	8 (23)^*d*^	6 (18)^*e*^

a Data presented as *n* (%) unless otherwise specified.

b Oral metronidazole was the standard treatment for females. One woman received 28 days of metronidazole. Oral metronidazole was contraindicated in two women who subsequently received vaginal clindamycin.

c No adverse effects were reported by women who used clindamycin.

d Other side effects: thrush (*n* = 2), drowsiness or fatigue (*n* = 2), feeling weak (*n* = 1), vaginal dryness (*n* = 1), gastrointestinal upset (*n* = 1), tension behind eyes and feeling ill (*n* = 1).

e Other side effects: fatigue (*n* = 2), dizziness (*n* = 1), tingling sensation in hands (*n* = 1), increased appetite (*n* = 1), and thrush (*n* = 1; this participant also reported irritation and redness of penile skin).

### BV recurrence over the study period.

Of the 29 women to provide follow-up data to study endpoint (BV recurrence defined as NS of 4 to 10 with ≥3 Amsel criteria or week 12 without recurrence), 5 (17%; 95% confidence interval [CI], 6 to 34%) experienced recurrence. Of the five women to experience recurrence, one was diagnosed with recurrence at week 4, two at an interim visit between week 4 and week 8, one at week 8, and one at week 12. Table 4 presents participant practices over the study period stratified by BV recurrence. Women who recurred all had a history of BV, all had an uncircumcised partner, all reported condomless vaginal sex after the 7-day treatment period, and three of the five had an IUD *in situ* (two had a copper IUD *in situ* and one had a levonorgestrel IUD *in situ*). One woman who recurred also reported a new sexual partner during follow-up. All couples that recurred reported 100% treatment adherence, and all reported no sexual contact, or using condoms for sex, during the 7-day treatment period.

**TABLE 4:** Baseline and longitudinal characteristics of women stratified by BV recurrence status

Characteristic	Data for [*n* (%)]:	
	Cured (*n* = 24)	BV recurrence (*n* = 5)
Baseline characteristics		
History of BV		
No	5 (21)	0 (0)
Yes	19 (79)	5 (100)
Circumcised partner		
No	19 (79)	5 (100)
Yes	5 (21)	0 (0)
Intrauterine device *in situ*		
No	16 (67)	2 (40)
Yes	8 (33)^*a*^	3 (60)^*b*^
Current hormonal contraception use		
No	11 (46)	3 (60)
Yes	13 (54)	2 (40)
Treatment period characteristics (days 1–7)		
Female adherence to treatment^*c*^		
100%	21 (88)	5 (100)
<100%	3 (13)	0 (0)
Male adherence to treatment^*d*^		
100%	14 (58)	5 (100)
<100%	10 (42)	0 (0)
Condomless vaginal sex during treatment period		
No sex/sex with a condom only	21 (88)	5 (100)
Yes	3 (13)	0 (0)
Any oral sex received		
No	22 (92)	5 (100)
Yes	2 (8)	0 (0)
Longitudinal posttreatment characteristics (day 8 to endpoint)		
Any condomless vaginal sex		
No sex/protected sex only	1 (4)	0 (0)
Yes	23 (96)	5 (100)
Any condomless anal sex		
No sex/protected sex only	16 (67)	3 (60)
Yes	8 (33)	2 (40)
Any oral sex received		
No	4 (17)	1 (20)
Yes	20 (83)	4 (80)
Any new sexual partner in relationship^*e*^		
No	21 (92)	4 (80)
Yes	3 (8)	1 (20)

a Four women reported using a copper IUD; four reported using a levonorgestrel IUD.

b Two women who recurred reported using a copper IUD, and one reported using a levonorgestrel IUD.

c Refers to treatment adherence for females as self-reported by females in the day-8 questionnaire.

d Refers to treatment adherence for males as self-reported by males in the day-8 questionnaire.

e Three women and one man reported a new sexual partner during the follow-up period.

### Vaginal, cutaneous penile, and urethral microbiota composition at baseline.

A total of 27 couples (including 4 couples where the female partner experienced recurrence and 23 couples where the female partner was cured) were included in microbiota analyses (Fig. 1), providing 131 vaginal specimens, 122 cutaneous penile specimens, and 122 urethral specimens. After quality filtering, the median number of reads per specimen was 24,284 (IQR, 18,988 to 28,719) for vaginal specimens, 22,695 (IQR, 17,698 to 27,046) for cutaneous penile, and 22,497 (IQR, 17,621 to 26,018) for urethral specimens.

**FIG 2:**
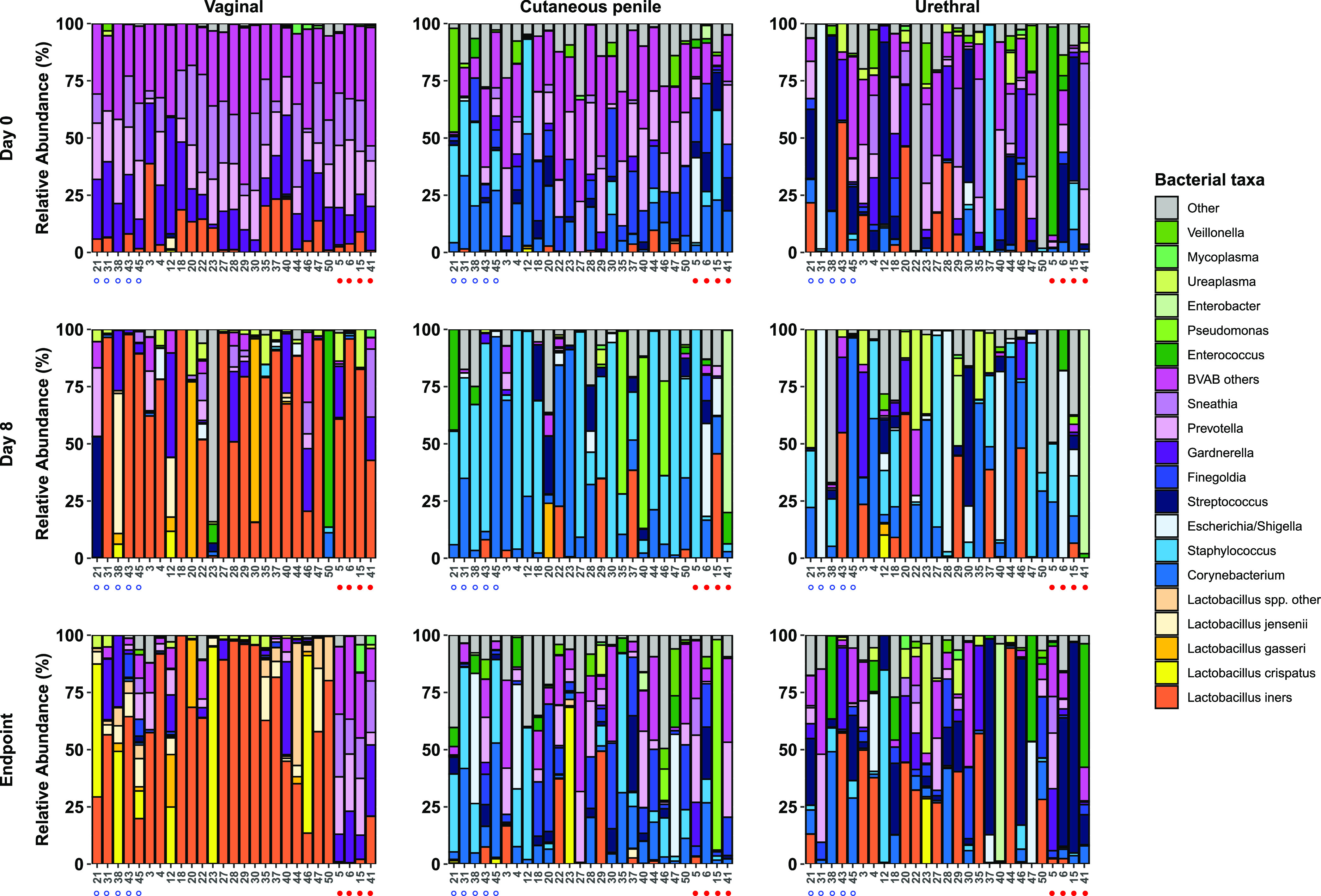
Stacked bar graphs of the vaginal, cutaneous penile, and male urethral microbiota. Stacked bar graphs show the relative abundance of key bacterial taxa in the vaginal, cutaneous penile, and urethral microbiota of sexual partners at day 0, day 8, and the endpoint. Specimens are ordered by couple number. Couples where the male is circumcised are indicated by an open blue circle under the bar graphs, and couples where the woman experienced BV recurrence are indicated by a filled red circle. “*Lactobacillus* spp. other” includes L. antri, L. casei, L. coleohominis, L. fermentum, and L. pontis, and *Lactobacillus* species that were unable to be classified to the species level. “BVAB others” includes less abundant species and genera that have previously been associated with BV (*Aerococcus*, *Anaerococcus*, *Atopobium*, BVAB TM7, “*Candidatus* Lachnocurva vaginae” (previously BVAB-1), BVAB-2, Mageeibacillus indolicus (previously BVAB-3), *Dialister*, DNF00809, *Fusobacterium*, *Gemella*, *Megasphaera*, *Mobiluncus*, *Parvimonas*, *Peptoniphilus*, *Peptostreptococcus*, *Porphyromonas*). The remaining taxa are grouped in the “other” category.

Prior to commencing treatment (i.e., at day 0), all women had low relative abundance of *Lactobacillus* spp. and high prevalence and relative abundance of BV-associated bacteria (Fig. 2 and Fig. S1A). Male specimens were heterogeneous in composition at day 0 (Fig. 2 and Fig. S1B). Overall, the most abundant organisms in the cutaneous penile microbiota were *Corynebacterium*, *Finegoldia*, Staphylococcus, *Peptoniphilus* (grouped under BVAB others in Fig. 2) and *Prevotella*. The most abundant organisms in the urethral microbiota were Streptococcus, Lactobacillus iners, *Gardnerella*, *Sneathia* and Staphylococcus. *Gardnerella* was also prevalent in cutaneous penile samples but at a lower average relative abundance than in the urethra (1.4% versus 7.5%).

10.1128/mBio.02323-21.8 FIG S1 Heatmap of female and male genital specimens. (A) A heatmap of relative abundance of bacterial taxa in all vaginal specimens, ordered by study stage (baseline, day 8, longitudinal [BV recurrence] and longitudinal [no recurrence]). Specimens collected at the time of BV recurrence (defined as ≥3 Amsel criteria and a Nugent score of 4 to 10) are indicated in black in the top metadata bar. Ward linkage clustering was applied to determine the order of specimens within each study stage. VALENCIA (81) was used to assign community state types (CSTs) to vaginal specimens as follows: CST I, dominated by L. crispatus; CST II, dominated by L. gasseri; CST III, dominated by *L. iners*; CST IV-A, low relative abundance of *Lactobacillus* spp. with high to moderate abundance of “*Candidatus* Lachnocurva vaginae” (BVAB-1) and *Gardnerella*; CST IV-B, low relative abundance of *Lactobacillus* spp. with high to moderate abundance of *Gardnerella* and *A. vaginae*; CST IV-C, low relative abundance of *Lactobacillus* spp., “*Candidatus* Lachnocurva vaginae,” and *Gardnerella*; CST V, dominated by L. jensenii. The heatmap was drawn using ComplexHeatmap (82). The Nugent score is also indicated above the heatmap (missing for *n* = 1 specimen). (B) The heatmap of relative abundance of bacterial taxa in all male specimens, ordered by specimen type (cutaneous penile, urethral) and then by study stage (baseline, day 8, longitudinal [BV recurrence in FSP (female sexual partner)], longitudinal [no recurrence in FSP]). Specimens collected at the time of BV recurrence in the female partner are indicated in black in the top metadata bar. Circumcision status is also indicated above the heatmap. Ward linkage clustering was applied to determine the order of specimens within each study stage, and the heatmap was drawn using ComplexHeatmap (82). Download FIG S1, PDF file, 0.4 MB. Copyright © 2021 Plummer et al. 2021 Plummer et al. https://creativecommons.org/licenses/by/4.0/ This content is distributed under the terms of the Creative Commons Attribution 4.0 International license.

### Impact of concurrent partner treatment on the composition of the female and male genital microbiota.

**FIG 3:**
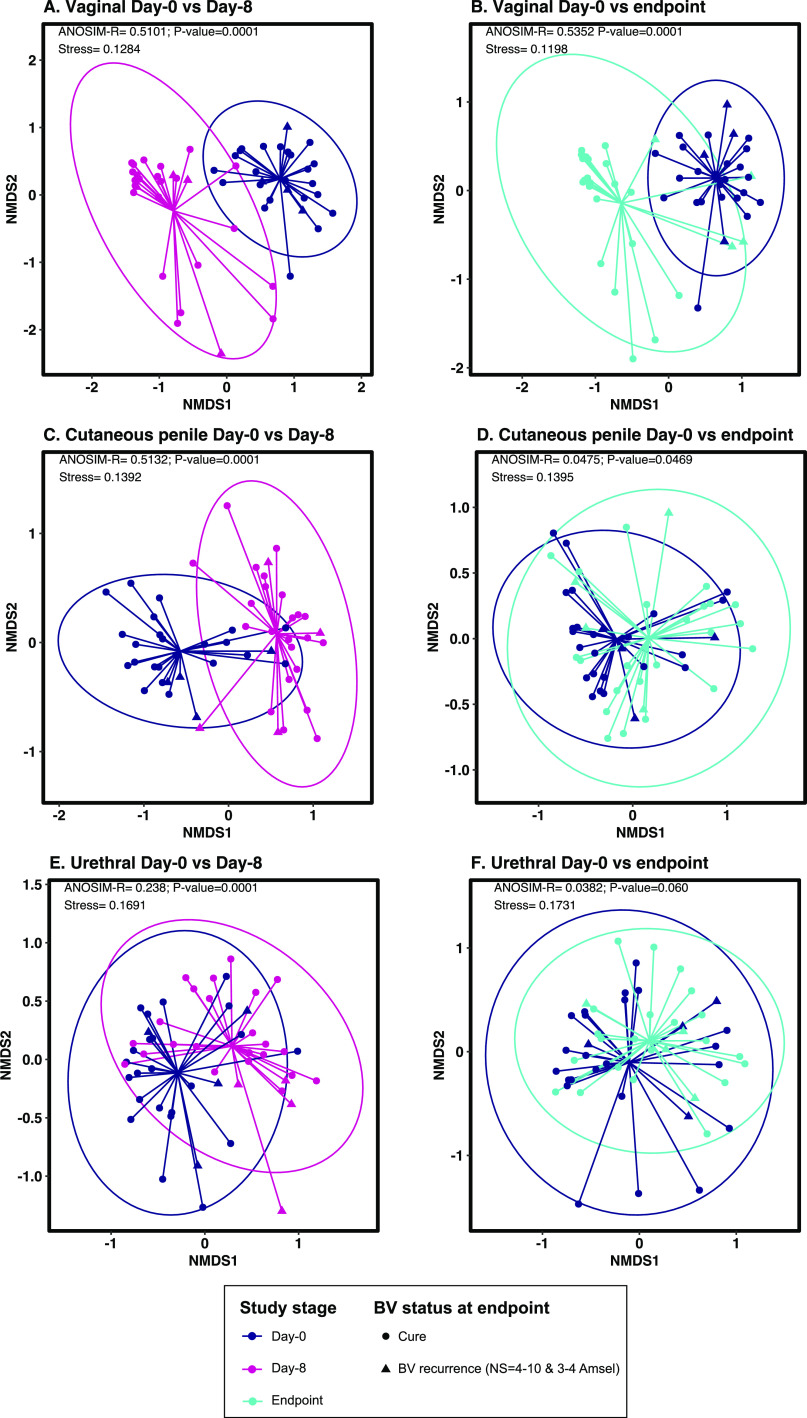
Nonmetric multidimensional scaling (NMDS) plots showing the global differences in microbiota composition following concurrent partner treatment. (A and B) Concurrent partner treatment had a significant immediate (A) and sustained (B) effect on the vaginal microbiota composition. (C to F) Conversely, concurrent partner treatment had a significant immediate effect on the cutaneous penile (C) and urethral microbiota (D), but this was not sustained at endpoint at either male site (E and F). Analysis of similarity (ANOSIM) test statistics are shown in the top left corner of each plot, and a *P* value of <0.05 indicates dissimilarity in the composition of specimens collected pretreatment versus posttreatment. NMDS and ANOSIM analyses were conducted using Bray-Curtis dissimilarities and ASV-level data.

Nonmetric multidimensional scaling (NMDS) analyses revealed that vaginal specimens collected at day 0 clustered separately from those collected immediately following the 7-day treatment period, i.e., at day 8 (analysis of similarity [ANOSIM] *R-*statistic = 0.5101, *P *< 0.001; Fig. 3A), and those collected at the study endpoint, i.e., week 12 or BV recurrence (ANOSIM *R*-statistic = 0.5352, *P *< 0.001; Fig. 3B). Male genital specimens collected at day 0 clustered separately from those collected at day 8 (cutaneous penile ANOSIM *R-*statistic = 0.5132, *P *< 0.001; Fig. 3C and urethral ANOSIM *R*-statistic = 0.238, *P *< 0.001; Fig. 3E). However, male specimens collected at the endpoint did not cluster separately from those collected at day 0, and the ANOSIM *R*-statistics suggest little difference between the overall composition of the male genital microbiota at day 0 compared to that at the endpoint (cutaneous penile ANOSIM *R-*statistic = 0.0475, *P *= 0.05; Fig. 3D and urethral ANOSIM *R-*statistic = 0.0382, *P *= 0.060; Fig. 3F).

**FIG 4:**
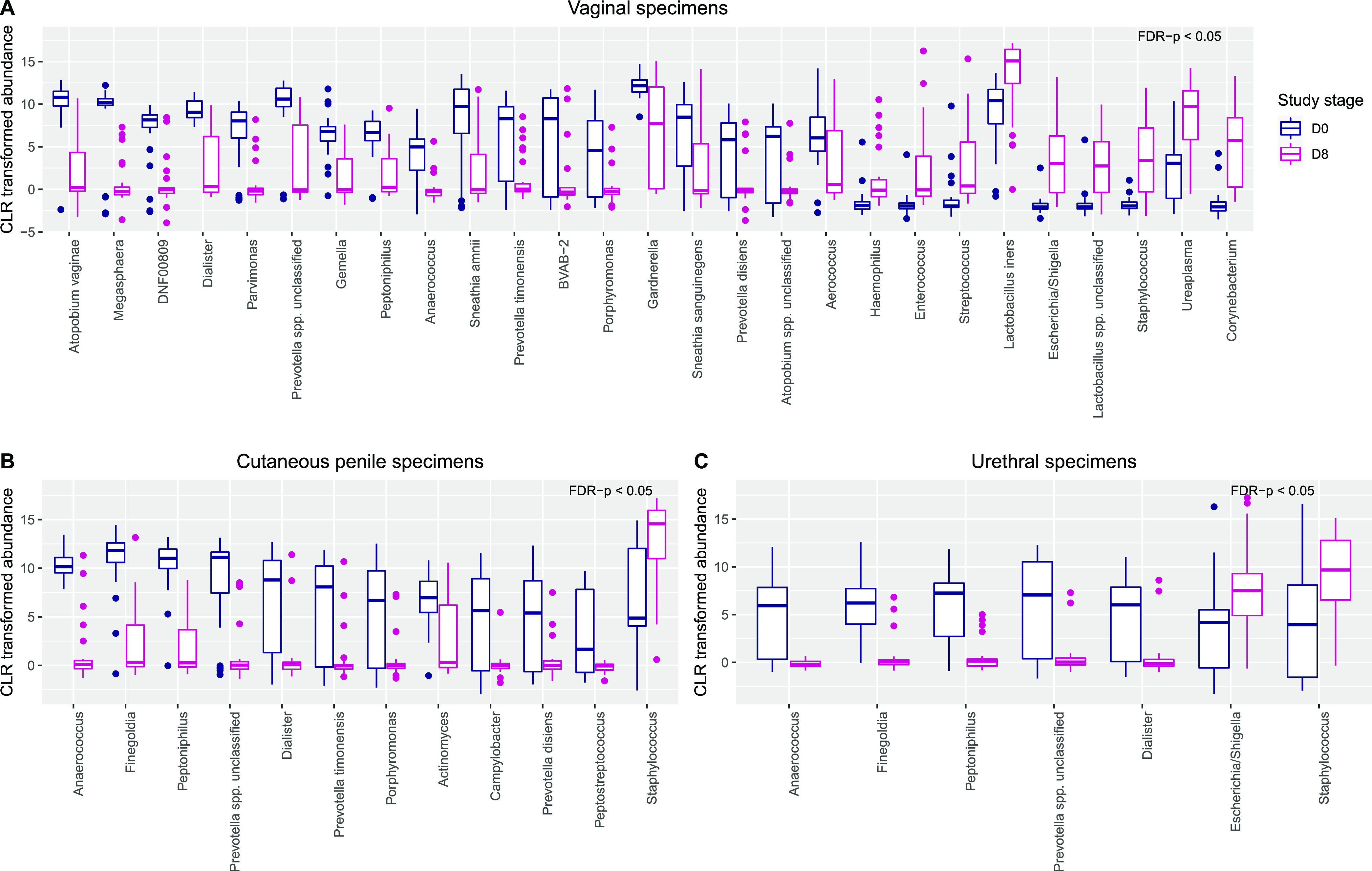
Differences in the relative abundance of taxa between samples collected pretreatment and following 7 days of concurrent partner treatment. (A to C) Boxplots show the centered-log ratio (CLR) transformed relative abundance of bacteria that were differentially abundant by ALDEX2 (FDR < 0.05) between day 0 and day 8 specimens in the vaginal (A), cutaneous penile (B), and urethral (C) microbiota.

The ANOVA-like differential expression tool (ALDEX2) (13, 14) was used to identify taxa that were differentially abundant between pre- and posttreatment specimens, using center log-ratio transformed data (Tables S1, S2 and S3). The relative abundance of 18 taxa was significantly decreased in day 8 vaginal specimens compared to day 0 specimens, including Atopobium vaginae, *Megasphaera*, Coriobacteriales bacterium DNF00809, *Prevotella* spp. (P. timonensis, P. disiens), *Sneathia* spp. (S. amnii, S. sanguinegens), and *Gardnerella* (false-discovery rate [FDR], <0.05; Fig. 4A). Additionally, the relative abundance of nine taxa significantly increased in the vaginal microbiota after 7 days of treatment, including *L. iners*, Staphylococcus, *Ureaplasma*, and *Corynebacterium*. At the study endpoint, the relative abundance of 16 taxa significantly decreased in vaginal specimens compared to day 0, including *A. vaginae*, *Megasphaera*, *Prevotella* spp., *Sneathia* spp., *Coriobacteriales bacterium* DNF00809, and *Gardnerella* (FDR, <0.05; Fig. S2A). *Lactobacillus* spp. (including *L. iners* and L. jensenii), *Corynebacterium*, *Finegoldia*, *Ureaplasma*, and Staphylococcus were significantly increased in vaginal specimens at the endpoint compared to day 0.

10.1128/mBio.02323-21.1 TABLE S1 Taxa that are differentially abundant in vaginal samples in day 0 versus day 8 specimens and day 0 versus endpoint specimens (ALDEX2 analysis). Download Table S1, PDF file, 0.6 MB. Copyright © 2021 Plummer et al. 2021 Plummer et al. https://creativecommons.org/licenses/by/4.0/ This content is distributed under the terms of the Creative Commons Attribution 4.0 International license.

10.1128/mBio.02323-21.2 TABLE S2 Taxa that are differentially abundant in cutaneous penile samples in day 0 versus day 8 specimens and day 0 versus endpoint specimens (ALDEX2 analysis). Download Table S2, PDF file, 0.5 MB. Copyright © 2021 Plummer et al. 2021 Plummer et al. https://creativecommons.org/licenses/by/4.0/ This content is distributed under the terms of the Creative Commons Attribution 4.0 International license.

10.1128/mBio.02323-21.3 TABLE S3 Taxa that are differentially abundant in urethral samples in day 0 versus day 8 specimens and day 0 versus endpoint specimens (ALDEX2 analysis). Download Table S3, PDF file, 0.5 MB. Copyright © 2021 Plummer et al. 2021 Plummer et al. https://creativecommons.org/licenses/by/4.0/ This content is distributed under the terms of the Creative Commons Attribution 4.0 International license.

10.1128/mBio.02323-21.9 FIG S2 Differences in the relative abundance of taxa between samples collected at day 0 and study endpoint. (A to C) Boxplots show the centered-log ratio (CLR) transformed relative abundance of bacteria that were differentially abundant by ALDEX2 between day-0 and endpoint specimens in the vaginal (A) (FDR < 0.05), cutaneous penile (B) (*P* < 0.05 but FDR > 0.05), and urethral (C) microbiota (*P* < 0.05 but FDR > 0.05). Download FIG S2, PDF file, 0.02 MB. Copyright © 2021 Plummer et al. 2021 Plummer et al. https://creativecommons.org/licenses/by/4.0/ This content is distributed under the terms of the Creative Commons Attribution 4.0 International license.

The relative abundance of 11 taxa, including *Anaerococcus*, *Finegoldia*, *Peptoniphilus*, and *Prevotella* spp. and *Dialister*, was significantly decreased in day 8 cutaneous penile specimens compared to those at day 0 (FDR, <0.05; Fig. 4B). The relative abundance of these five taxa was also significantly decreased in the urethral microbiota after 7 days of treatment (FDR, <0.05; Fig. 4C). Additionally, the relative abundance of Staphylococcus significantly increased at day 8 at both male sites, and Escherichia increased at the urethra. The relative abundance of several BV-associated bacteria remained decreased at the cutaneous penile and urethral sites at the endpoint; however, following FDR correction, the difference was not significant (*P *< 0.05 but FDR > 0.05; Fig. S2B and S2C).

### The composition of the female and male genital microbiota by recurrence status.

Analysis of composition of microbiomes (ANCOM) (15) was used to investigate longitudinal differences in microbiota composition between couples in whom the female partner experienced BV recurrence and those who did not. This analysis had limited power to detect differences due to the small number of women who recurred. However, 11 taxa, including *S. sanguinegens*, *Dialister*, *Gemella*, and *S. amnii*, were found in significantly higher abundance in the vaginal microbiota of women who experienced BV recurrence (Table S4). Conversely, Lactobacillus crispatus and Lactobacillus gasseri were present in cured women but were not detected in women who recurred. No taxa were significantly differentially abundant between male partners of women who recurred and male partners of cured women.

10.1128/mBio.02323-21.4 TABLE S4 Analysis of composition of microbiomes (ANCOM) of longitudinal vaginal, cutaneous penile, and urethral specimens from couples that were cured versus couples who recurred. Download Table S4, PDF file, 0.7 MB. Copyright © 2021 Plummer et al. 2021 Plummer et al. https://creativecommons.org/licenses/by/4.0/ This content is distributed under the terms of the Creative Commons Attribution 4.0 International license.

### Correlations between bacterial taxa in the female and male genital microbiota of sexual partners.

We investigated the correlation between vaginal taxa and both cutaneous penile and urethral taxa using FastSpar (16). Seven taxa were positively correlated between sexual partners at day 0 (Table 5), one of which (*S. sanguinegens*) showed moderate positive correlation between the vaginal microbiota and both the cutaneous penile and urethral microbiota (correlation coefficient = 0.37 and 0.43, respectively). *P. disiens* and “*Candidatus* Lachnocurva vaginae” (BVAB-1) were both moderately positively correlated between the vagina and cutaneous penile microbiota (correlation coefficient = 0.47 and 0.63, respectively). Additionally, L. crispatus, L. gasseri, and L. jensenii were moderately positively correlated between the vagina and cutaneous penile microbiota (correlation coefficient = 0.57, 0.48, and 0.35, respectively). *Dialister* was negatively correlated between the vaginal microbiota and both the cutaneous penile and urethral microbiota. At day 8, four taxa were moderately positively correlated between the vaginal and cutaneous penile microbiota of sexual partners (*Anaerococcus*, L. gasseri, *Finegoldia*, and *Corynebacterium*), and two were moderately positively correlated between the vaginal and urethral microbiota (L. crispatus and L. gasseri).

**TABLE 5:** Correlation of specific bacterial taxa between the genital microbiota of sexual couples at day 0, day 8, and longitudinally^*a*^

Taxon	Day 0^*b*^				Day 8^*c*^				Longitudinal samples^*d*^			
	Vaginal/penile^*e*^		Vaginal/urethral		Vaginal/penile^*e*^		Vaginal/urethral		Vaginal/penile^*e*^		Vaginal/urethral	
	Corr	*P* value	Corr	*P* value	Corr	*P* value	Corr	*P* value	Corr	*P* value	Corr	*P* value
*Aerococcus#*	0.1636	0.3916	0.0737	0.7283	0.1109	0.5085	0.3201	0.0549	**0.4118**	**0.001**	0.2678	0.017
*Anaerococcus*	0.3021	0.1009	0.1247	0.5095	**0.4041**	**0.005**	–	–	0.1335	0.2797	−0.1874	0.1069
Atopobium vaginae	0.0732	0.7293	0.0293	0.8931	−0.1365	0.3596	0.1617	0.2368	0.048	0.4795	0.1367	0.0989
“*Candidatus* Lachnocurva vaginae” (BVAB-1)*#*	**0.6268**	**0.001**	0.2533	0.0619	–	–	–	–	–	–	–	–
BVAB-2*#*	0.2931	0.0889	**0.3586**	**0.048**	0.0215	0.8641	–	–	−0.0145	0.8342	0.1691	0.046
*Corynebacterium*	0.1236	0.3716	0.1833	0.1908	**0.3943**	**0.029**	0.1698	0.3976	**0.3786**	**0.001**	−0.167	0.1489
*Dialister#*	**−0.4451**	**0.017**	**−0.5567**	**0.004**	0.2804	0.0759	0.0238	0.8721	0.2419	0.036	0.1691	0.1259
Enterobacter	0.1032	0.3696	0.1894	0.1032	–	–	–	–	–	–	–	–
*Enterococcus*	0.0912	0.4396	0.047	0.6773	0.0294	0.8731	−0.0732	0.6733	**0.339**	**0.001**	0.2269	0.015
Escherichia */Shigella*	0.201	0.0909	0.1704	0.1768	0.2311	0.2168	0.0596	0.7293	0.2444	0.017	0.0725	0.5175
*Finegoldia*	−0.2017	0.2707	−0.0037	0.984	**0.4306**	**0.004**	0.0416	0.7812	−0.0385	0.7433	−0.1229	0.2947
*Gardnerella*	0.0522	0.7862	0.0522	0.7862	−0.0434	0.8212	−0.0025	0.991	**0.3473**	**0.004**	**0.3109**	**0.012**
L. crispatus	**0.5724**	**0.001**	**–**	**–**			**0.4517**	**0.008**	**0.4627**	**0.001**	**0.3291**	**0.002**
L. gasseri	**0.4813**	**0.001**	–	–	**0.4229**	**0.01**	**0.3835**	**0.013**	**0.4362**	**0.001**	0.2223	0.014
*L. iners*	0.1843	0.3407	0.2438	0.2168	0.0901	0.6523	0.1727	0.3726	**0.3654**	**0.003**	0.2876	0.011
L. jensenii	**0.3522**	**0.032**	–	–	–	–	0.2595	0.044	**0.4082**	**0.001**	0.1531	0.037
*Megasphaera#*	0.1845	0.3387	0.0627	0.6314	–	–	–	–	−0.0105	0.8771	–	–
*Peptoniphilus*	0.1153	0.5764	−0.3671	0.0559	0.2428	0.1499	−0.1205	0.3976	0.0869	0.4615	−0.1136	0.3257
*Prevotella* unclassified spp.	0.2522	0.1738	0.1586	0.4585	0.2649	0.1039	−0.0937	0.5445	0.2082	0.0869	0.1823	0.1189
*P. bivia*	0.0381	0.8382	0.0042	0.974	0.0324	0.8122	0.0308	0.7902	0.1859	0.0969	0.0682	0.5984
*P. disiens#*	**0.4698**	**0.01**	0.1844	0.3437	−0.1425	0.2667	–	–	**0.3583**	**0.001**	0.2828	0.011
*P. timonensis#*	0.3343	0.0759	0.1234	0.4975	0.1364	0.3636	−0.1007	0.3676	**0.4585**	**0.001**	**0.3742**	**0.002**
Pseudomonas	0.1844	0.0972	−0.0169	0.8824	–	–	–	–	0.0782	0.2148	0.0642	0.3666
*Sneathia* unclassified spp. *#*	0.2488	0.1558	**0.3806**	**0.026**	–	–	–	–	0.0154	0.8202	0.159	0.0689
*S. amnii*	0.3132	0.0729	0.3036	0.1209	0.1869	0.2068	0.1051	0.4505	**0.3932**	**0.001**	**0.4796**	**0.001**
*S. sanguinegens*	**0.3741**	**0.038**	**0.4302**	**0.019**	0.1865	0.1928	–	–	**0.365**	**0.005**	**0.3234**	**0.005**
Staphylococcus	0.2183	0.0619	0.1325	0.2577	0.1502	0.4525	0.1026	0.5864	**0.3088**	**0.011**	0.02	0.8691
Streptococcus	0.1469	0.3836	0.1593	0.3417	0.0903	0.6154	0.0874	0.6174	0.1882	0.0999	0.0575	0.6384
*Ureaplasma*	−0.0355	0.8182	−0.0416	0.8272	−0.1423	0.4446	0.1696	0.4006	0.1087	0.3397	0.1945	0.0849

a Corr, SparCC correlation coefficient; – indicates that the taxon was not detected in one or more specimen types for that study time point. The 10 most abundant bacteria at each site and 8 bacteria previously associated with BV (indicated by *#*) are presented in this table. Correlations with an absolute correlation coefficient of >0.3 and *P *value of <0.05 were considered significant and have been set in bold type in this table.

b Includes 27 vaginal, 27 cutaneous penile, and 27 urethral specimens from 27 couples.

c Includes 27 vaginal, 27 cutaneous penile, and 27 urethral specimens from 27 couples.

d Includes 68 vaginal, 68 cutaneous penile, and 68 urethral specimens from 27 couples.

e Penile refers to cutaneous penile specimens.

We also observed a significant positive correlation of taxa between sexual partners longitudinally. When we stratified data by BV recurrence (Table S5), two taxa were significantly correlated between the vaginal and urethral microbiota of sexual partners who recurred; *P. timonensis* was strongly positively correlated (correlation coefficient = 0.97) and *L. iners* was strongly negatively correlated (correlation coefficient=−0.96). *S. amnii* also demonstrated a strong positive correlation between the vaginal and urethra microbiota of sexual partners that recurred, but the correlation was not significant (correlation coefficient = 0.85, *P *= 0.06). Conversely, *Lactobacillus* spp. (including L. crispatus, *L. iners*, L. gasseri, and L. jensenii), *Corynebacterium*, and *Gardnerella* were moderately positively correlated between the genital microbiota of cured partners. Correlation of taxa predominately occurred between the vaginal and cutaneous penile sites; however, L. crispatus and *L. iners* were also correlated between the vagina and male urethra. The BV-associated bacteria *P. timonensis* and *S. amnii* were also moderately positively correlated between the genital microbiota of cured partners (correlation coefficient = 0.38 and 0.34, respectively), but the strength of correlation between cured couples was lower than that of couples that recurred. As discussed above, these analyses had limited power to detect differences due to the small number of women who recurred.

10.1128/mBio.02323-21.5 TABLE S5 Correlation of specific bacterial taxa between the genital microbiota of sexual partners longitudinally, stratified by BV recurrence status. Download Table S5, PDF file, 0.5 MB. Copyright © 2021 Plummer et al. 2021 Plummer et al. https://creativecommons.org/licenses/by/4.0/ This content is distributed under the terms of the Creative Commons Attribution 4.0 International license.

## DISCUSSION

In this pilot study of combined oral and topical antibiotic treatment of male partners of women being treated for BV we assessed (i) the impact of concurrent partner treatment on the composition of the vaginal, cutaneous penile, and male urethral microbiota over a 12-week period and (ii) the adherence to and acceptability and tolerability of concurrent partner treatment and (iii) generated preliminary estimates of the efficacy of the intervention over a 12-week period. We found that concurrent partner treatment significantly altered the overall composition of the genital microbiota of both partners immediately following treatment. Specifically, we observed a decrease in the abundance of anaerobic BV-associated bacteria at all three urogenital sites. Several taxa, including *Lactobacillus* spp., *Corynebacterium*, and *Ureaplasma*, increased in abundance in the vaginal microbiota, and the abundance of Staphylococcus increased in both the cutaneous penile and urethral microbiota. However, by 12 weeks, the overall composition of the male genital microbiota was not significantly different from baseline, with BV-associated bacteria reemerging at both male sites. Despite this, the majority of women experienced suppression of BV-associated bacteria, a sustained increase in *Lactobacillus* spp. (most commonly, *L. iners*), and BV cure to 12 weeks. This study was not powered to assess the effect of partner treatment on BV recurrence; however, we observed fewer than expected cases of BV recurrence in a group of women with a high prevalence of risk factors for recurrence. Additionally, we found that concurrent partner treatment was well tolerated and adhered to by men. Close to 90% of approached male partners agreed to participate in the study and receive the intervention, most reported 100% adherence to treatment, and few side effects were reported. These findings extend the data from our previous exploratory study (8) and provide the support and evidence to proceed to a randomized controlled trial (RCT) of combined oral and topical male partner treatment for BV (17).

Recurrence following recommended BV treatments is unacceptably high. Prior studies have shown that approximately 20 to 25% of women recur within 1 month of female-only treatment, and up 50% recur within 12 weeks (3, 4). Notably, women with an RSP have a 2- to 3-fold increased risk of recurrence (3, 5, 6), and the recent RCT of partner treatment for BV conducted by Schwebke et al. (18) reported recurrence to be as high as 80% in women (all of whom had an RSP), in the 16 weeks following standard female-only treatment. All women in our study had an RSP, and other known risk factors for recurrence were very common; 80% of women had a history of BV, 80% of RSPs were uncircumcised, and all but one couple reported condomless sex during the follow-up period. Additionally, 35% of women had an IUD *in situ*, and IUD use, particularly copper IUD use, has been associated with increased risk of BV (19–23). Given the risk profile of our population, we would expect recurrence rates in our cohort to be similar to that reported by Schwebke et al. (18). Encouragingly, only five women (17%) experienced recurrence within 12 weeks following concurrent partner treatment, and 24 (83%) were cured at 12 weeks posttreatment. Additionally, although our first exploratory study assessed BV using the Nugent score only (8), pooled data from our two studies provide an insight into 1-month recurrence rates following this intervention. Collectively, of 50 couples undergoing partner treatment who provided data to 4 weeks, only 3 women (8%; 95% CI, 2 to 19%) experienced recurrence within 1 month of treatment.

Despite the encouraging results from our two studies, past randomized trials of male partner treatment have failed to consistently improve BV cure (18, 24–29). Methodological limitations have been highlighted as a potential reason that early trials failed (30, 31); however, the recent study by Schwebke et al. (18), a well-designed placebo-controlled trial of male partner treatment with 7 days of oral metronidazole, also failed to improve BV cure. A common characteristic of completed trials is that they have all used only oral therapy for men. This contrasts to our study, which used combined oral and topical antibiotics for men, specifically to address multisite carriage of BV-associated bacteria in men (9–12). In addition, topical treatment may be particularly important in uncircumcised males, who have a high abundance of anaerobic BV-associated bacteria in the penile microbiota compared to circumcised men (11, 12). Circumcision has been shown to reduce the abundance of anaerobic bacteria in the penile microbiota (32, 33) and has been associated with a reduced risk of BV in female partners (34). Therefore, it is possible that circumcision may impact not only a woman’s risk of BV recurrence, but also the effectiveness of male partner treatment strategies. While the small sample size and small number of circumcised men in this pilot study limits any investigation of the impact of circumcision on the efficacy of partner treatment, we will address this in our RCT, as randomization to male partner treatment is stratified by circumcision status (17).

Overall, there is a lack of information on how antibiotics modify the male genital microbiota composition, with our two studies providing the only data. A currently recruiting trial (ClinicalTrials registration number NCT03412071) is investigating the impact of four antimicrobial agents (oral tinidazole, topical metronidazole, topical clindamycin, and topical hydrogen peroxide) compared to circumcision on the composition of the cutaneous penile microbiota (35) and will add to our understanding of how different antimicrobials modify the penile microbiota composition. Investigating alternative oral antimicrobials may be of particular importance because, consistent with our first exploratory study (8), we observed lower male adherence to clindamycin compared to metronidazole, potentially indicating a preference for oral over topical therapy. However, qualitative data from our group suggests that men largely felt that both the oral and topical treatment were easy to use (36). Although the five women who recurred in our study all self-reported 100% treatment adherence, as did their male partners, nonadherence was a predictor of recurrence in the study by Schwebke et al. (18), which reported 100% adherence to metronidazole in 71% of females and 64% of males. These data highlight the importance of education and emphasis on strategies to optimize adherence for males. These data also highlight the importance of continued investigation to determine the acceptability of alternative antimicrobials for partner treatment and if there is any preference among men for oral versus topical treatment.

In both of our partner treatment studies completed to date we observed reemergence of BV-associated bacteria in men that was not associated with BV recurrence in female partners. These data raise important questions. First, why is the immediate effect of treatment on the male genital microbiota not sustained? Although the immediate effect of treatment on the cutaneous penile microbiota was in the same order of magnitude as that of the vagina (ANOSIM *R-*statistics = 0.5132 and 0.5101, respectively), the immediate effect of treatment on the urethral microbiota was not as large (ANOSIM *R-*statistic= 0.238). These data may suggest reduced treatment efficacy at the urethral site. Intrinsic resistance to metronidazole is well documented for BV-associated bacteria, including *Gardnerella* and *A. vaginae* (37–43), and studies have shown that *Gardnerella* is less resistant to clindamycin than metronidazole (41, 44). Additionally, BV-associated biofilms have been detected in male urine (45), and bacterial biofilms comprised predominately of *Gardnerella* and *Atopobium* have been shown to reemerge within 3 weeks of treatment with oral metronidazole (46). Together, these data may suggest that metronidazole-resistant BV-associated bacteria persist at low levels posttreatment in the urethra, potentially in the setting of an established biofilm, only to reemerge later at both male sites. An alternative hypothesis is that sequestration of metronidazole by nontarget organisms such as *Lactobacillus* spp. may impact metronidazole efficacy (47). Lee et al. reported that women with BV were more likely to fail metronidazole if they had a low pretreatment ratio of BV-associated bacteria to *Lactobacillus* spp. (47), suggesting the pretreatment vaginal microbiota composition may impact treatment efficacy. It is unclear what implications this has for metronidazole efficacy in men and if organisms that are present in high abundance in the male genital microbiota pretreatment may influence treatment outcomes. The reemergence of BV-associated bacteria in men may suggest that alternative treatment(s), or a longer duration of treatment, could be needed to achieve sustained clearance of these organisms from men. Finally, it is possible that BV-associated bacteria are reintroduced into the male genital microbiota from extrapenile sources (i.e., the prostate [9] or the gastrointestinal tract) via autoinoculation or during anal sex.

Little is known about what constitutes an optimal or “normal” genital microbiota in men. A study of 18 adolescent males reported detection of BV-associated bacteria, including *Atopobium*, *Megasphaera*, *Mobiluncus*, *Prevotella*, and *Gardnerella* in the coronal sulcus of sexually experienced and sexually inexperienced individuals (11). Although BV-associated bacteria are commonly detected in the genital microbiota of male partners of women with BV, their detection has also been reported in male partners of women without BV, albeit at lower abundance and prevalence (10, 48). In line with the evidence that specific *Gardnerella* spp. or clades associate with BV and others do not (49–54), it is possible that the organisms that reemerged in men are nonvirulent species or strains that constitute a normal male genital microbiota, or do not pose a BV risk to their female partner. Interestingly, a recent study reported that six bacterial species present in the penile foreskin microbiota (belonging to the *Prevotella*, *Dialister*, and *Peptostreptococcus* genera), were associated with increased risk of HIV acquisition in men (55). Some of the identified species (i.e., Prevotella bivia, *P. disiens*) have previously been associated with BV and were also identified in our study. While a low diversity *Lactobacillus*-dominant vaginal microbiota has generally been accepted as associated with optimal outcomes in women, we have no such data in males or knowledge of if there is an optimal genital microbiota in men. Further research is clearly needed to better understand the composition of the genital microbiota in males and to determine the contribution of BV-associated bacteria to health outcomes in men.

The second question our data raise is why does the return of BV-associated bacteria in men not correspond to a return of BV-associated bacteria and subsequent recurrence in all women? One hypothesis is that the organisms we see reemerge in men are not driving BV pathogenesis. Alternatively, although longitudinal data suggest that the majority of BV recurrence occurs within the 12 weeks following treatment (3–6, 18), it is possible that treating male partners delays recurrence in women, and we were unable to capture this within 3 months of follow-up. However, one would expect the effect on women, of a 1-week intervention in males, to be captured and most relevant to the first 1 to 2 months of follow-up. Another potential explanation is that the immediate reduction of BV-associated bacteria in both partners is sufficient for *Lactobacillus* spp., which are resistant to metronidazole (56, 57), to recolonize the vagina and provide a barrier to reinfection. Additionally, we found that *Lactobacillus* spp. (including L. crispatus, *L. iners*, L. gasseri, and L. jensenii) were moderately correlated between the genital microbiota of sexual partners who were cured. In contrast, *L. iners* was negatively correlated between partners who recurred, and in general, *Lactobacillus* spp. were depleted among couples that recurred. Although our correlation and ANCOM analyses had limited power due to the small number of recurrences, our findings indicate that couples who share *Lactobacillus* spp. may be more likely to maintain an optimal vaginal microbiota and less likely to experience recurrence. In support of this, increased frequency of penile-vaginal sex has been shown to increase the concentration of hydrogen peroxide-producing lactobacilli (58). This is also supported by studies of female partnerships that have shown that female sexual partners share *Lactobacillus* strains (59) and are highly concordant for Nugent score category (60–63).

If concurrent partner treatment is shown to be effective for reducing BV recurrence, its success depends on its acceptability to couples. Eleven of the women we approached declined to participate because they did not want to discuss BV or the study with their partner or felt that he would not be interested in participating. Excluding these couples biases our findings toward a higher proportion of male partners accepting treatment; however, we do not know for sure if these men would have declined. Importantly, among couples that discussed the study, acceptance of partner treatment was high. A recent qualitative study of men participating in partner treatment trials for BV highlighted that open communication in the relationship was a key element to men accepting treatment (36). Furthermore, participants hypothesized that men may decline partner treatment because they feel BV has “nothing to do with them,” and this is compounded by a lack of corresponding symptoms and no diagnostic test for men. Additionally, the nature of the relationship (i.e., casual versus established relationship) is likely to impact on a partner’s willingness to be treated (36). This research identifies key barriers to couples accepting partner treatment and highlights that more support is needed to help women discuss BV with their partners, and more education about BV and male carriage of BV-associated bacteria is needed to effectively engage men.

The limitations of this study include the small size and recruiting from a single sexual health clinic, both of which limit the generalizability of our findings. Additionally, we did not have a control group where men were not treated. Thus, we were unable to compare the effect of concurrent partner treatment to standard treatment (i.e., female-only treatment) or determine if treating a woman with antibiotics influences the genital microbiota of her untreated male partner. Our currently recruiting multisite trial (17) (Australian Clinical Trials registration number ACTRN12619000196145) will provide the first randomized data on the efficacy of combined oral and topical partner treatment for BV compared to female-only treatment. Additionally, there are limitations with 16S rRNA gene sequencing, including reduced resolution beyond the genus level. Although the etiological agent of BV remains unknown, specific species of *Gardnerella* have been hypothesized to be integral to BV pathogenesis (64). As *Gardnerella* species cannot be distinguished using the 16S rRNA gene sequence (65), we could not achieve this level of resolution in this study. Studies are needed to determine the effect of concurrent partner treatment at the strain level, as well as to identify the organisms that represent an optimal genital microbiota composition in men and if there are organisms in men that promote optimal outcomes in female partners. Furthermore, extending the follow-up duration may provide more insight into the durability of male partner treatment for BV, although onset of new partnerships becomes more likely the longer individuals are followed. Finally, there are limitations with self-reported data, including recall bias and social desirability bias (66).

In summary, our findings demonstrate that combined oral and topical antibiotic treatment has a significant and immediate effect on the composition of the female and male genital microbiota. Although our study was not powered to measure BV recurrence, we observed a lower-than-expected BV recurrence within the 12 weeks following concurrent partner treatment in a group of women who were at high risk of recurrence. We would expect >50% of these women to experience BV recurrence within 12 weeks of standard first-line antibiotics, and 17% of women recurred following concurrent partner treatment. Our findings demonstrate that concurrent partner treatment is well adhered to and tolerated by those who agree to receive it. Critically, our study highlights that strategies to educate couples in a way that encourages open communication about BV are needed to ensure partner treatment is well accepted. If shown to be effective in a randomized setting, concurrent partner treatment, utilizing combined oral and topical antibiotic treatment for male partners, represents a readily implementable intervention that, ultimately, may be a strategy to achieve sustained BV cure and improve reproductive and sexual health outcomes for women globally.

## MATERIALS AND METHODS

This study was conducted and reported in accordance with the Transparent Reporting of Evaluations with Nonrandomised Designs statement (67) and was prospectively registered with the Australian New Zealand Clinical Trials Registry (ACTRN12618000219280). Ethical approval was obtained from the Human Research and Ethics Committee of the Alfred Hospital, Melbourne, Australia (project number 264/15). Written informed consent was obtained from all participants.

### Study design, participants, and recruitment.

This was a prospective, open-label pilot study of concurrent partner treatment for BV conducted at the MSHC, Victoria, Australia, from March 2018 to March 2019. This study was originally designed as a two-arm, nonrandomized trial, where participants could choose to be in either an intervention group (concurrent partner treatment) or a standard of care group (female-only treatment). While the study objectives were to obtain acceptability, tolerability, and microbiota data, as well as preliminary efficacy estimates for the intervention, the design enabled couples to enroll if the male declined treatment. However, all recruited couples elected to receive concurrent partner treatment (i.e., no males who agreed to participate in the study declined treatment), so the study is reported as a single-arm study.

Women attending MSHC with vaginal symptoms were tested for BV using both Amsel criteria (68) and the Nugent method (69). In keeping with our standard clinical practice, women were diagnosed with BV defined by ≥3 Amsel criteria and NS of 4 to 10. Women with BV were referred to the research team for eligibility screening. Women were eligible if they were prescribed a first-line treatment for BV (oral metronidazole 400 mg BID for 7 days) or an alternative first-line treatment (i.e., 2% vaginal clindamycin cream as one applicator vaginally for 7 nights if oral metronidazole was contraindicated), were aged 18 to 55 years, were willing and able to comply with study requirements, and had a regular male partner of ≥2 months who was willing to participate. Women were ineligible if they were pregnant or breastfeeding, HIV positive, or diagnosed with current pelvic inflammatory disease, had an allergy to both metronidazole and clindamycin, were diagnosed with Chlamydia trachomatis, Neisseria gonorrhoeae, or Trichomonas vaginalis at baseline, had other current sexual partners, or were engaging in current sex work.

Male partners of women who agreed to participate were screened for eligibility and recruited either in clinic or via a telephone consultation. Males were eligible if they were aged ≥18 years and were willing and able to comply with study requirements. Males were ineligible if they were HIV positive, had an allergy to metronidazole or clindamycin, had other current sexual partners, or were engaging in current sex work.

### Intervention.

All males received oral metronidazole, 400 mg BID, and 2% clindamycin cream which was applied topically to the head of the penis and upper shaft (under the foreskin if uncircumcised) BID for 7 days. Couples were instructed to abstain from sexual activity until both partners had completed all treatment doses.

### Study procedures.

Prior to commencing antibiotics, participants completed a questionnaire concerning demographics, clinical and behavioral information, and self-collected genital specimens for microbiota analysis. Women self-collected a high-vaginal swab (flocked swab; Copan, Italy), and males self-collected a cutaneous penile swab and a first pass urine (FPU) sample. FPU has been shown to provide an accurate representation of the urethral microbiota (70) and was chosen, as it was thought to be more acceptable to men than a self-collected urethral swab. The cutaneous penile swab was collected using a Copan flocked swab premoistened with sterile water. Males were instructed to firmly rub the swab three times around the coronal sulcus and then over the glans of the penis. Males obtained the FPU sample by urinating the first 15 ml of urine into a pot and then used a sterile single-use plastic pipette to transfer the urine to a 15-ml tube with 830 μl of AssayAssure Genelock (SierraMolecular, USA). Males were instructed to retract the foreskin if uncircumcised before collecting both specimens. Female baseline samples were collected in the clinic, and male baseline samples were collected at home.

Couples completed questionnaires and self-collected genital specimens at day 8 (the day after finishing antibiotics) and weeks 4, 8, and 12. At each follow-up time point (i.e., day 8 and weeks 4, 8, and 12), women self-collected a vaginal swab and a smear for Nugent scoring, and men self-collected a cutaneous penile swab and FPU sample. All follow-up was completed at home by participants, and questionnaires and self-collected specimens were returned by post, with the exception that women attended the clinic for a week-12 follow-up visit that included a BV assessment in addition to the questionnaire and self-collected vaginal swab. Women who experienced BV symptoms during follow-up were recalled to the clinic for BV assessment. Couples were censored from the study if the woman experienced BV recurrence (≥3 Amsel criteria and a Nugent score [NS] of 4 to 10). Where the woman had an NS of 7 to 10 during follow-up, she was encouraged to attend the clinic for BV assessment using combined Amsel and Nugent criteria. Women who did not report symptoms and could not attend the clinic were not treated and continued to provide specimens until the study endpoint (defined as BV recurrence within 12 weeks or reaching week 12 without recurrence).

### Outcomes.

The following outcomes were measured: (i) The effect of concurrent partner treatment on the composition of the vaginal and male genital (cutaneous penile and urethral) microbiota over a 12-week period. Couples contributed to this outcome if both partners returned a minimum of day-0, day-8, and endpoint specimens. (ii) Adherence to, and acceptability and tolerability of, combined oral and topical antibiotic treatment in male partners of women with BV. Adherence was self-reported at day 8, acceptability was assessed as the proportion of males who agreed to participate, and tolerability was assessed by self-report of adverse events at day 8. Couples contributed to this outcome if both partners completed the day-8 questionnaire. (iii) Preliminary estimates of BV recurrence (defined as ≥3 Amsel criteria and NS of 4 to 10) over the 12-week study period. Couples contributed to this outcome if both partners completed the day-8 questionnaire and provided questionnaire data to the study endpoint.

### Microbiota characterization.

On receipt, vaginal and cutaneous penile swabs were agitated in 600 μl of phosphate-buffered saline (PBS) and stored at **−**80°C until DNA extraction. For samples returned by post, the median time between samples being self-collected and stored at **−**80°C was 3 days (IQR, 2 to 5 days). DNA was extracted from swab samples using a prelysis bead-beating protocol followed by automated extraction on the MagNA Pure 96 system (Roche Diagnostics, Germany). Samples were prepared for prelysis bead beating as follows: 200 μl of sample, 300 μl of MagNA Pure 96 bacterial lysis buffer (Roche Diagnostics), and 50 μl of proteinase K solution (recombinant, PCR grade, 18 mg/ml; Roche Diagnostics) were combined and then incubated at 65°C for 10 min. Following incubation, lysate was transferred to a bead tube (bead tubes for the PureLink microbiome DNA purification kit; Invitrogen) for bead beating on the TissueLyser (Qiagen, Germany) at 50 Hz for 5 min, after which 500 μl of lysate was transferred for extraction using the MagNA Pure 96 DNA and viral nucleic acid (NA) large-volume kit (Roche Diagnostics) and the manufacturer’s Pathogen Universal 500 3.1 protocol with an elution volume of 100 μl for vaginal swabs and 50 μl for penile swabs. Reagent-only negative controls were extracted in parallel for the prelysis using PBS and using ultrapure water (Sigma-Aldrich) for the MagNA Pure 96 extraction.

On receipt, urine samples were immediately transferred to Royal Women’s Hospital, Melbourne, Australia, where they were centrifuged using a refrigerated Heraeus Megafuge 16R (Thermo Fisher Scientific, USA) at 5,580 relative centrifugal force for 30 min at 4°C. Supernatant was removed to within 2 ml of the pellet. The pellet was resuspended in the remaining supernatant and stored at −80°C until extraction. DNA was extracted from 1 ml of urine concentrate using the saliva and urine sample protocol for the PureLink microbiome DNA purification kit (Invitrogen) with an elution volume of 50 μl. A sample comprising the stabilization medium AssayAssure Genelock (Sierra Molecular, USA) and PBS was extracted for each kit as the reagent-only negative controls.

PCR amplification of the V3-V4 hypervariable regions of the 16S rRNA gene was performed by Micromon Genomics (Monash University, Victoria, Australia) using primers 341F/805R and dual indexing based on the 16S metagenomics protocol (Illumina, San Diego, CA, USA). Amplicons were sequenced on the MiSeq platform using v3 chemistry (600-cycle kit; Illumina) at Micromon Genomics. Reagent and PCR negative controls and positive controls (mock microbial community standards) were processed and sequenced alongside samples (Table S6). The microbial composition of negative and positive controls is shown in Fig. S3.

10.1128/mBio.02323-21.6 TABLE S6 List of controls processed alongside biological specimens. Download Table S6, PDF file, 0.4 MB. Copyright © 2021 Plummer et al. 2021 Plummer et al. https://creativecommons.org/licenses/by/4.0/ This content is distributed under the terms of the Creative Commons Attribution 4.0 International license.

10.1128/mBio.02323-21.10 FIG S3 Compositional profile of negative and positive controls included in the study. (A) Heatmap of the relative abundance of bacterial genera in all negative controls, ordered by control type (PCR negative controls, PBS, Ultrapure water, AssayAssure Genelock). (B) Stacked bar graphs showing the relative abundance of bacterial genera present in the four mock community control samples, as well as the theoretical composition of the mock communities (BEI_mock = BEI resources HM-276D genomic DNA from microbial mock community B; Zymo_mock = ZymoBIOMICS microbial community standard, catalog no. D6300). Download FIG S3, PDF file, 0.2 MB. Copyright © 2021 Plummer et al. 2021 Plummer et al. https://creativecommons.org/licenses/by/4.0/ This content is distributed under the terms of the Creative Commons Attribution 4.0 International license.

Adapter removal and demultiplexing were performed by Micromon Genomics. The 16S rRNA gene-amplifying primer sequences were removed from sequencing reads using Cutadapt v2.4 (71). DADA2 v1.16.0 (72) was used to quality filter and trim the sequencing reads, infer amplicon sequence variants (ASVs), merge paired reads, and remove chimeras. Taxonomic classification of ASVs was also performed using DADA2 and the DADA2-formatted SILVA database v138 (73). Species-level classification of *Lactobacillus* ASVs and classification of ASVs matching “*Candidatus* Lachnocurva vaginae” (formerly BVAB-1), BVAB-2, and Mageeibacillus indolicus (formerly BVAB-3) was performed as previously described (74).

ASVs identified as likely contaminants were removed. Contaminants included ASVs that were present (i) only in negative-control specimens or (ii) in higher prevalence and/or abundance in negative-control specimens compared to biological specimens and not expected in the biological context (Table S7). We additionally removed ASVs that were of nonbacterial origin, had a total abundance of <0.001%, or were present in only one specimen.

10.1128/mBio.02323-21.7 TABLE S7 ASVs identified as potential contaminants. Download Table S7, PDF file, 0.3 MB. Copyright © 2021 Plummer et al. 2021 Plummer et al. https://creativecommons.org/licenses/by/4.0/ This content is distributed under the terms of the Creative Commons Attribution 4.0 International license.

### Sequence analysis.

RStudio v1.3.959 (75) running R v4.0.3 (76) was employed for all analyses and for generating figures, unless stated otherwise.

NMDS and ANOSIM were used to visualize and test for global differences in the microbiota composition following partner treatment. We performed two analyses for each genital site comparing (i) day-0 versus day-8 specimens and (ii) day-0 versus endpoint specimens. NMDS and ANOSIM were performed with vegan (77) using the Bray-Curtis dissimilarity index and ASV data. NMDS plots were drawn using ggplot2 (78).

ASVs with identical taxonomy were merged for subsequent analyses. Stacked bar plots were drawn using ggplot2 (78).

ALDEX2 (13, 14) was used to identify taxa that were differentially abundant between pretreatment and posttreatment samples, using the sample comparisons as described above for each genital site: (i) day 0 versus day 8 and (ii) day 0 versus the endpoint. We generated 128 center log-ratio transformed Dirichlet Monte Carlo instances and tested for differentially abundant taxa using the Wilcoxon test, followed by a Benjamini-Hochberg FDR correction. Taxa with an FDR of <0.05 were considered significant. Boxplots were drawn using ggplot2 (78).

ANCOM (15) v2.1 was used to identify differences longitudinally in the abundance of taxa between couples who recurred and couples who were cured. All longitudinal specimens (i.e., those collected at weeks 4, 8, and 12) were included in the ANCOM analyses; specimens collected at day 0 and day 8 were excluded. The ANCOM framework accounts for the compositional nature of microbiota data by applying a pseudocount of 1 to all taxa and comparing the log-transformed ratio of the abundance of each pair of taxa between study groups. Specifically, we regressed the log-transformed ratio of the abundance of each pair of taxa against recurrence status, with participant as a random effect. A cutoff value of 0.7 was applied to identify taxa that were differentially abundant, meaning that the null hypothesis was rejected in ≥70% of comparisons, using an FDR of <0.05. Structural zeros were identified (79), and taxa that were present in ≤10% of samples were excluded.

The FastSpar (16) implementation of SparrCC (80) was used to examine the correlation between taxa in the female and male genital microbiota of sexual partners at day 0, day 8, and longitudinally (i.e., weeks 4, 8, and 12), taking into account the compositional nature of microbiota data. Taxa with an absolute correlation coefficient of >0.3 and *P *< 0.05 were considered significant.

### Data availability.

Raw sequence data are available in the NCBI Sequence Read Archive (BioProject identifier PRJNA735440).
