# Supplementary figures and images for: A Prospective, Open-Label Pilot Study of Concurrent Male Partner Treatment for Bacterial Vaginosis
Source: mBio. 2021 Oct 19;12(5):e02323-21. doi: 10.1128/mBio.02323-21 (PMC8524345; doi:10.1128/mBio.02323-21)

A

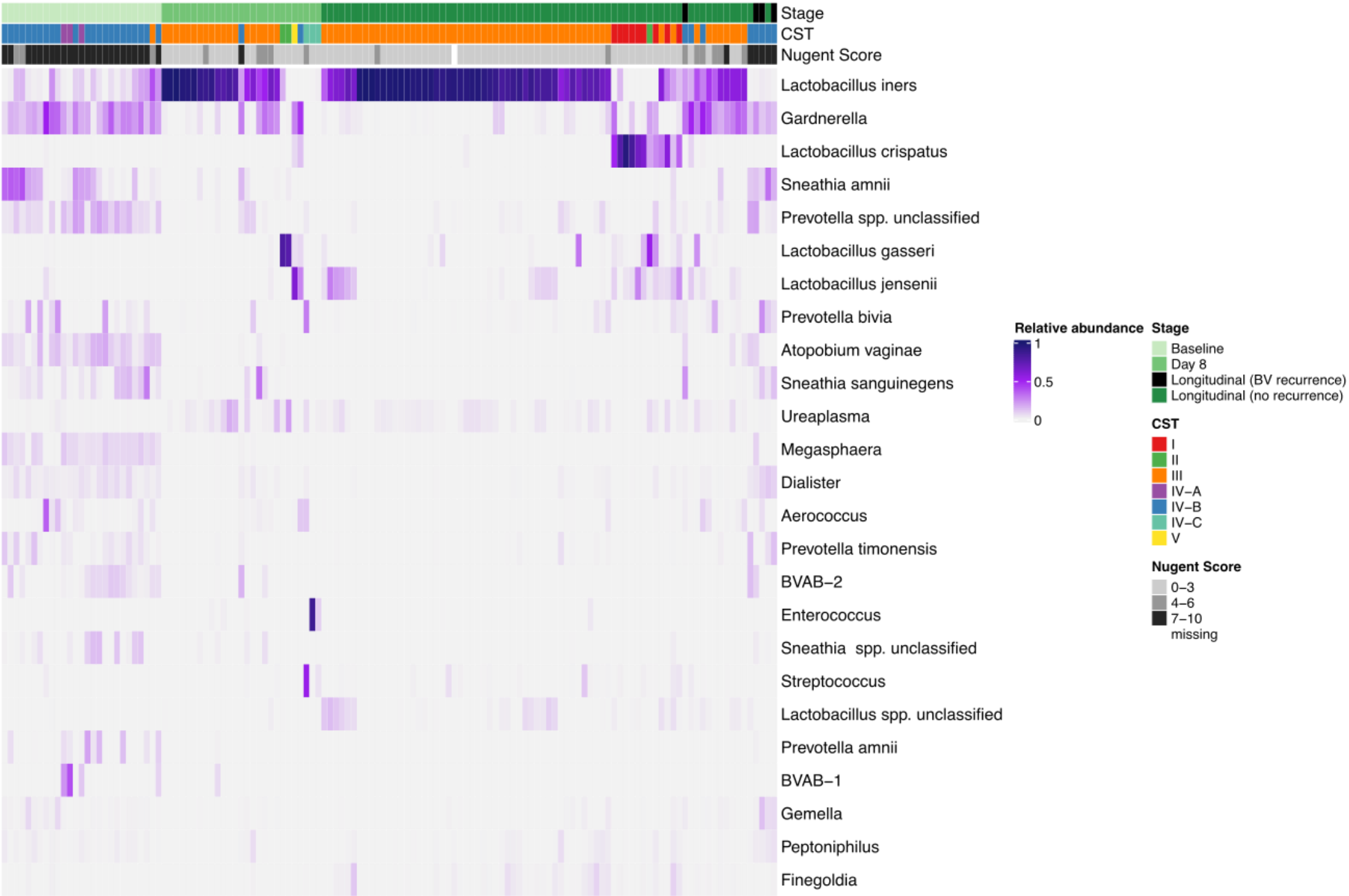

B

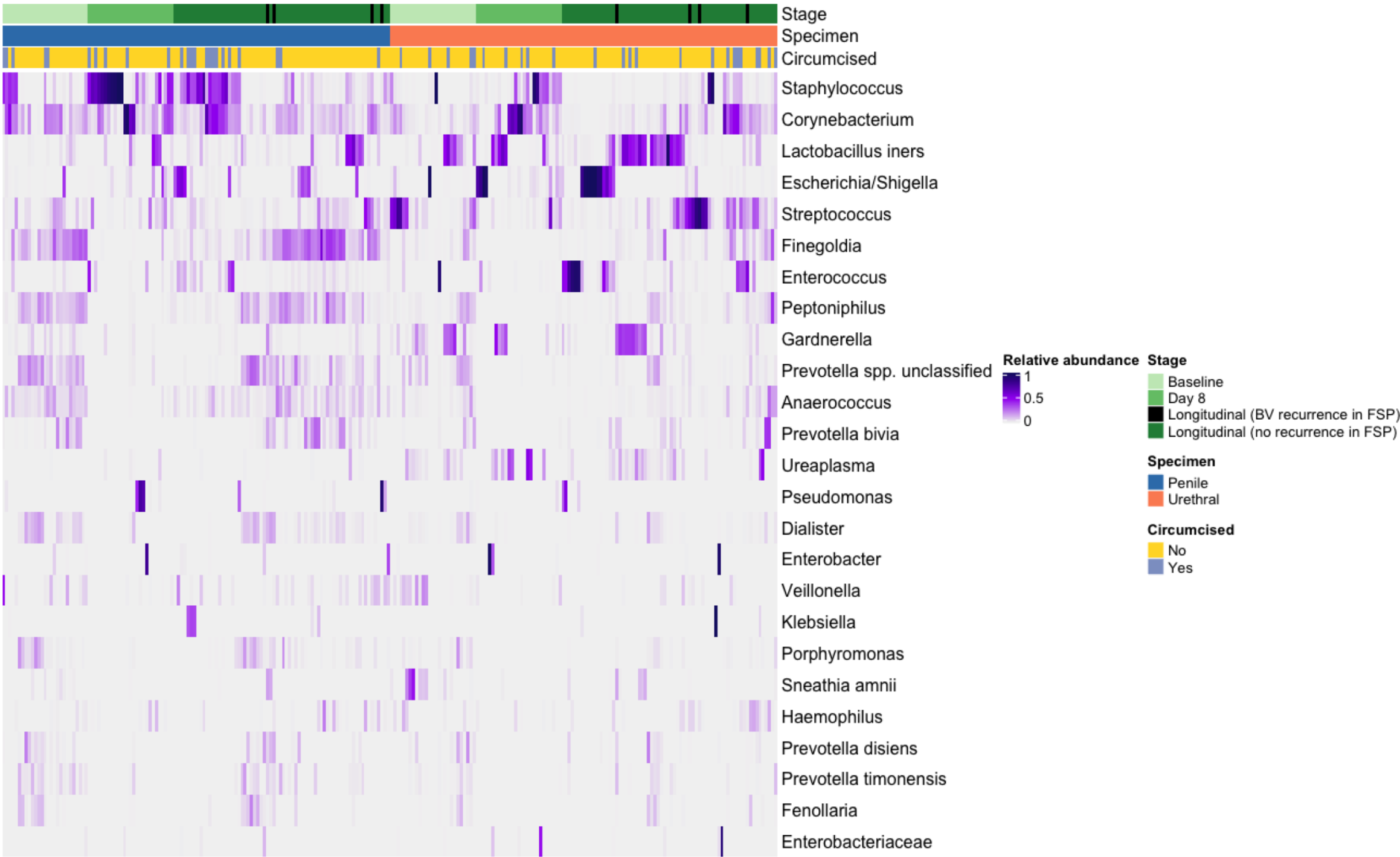

Supplement: FIG S1 [file mbio.02323-21-sf001.pdf]

**A**

## Vaginal specimens

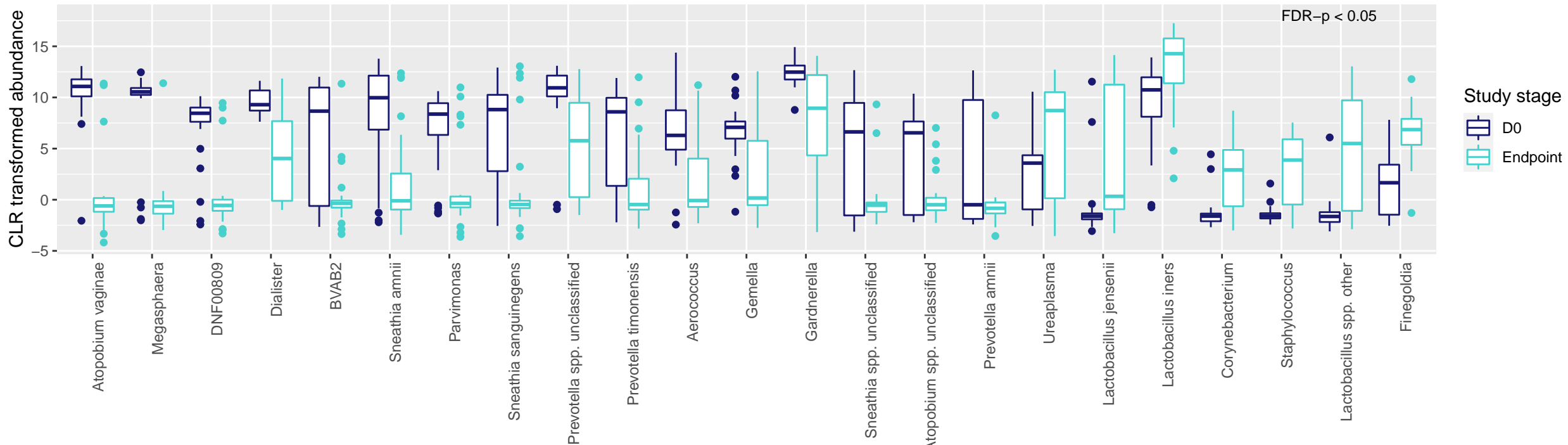**B**

## Cutaneous penile specimens

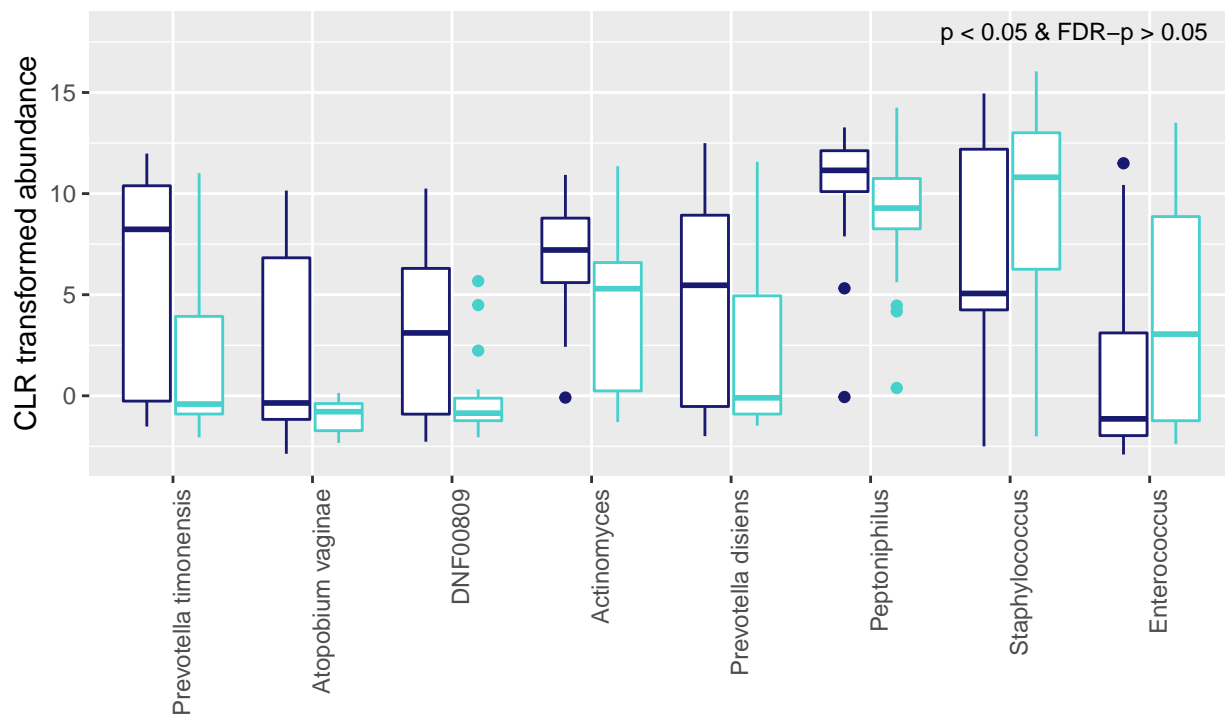**C**

## Urethral specimens

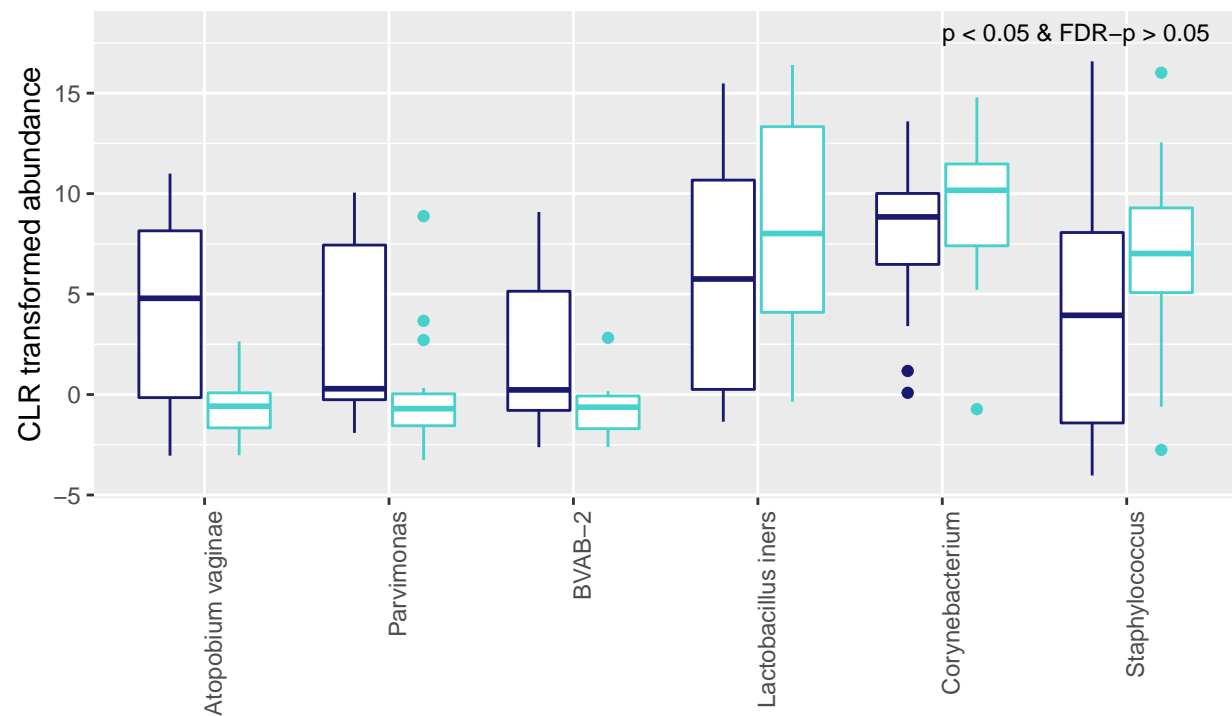

Supplement: FIG S2 [file mbio.02323-21-sf002.pdf]

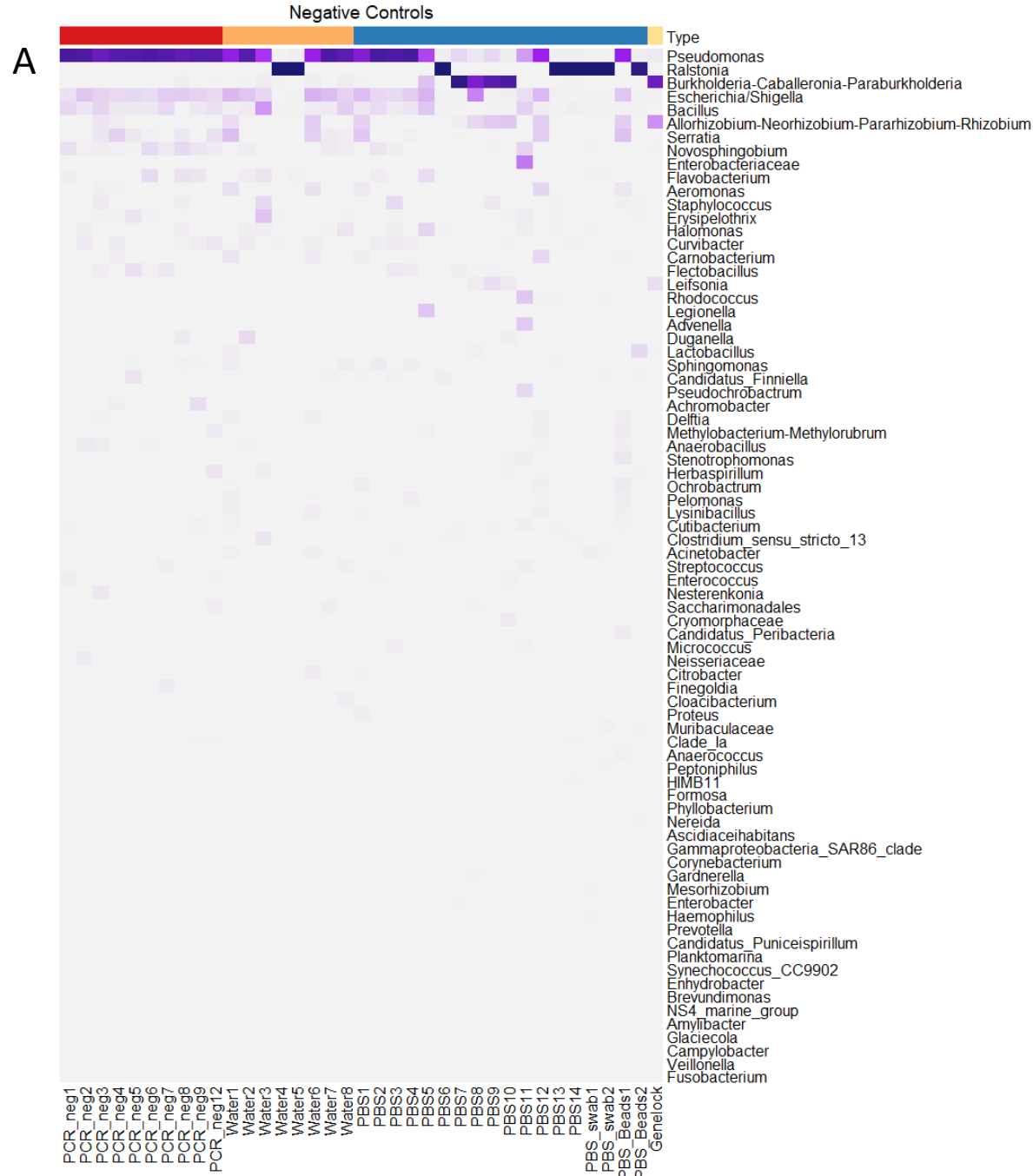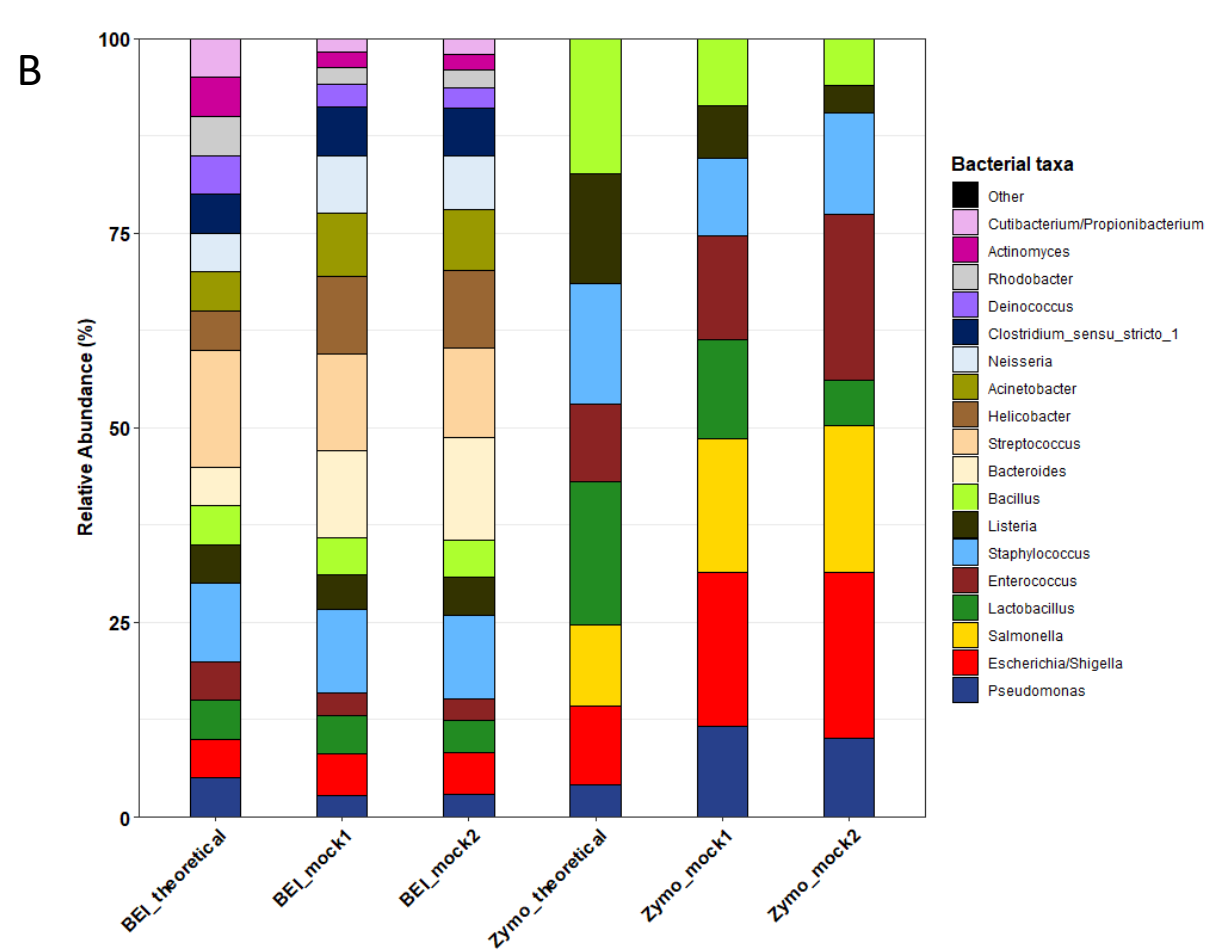

Supplement: FIG S3 [file mbio.02323-21-sf003.pdf]
