# Supplementary material for: A Prospective, Open-Label Pilot Study of Concurrent Male Partner Treatment for Bacterial Vaginosis
Source: mBio. 2021 Oct 19;12(5):e02323-21. doi: 10.1128/mBio.02323-21 (PMC8524345; doi:10.1128/mBio.02323-21)
Supplement: TABLE S1 [file mbio.02323-21-st001.pdf]

Table S1. Taxa that are differentially abundant in vaginal samples in day-0 *vs* day-8 specimens and day-0 vs endpoint specimens (ALDEX2 analysis)

Taxon[a]	Mean relative abundance at Day 0 (%)	Mean relative abundance at Day-8 (%)	Mean relative abundance at Endpoint (%)	Day 0 vs Day-8			Day-0 vs Endpoint		
				p-value[b]	FDR-p[c]	Effect size[d]	p-value[b]	FDR-p[c]	Effect size[d]
Atopobium vaginae#*	7.37	0.17	0.69	5.32E-07	**1.18E-05**	-1.59	2.68E-07	**1.23E-05**	-1.82
Megasphaera#*	5.33	0.05	0.27	8.19E-06	**7.13E-05**	-1.54	1.17E-06	**2.95E-05**	-1.82
DNF00809#*	1.17	0.07	0.14	6.98E-06	**7.63E-05**	-1.32	6.26E-06	**9.69E-05**	-1.30
Dialister#*	2.86	0.34	0.98	1.74E-07	**7.45E-06**	-1.25	1.56E-06	**4.18E-05**	-1.02
Parvimonas#*	1.50	0.06	0.41	4.88E-05	**3.33E-04**	-1.10	2.96E-04	**1.94E-03**	-0.89
Prevotella spp. unclassified#*	8.48	0.95	1.92	5.43E-06	**6.28E-05**	-1.09	7.18E-05	**6.62E-04**	-0.79
Gemella#*	0.89	0.07	0.82	2.85E-05	**2.24E-04**	-1.01	1.90E-04	**1.39E-03**	-0.72
*Peptoniphilus**	0.68	0.22	0.72	6.77E-05	**4.69E-04**	-1.01	2.24E-01	3.85E-01	-0.24
*Anaerococcus**	0.40	0.00	0.57	7.37E-04	**2.84E-03**	-0.86	3.12E-01	4.69E-01	0.13
Sneathia amnii#*	10.74	0.89	1.49	2.07E-04	**1.08E-03**	-0.81	6.87E-05	**5.84E-04**	-0.90
Prevotella timonensis#*	2.44	0.09	0.63	1.59E-03	**5.80E-03**	-0.80	4.79E-04	**2.91E-03**	-0.79
BVAB-2#*	3.09	0.68	0.27	1.59E-03	**5.73E-03**	-0.77	2.42E-04	**1.48E-03**	-0.92
*Porphyromonas**	1.24	0.05	0.51	3.37E-03	**9.94E-03**	-0.66	2.20E-01	3.58E-01	-0.27
Gardnerella#*	20.32	7.91	7.79	3.28E-04	**1.85E-03**	-0.64	3.70E-05	**4.35E-04**	-0.67
Sneathia sanguinegens#*	4.36	1.56	1.72	1.96E-03	**7.12E-03**	-0.62	8.60E-04	**4.75E-03**	-0.80
*Prevotella disiens**	0.82	0.04	0.69	4.49E-03	**1.26E-02**	-0.61	3.44E-01	4.99E-01	-0.22
Atopobium spp. unclassified#*	0.54	0.04	0.04	1.24E-02	**2.89E-02**	-0.57	1.39E-02	**4.38E-02**	-0.64
Sneathia spp. unclassified#	2.79	0.33	0.12	3.86E-02	7.47E-02	-0.45	2.92E-03	**1.26E-02**	-0.66
Aerococcus#*	2.53	0.93	0.41	1.26E-02	**3.31E-02**	-0.45	1.72E-04	**1.23E-03**	-0.77
Mageeibacillus indolicus (BVAB-3)	0.28	0.00	0.00	1.26E-01	1.84E-01	-0.34	3.56E-02	8.90E-02	-0.46
Prevotella amnii#	2.71	0.02	0.03	6.00E-02	1.05E-01	-0.32	1.61E-02	**5.00E-02**	-0.43
Finegoldia#	0.09	0.04	1.02	5.71E-01	6.31E-01	-0.09	3.06E-06	**5.64E-05**	1.11
Lactobacillus jensenii#	0.21	3.29	4.09	9.61E-02	1.38E-01	0.21	4.87E-03	**1.80E-02**	0.58
Lactobacillus gasseri	0.02	6.26	2.34	4.86E-02	7.87E-02	0.26	6.73E-02	1.37E-01	0.28
*Haemophilus**	0.00	0.13	0.04	2.41E-02	**4.94E-02**	0.43	1.50E-01	2.48E-01	0.32
*Enterococcus**	0.00	3.59	0.00	1.64E-02	**3.48E-02**	0.45	5.28E-01	6.33E-01	0.06
*Streptococcus**	0.06	2.13	0.20	1.23E-02	**2.89E-02**	0.55	1.89E-02	5.16E-02	0.55
Lactobacillus iners#*	8.41	57.20	49.25	3.48E-04	**1.93E-03**	0.71	2.42E-04	**1.73E-03**	0.59
*Escherichia/Shigella**	0.00	1.06	0.01	8.32E-04	**3.21E-03**	0.75	1.95E-01	2.97E-01	0.28
Lactobacillus spp. unclassified#*	0.01	0.07	2.13	1.48E-03	**3.99E-03**	0.79	9.75E-05	**7.07E-04**	0.99
Staphylococcus#*	0.00	0.40	0.08	3.97E-04	**1.75E-03**	0.86	4.93E-04	**2.79E-03**	0.92
Ureaplasma#*	0.19	3.78	1.35	1.33E-05	**1.23E-04**	0.92	8.25E-03	**2.84E-02**	0.54
Corynebacterium#*	0.00	0.72	0.14	2.36E-05	**1.91E-04**	1.11	4.22E-03	**1.51E-02**	0.68

[a]Only those taxa with a p-value<0.05 for either the Day-0 *vs* Day-8 or Day-0 *vs* Endpoint comparison are shown in this table. * FDR-p<0.05 for Day-0 *vs* Day-8 comparison; # FDR-p<0.05 for Day-0 vs Endpoint comparison

[b]Expected P value of Wilcoxon rank test

[c]Expected Benjamini-Hochberg corrected P value of Wilcoxon test. Bold indicates statistically significant difference at FDR-p<0.05

[d]Median difference in CLR transformed abundance between Day-0 vs Day-8 groups /median of the largest difference in CLR transformed abundance within Day-0 and Day-8 groups

[e]Expected P value of Wilcoxon rank test

[f]Expected Benjamini-Hochberg corrected P value of Wilcoxon test. Bold indicates statistically significant difference at FDR-p<0.05

[g]Median difference in CLR transformed abundance between Day-0 vs Endpoint groups /median of the largest difference in CLR transformed abundance within Day-0 and Endpoint groups
