# Supplementary material for: A Prospective, Open-Label Pilot Study of Concurrent Male Partner Treatment for Bacterial Vaginosis
Source: mBio. 2021 Oct 19;12(5):e02323-21. doi: 10.1128/mBio.02323-21 (PMC8524345; doi:10.1128/mBio.02323-21)
Supplement: TABLE S2 [file mbio.02323-21-st002.pdf]

Table S2. Taxa that are differentially abundant in cutaneous penile samples in day-0 *vs* day-8 specimens and day-0 vs endpoint specimens (ALDEX2 analysis)

Taxon[a]	Mean relative abundance at Day 0 (%)	Mean relative abundance at Day-8 (%)	Mean relative abundance at Endpoint (%)	Day 0 vs Day-8			Day-0 vs Endpoint		
				**p-value[b]**	**FDR-p[c]**	**Effect size[d]**	**p-value[e]**	**FDR-p[f]**	**Effect size[g]**
*Anaerococcus**	5.53	0.15	3.70	1.49E-08	**2.02E-06**	-2.32	8.41E-02	4.04E-01	-0.23
*Peptoniphilus**	8.42	0.17	5.38	4.00E-07	**1.92E-05**	-1.65	1.60E-02	1.89E-01	-0.35
*Finegoldia**	13.39	0.45	11.83	1.42E-06	**5.38E-05**	-1.66	4.35E-01	7.65E-01	-0.12
Prevotella spp. unclassified*	8.01	0.20	3.53	1.18E-05	**2.95E-04**	-1.20	5.40E-02	3.26E-01	-0.26
*Dialister**	4.01	0.38	1.99	1.87E-04	**2.09E-03**	-0.91	1.09E-01	4.24E-01	-0.27
*Staphylococcus**	8.86	41.43	12.89	2.19E-04	**3.36E-03**	0.84	1.27E-02	1.83E-01	0.34
*Prevotella timonensis**	2.34	0.23	0.91	4.13E-04	**3.96E-03**	-0.86	4.37E-03	1.07E-01	-0.64
*Porphyromonas**	2.97	0.03	1.46	4.25E-04	**4.19E-03**	-0.81	3.41E-01	6.45E-01	-0.15
*Campylobacter**	1.26	0.01	0.92	1.11E-03	**8.27E-03**	-0.77	3.78E-01	7.01E-01	-0.19
*Actinomyces**	0.91	0.32	0.48	8.33E-04	**8.69E-03**	-0.77	1.03E-02	1.69E-01	-0.41
*Prevotella disiens**	1.87	0.02	1.33	2.85E-03	**1.67E-02**	-0.72	2.56E-02	2.16E-01	-0.40
*Peptostreptococcus**	0.64	0.00	0.38	1.44E-02	**4.96E-02**	-0.59	1.75E-01	5.07E-01	-0.25
Negativicoccus	0.89	0.00	0.13	1.34E-02	5.02E-02	-0.54	6.11E-02	3.19E-01	-0.42
Escherichia/Shigella	1.47	3.05	5.82	1.31E-02	5.30E-02	0.49	7.79E-01	9.17E-01	0.01
DNF00809	0.24	0.00	0.01	1.71E-02	5.51E-02	-0.58	1.18E-02	1.50E-01	-0.58
Prevotella bivia	3.28	0.00	1.99	1.56E-02	6.12E-02	-0.56	7.25E-01	8.95E-01	-0.02
Howardella	0.08	0.00	0.03	3.43E-02	8.68E-02	-0.53	5.64E-02	2.86E-01	-0.35
Varibaculum	0.08	0.00	0.09	3.64E-02	9.33E-02	-0.48	1.25E-01	4.02E-01	-0.29
Pseudomonas	0.07	7.01	3.95	3.71E-02	9.62E-02	0.36	1.65E-01	4.36E-01	0.23
Fenollaria	1.85	0.02	0.72	3.99E-02	1.02E-01	-0.38	4.22E-01	7.03E-01	-0.11
Atopobium vaginae	0.61	0.02	0.00	9.03E-02	1.85E-01	-0.40	9.62E-03	1.33E-01	-0.60
Enterococcus	0.54	2.65	2.17	1.06E-01	1.96E-01	0.25	4.89E-02	2.86E-01	0.35

[a]Only those taxa with a p-value <0.05 for either the Day-0 *vs* Day-8 or Day-0 *vs* Endpoint comparison are shown in this table. * FDR-p<0.05 for Day-0 *vs* Day-8 comparison

[b]Expected P value of Wilcoxon rank test

[c]Expected Benjamini-Hochberg corrected P value of Wilcoxon test. Bold indicates statistically significant difference at FDR-p<0.05

[d]Median difference in CLR transformed abundance between Day-0 vs Day-8 groups /median of the largest difference in CLR transformed abundance within Day-0 and Day-8 groups

[e]Expected P value of Wilcoxon rank test

[f]Expected Benjamini-Hochberg corrected P value of Wilcoxon test. Bold indicates statistically significant difference at FDR-p<0.05

[g]Median difference in CLR transformed abundance between Day-0 vs Endpoint groups /median of the largest difference in CLR transformed abundance within Day-0 and
