# Supplementary material for: A Prospective, Open-Label Pilot Study of Concurrent Male Partner Treatment for Bacterial Vaginosis
Source: mBio. 2021 Oct 19;12(5):e02323-21. doi: 10.1128/mBio.02323-21 (PMC8524345; doi:10.1128/mBio.02323-21)
Supplement: TABLE S3 [file mbio.02323-21-st003.pdf]

Table S3. Taxa that are differentially abundant in urethral samples in day-0 *vs* day-8 specimens and day-0 vs endpoint specimens (ALDEX2 analysis)

Taxon[a]	Mean relative abundance at Day 0 (%)	Mean relative abundance at Day-8 (%)	Mean relative abundance at Endpoint (%)	Day 0 vs Day-8			Day-0 vs Endpoint		
				p-value[b]	FDR-p[c]	Effect size[d]	p-value[e]	FDR-p[f]	Effect size[g]
*Anaerococcus**	1.47	0.00	2.88	1.49E-04	**2.82E-03**	-1.05	3.15E-01	7.00E-01	0.21
*Finegoldia**	1.86	0.04	4.55	1.60E-04	**3.19E-03**	-1.00	2.09E-01	6.32E-01	0.31
*Peptoniphilus**	2.42	0.04	3.62	2.41E-04	**4.36E-03**	-1.00	6.40E-01	8.72E-01	0.11
Prevotella spp. unclassified*	3.89	0.04	1.33	9.12E-04	**1.10E-02**	-0.79	1.16E-01	4.96E-01	-0.25
*Dialister**	1.31	0.03	0.67	1.17E-03	**1.21E-02**	-0.79	1.64E-01	5.42E-01	-0.18
*Escherichia/Shigella**	4.10	14.87	3.79	3.89E-03	**2.90E-02**	0.50	6.03E-01	8.59E-01	0.08
*Staphylococcus**	4.77	15.28	4.60	1.64E-03	**1.81E-02**	0.65	1.76E-03	7.92E-02	0.37
Porphyromonas	1.22	0.00	0.27	1.76E-02	6.41E-02	-0.55	4.09E-01	7.34E-01	-0.12
Campylobacter	0.41	0.00	0.28	1.86E-02	6.64E-02	-0.55	5.44E-01	8.11E-01	-0.07
Rothia	0.22	0.31	0.01	2.55E-02	7.49E-02	0.44	6.21E-01	8.49E-01	0.08
Atopobium vaginae	0.65	0.17	0.00	2.28E-02	7.67E-02	-0.54	2.68E-03	6.98E-02	-0.69
Streptococcus	15.86	3.94	17.70	3.35E-02	1.11E-01	-0.32	5.34E-01	8.34E-01	0.10
Veillonella	3.23	0.39	0.51	3.22E-02	9.91E-02	-0.46	1.22E-01	5.14E-01	-0.33
Gardnerella	7.51	4.68	6.02	1.86E-02	7.30E-02	-0.46	4.81E-01	7.99E-01	-0.16
Prevotella disiens	1.75	0.00	0.50	2.15E-02	7.22E-02	-0.51	3.48E-01	6.86E-01	-0.13
Prevotella timonensis	0.59	0.01	0.76	1.35E-02	5.30E-02	-0.61	3.05E-01	6.65E-01	-0.19
Sneathia amnii	4.90	0.03	0.83	3.48E-02	1.02E-01	-0.41	1.42E-01	5.15E-01	-0.34
Sneathia sanguinegens	4.54	0.00	0.31	2.35E-02	7.81E-02	-0.45	7.00E-02	3.90E-01	-0.35
BVAB-2	0.32	0.00	0.00	1.40E-01	2.42E-01	-0.28	3.99E-02	2.70E-01	-0.42
Lactobacillus iners	8.96	10.78	18.02	1.93E-01	3.31E-01	0.13	3.21E-02	3.06E-01	0.25
Corynebacterium	3.69	21.37	7.59	2.80E-01	4.31E-01	0.31	3.25E-02	3.26E-01	0.26
Parvimonas	0.95	0.06	0.06	5.91E-02	1.41E-01	-0.43	3.56E-02	2.82E-01	-0.46

[a]Only those taxa with a p-value <0.05 for either the Day-0 *vs* Day-8 or Day-0 *vs* Endpoint comparison are shown in this table. * FDR-p<0.05 for Day-0 *vs* Day-8 comparison

[b]Expected P value of Wilcoxon rank test

[c]Expected Benjamini-Hochberg corrected P value of Wilcoxon test. Bold indicates statistically significant difference at FDR-p<0.05

[d]Median difference in CLR transformed abundance between Day-0 vs Day-8 groups /median of the largest difference in CLR transformed abundance within Day-0 and Day-8 groups

[e]Expected P value of Wilcoxon rank test

[f]Expected Benjamini-Hochberg corrected P value of Wilcoxon test. Bold indicates statistically significant difference at FDR-p<0.05

[g]Median difference in CLR transformed abundance between Day-0 vs Endpoint groups /median of the largest difference in CLR transformed abundance within Day-0 and Endpoint groups
