# Supplementary material for: A Prospective, Open-Label Pilot Study of Concurrent Male Partner Treatment for Bacterial Vaginosis
Source: mBio. 2021 Oct 19;12(5):e02323-21. doi: 10.1128/mBio.02323-21 (PMC8524345; doi:10.1128/mBio.02323-21)
Supplement: TABLE S4 [file mbio.02323-21-st004.pdf]

Table S4. Analysis of Composition of Microbiomes (ANCOM) of longitudinal vaginal, cutaneous penile and urethral specimens from couples that were cured *vs* couples who recurred[a]

	Taxon	CLR-mean difference[b]	W-statistic[c]	Detected_0.9[d]	Detected_0.8[e]	Detected_0.7[f]	Detected_0.6[g]
Vaginal microbiota	***Atopobium***	**0.14**	**Inf**	**TRUE**	**TRUE**	**TRUE**	**TRUE**
	Ezakiella	**0.24**	**Inf**	**TRUE**	**TRUE**	**TRUE**	**TRUE**
	Flavobacterium	**0.17**	**Inf**	**TRUE**	**TRUE**	**TRUE**	**TRUE**
	Fusobacterium	**0.00**	**Inf**	**TRUE**	**TRUE**	**TRUE**	**TRUE**
	Murdochiella	**0.20**	**Inf**	**TRUE**	**TRUE**	**TRUE**	**TRUE**
	Rothia	**0.15**	**Inf**	**TRUE**	**TRUE**	**TRUE**	**TRUE**
	Lactobacillus antri	**-0.09**	**Inf**	**TRUE**	**TRUE**	**TRUE**	**TRUE**
	Lactobacillus coleohominis	**-0.07**	**Inf**	**TRUE**	**TRUE**	**TRUE**	**TRUE**
	Lactobacillus crispatus	**-0.76**	**Inf**	**TRUE**	**TRUE**	**TRUE**	**TRUE**
	Lactobacillus gasseri	**-0.45**	**Inf**	**TRUE**	**TRUE**	**TRUE**	**TRUE**
	Sneathia sanguinegens	**1.08**	**40**	**TRUE**	**TRUE**	**TRUE**	**TRUE**
	Corynebacterium	**0.31**	**33**	FALSE	FALSE	**TRUE**	**TRUE**
	Dialister	**0.52**	**31**	FALSE	FALSE	**TRUE**	**TRUE**
	Gemella	**0.84**	**31**	FALSE	FALSE	**TRUE**	**TRUE**
	Sneathia amnii	**1.20**	**31**	FALSE	FALSE	**TRUE**	**TRUE**
	Parvimonas	0.12	30	FALSE	FALSE	FALSE	TRUE
	Peptostreptococcus	0.02	28	FALSE	FALSE	FALSE	TRUE
	Atopobium vaginae	0.29	23	FALSE	FALSE	FALSE	FALSE
	Muribaculaceae	0.23	12	FALSE	FALSE	FALSE	FALSE
	Actinomyces	-0.84	7	FALSE	FALSE	FALSE	FALSE
	Aerococcus	0.34	2	FALSE	FALSE	FALSE	FALSE
	Alloprevotella	-0.07	6	FALSE	FALSE	FALSE	FALSE
	Anaerococcus	-0.25	1	FALSE	FALSE	FALSE	FALSE
	Bifidobacterium	-0.71	7	FALSE	FALSE	FALSE	FALSE
	Campylobacter	-0.33	12	FALSE	FALSE	FALSE	FALSE
	Enterococcus	-0.47	7	FALSE	FALSE	FALSE	FALSE
	Escherichia/Shigella	0.04	1	FALSE	FALSE	FALSE	FALSE
	Fenollaria	-0.64	13	FALSE	FALSE	FALSE	FALSE
	Finegoldia	-1.00	15	FALSE	FALSE	FALSE	FALSE
	Granulicatella	-0.11	8	FALSE	FALSE	FALSE	FALSE
	Haemophilus	0.48	7	FALSE	FALSE	FALSE	FALSE
	Howardella	-0.47	14	FALSE	FALSE	FALSE	FALSE
	Lactobacillus	0.04	25	FALSE	FALSE	FALSE	FALSE
	Lawsonella	0.21	8	FALSE	FALSE	FALSE	FALSE
	Mobiluncus	-0.07	7	FALSE	FALSE	FALSE	FALSE
	Mycoplasma	0.52	3	FALSE	FALSE	FALSE	FALSE
	Neisseria	-0.18	6	FALSE	FALSE	FALSE	FALSE
	Peptococcus	-0.47	6	FALSE	FALSE	FALSE	FALSE
	Peptoniphilus	0.25	3	FALSE	FALSE	FALSE	FALSE
	Porphyromonas	-0.12	6	FALSE	FALSE	FALSE	FALSE
	Prevotella spp. unclassified	0.13	7	FALSE	FALSE	FALSE	FALSE
	Solobacterium	-0.32	11	FALSE	FALSE	FALSE	FALSE
	Staphylococcus	0.78	16	FALSE	FALSE	FALSE	FALSE
	Streptococcus	-0.15	7	FALSE	FALSE	FALSE	FALSE
	Sutterella	0.34	12	FALSE	FALSE	FALSE	FALSE
	Ureaplasma	-1.61	1	FALSE	FALSE	FALSE	FALSE
	Veillonella	0.28	8	FALSE	FALSE	FALSE	FALSE
	Gardnerella	1.73	0	FALSE	FALSE	FALSE	FALSE
	Lactobacillus iners	-2.70	1	FALSE	FALSE	FALSE	FALSE
	Lactobacillus jensenii	-0.95	4	FALSE	FALSE	FALSE	FALSE
	Prevotella bivia	1.05	18	FALSE	FALSE	FALSE	FALSE
	Prevotella disiens	0.68	8	FALSE	FALSE	FALSE	FALSE
	Prevotella melaninogenica	0.05	8	FALSE	FALSE	FALSE	FALSE
	Prevotella timonensis	0.41	1	FALSE	FALSE	FALSE	FALSE
	Alloscardovia	**0.09**	**Inf**	**TRUE**	**TRUE**	**TRUE**	**TRUE**
	Arcanobacterium	**0.22**	**Inf**	**TRUE**	**TRUE**	**TRUE**	**TRUE**
	Ezakiella	**0.16**	**Inf**	**TRUE**	**TRUE**	**TRUE**	**TRUE**
	Fusobacterium	**0.25**	**Inf**	**TRUE**	**TRUE**	**TRUE**	**TRUE**
	Mycoplasma	**-0.17**	**Inf**	**TRUE**	**TRUE**	**TRUE**	**TRUE**
	Pseudomonas	**-0.05**	**Inf**	**TRUE**	**TRUE**	**TRUE**	**TRUE**
	Lactobacillus gasseri	**0.26**	**Inf**	**TRUE**	**TRUE**	**TRUE**	**TRUE**
	Streptococcus	1.22	0	FALSE	FALSE	FALSE	FALSE

Table S4. Analysis of Composition of Microbiomes (ANCOM) of longitudinal vaginal, cutaneous penile and urethral specimens from couples that were cured *vs* couples who recurred[a]

**Cutaneous penile microbiota**	*Enterococcus*	1.39	0	FALSE	FALSE	FALSE	FALSE
	Gardnerella	1.12	0	FALSE	FALSE	FALSE	FALSE
	Corynebacterium	0.19	0	FALSE	FALSE	FALSE	FALSE
	Gemella	1.22	0	FALSE	FALSE	FALSE	FALSE
	Sneathia amnii	1.07	0	FALSE	FALSE	FALSE	FALSE
	Prevotella bivia	0.64	0	FALSE	FALSE	FALSE	FALSE
	Prevotella timonensis	0.76	0	FALSE	FALSE	FALSE	FALSE
	Sneathia sanguinegens	0.67	0	FALSE	FALSE	FALSE	FALSE
	Lactobacillus iners	-0.63	0	FALSE	FALSE	FALSE	FALSE
	Prevotella disiens	0.51	1	FALSE	FALSE	FALSE	FALSE
	Peptoniphilus	0.14	0	FALSE	FALSE	FALSE	FALSE
	Anaerococcus	0.42	0	FALSE	FALSE	FALSE	FALSE
	Granulicatella	0.70	0	FALSE	FALSE	FALSE	FALSE
	Ureaplasma	-0.27	0	FALSE	FALSE	FALSE	FALSE
	Prevotella spp. unclassified	0.09	0	FALSE	FALSE	FALSE	FALSE
	Facklamia	0.27	0	FALSE	FALSE	FALSE	FALSE
	Finegoldia	-0.03	0	FALSE	FALSE	FALSE	FALSE
	Aerococcus	-0.31	0	FALSE	FALSE	FALSE	FALSE
	Haemophilus	-0.21	0	FALSE	FALSE	FALSE	FALSE
	Dialister	0.12	0	FALSE	FALSE	FALSE	FALSE
	Dermabacter	0.14	0	FALSE	FALSE	FALSE	FALSE
	Howardella	0.15	0	FALSE	FALSE	FALSE	FALSE
	Actinotignum	-0.24	0	FALSE	FALSE	FALSE	FALSE
	Prevotella melaninogenica	-0.11	0	FALSE	FALSE	FALSE	FALSE
	Atopobium deltae	-0.14	0	FALSE	FALSE	FALSE	FALSE
	Negativicoccus	-0.06	0	FALSE	FALSE	FALSE	FALSE
	Peptococcus	-0.04	0	FALSE	FALSE	FALSE	FALSE
	Peptostreptococcus	-0.19	0	FALSE	FALSE	FALSE	FALSE
	Veillonella	-0.22	0	FALSE	FALSE	FALSE	FALSE
	Rothia	-0.34	0	FALSE	FALSE	FALSE	FALSE
	Porphyromonas	-0.52	0	FALSE	FALSE	FALSE	FALSE
	Micrococcus	-0.37	0	FALSE	FALSE	FALSE	FALSE
	Escherichia/Shigella	-0.56	0	FALSE	FALSE	FALSE	FALSE
	Varibaculum	-0.47	1	FALSE	FALSE	FALSE	FALSE
	Murdochiella	-0.45	0	FALSE	FALSE	FALSE	FALSE
	Actinomyces	-0.35	0	FALSE	FALSE	FALSE	FALSE
	Fenollaria	-0.68	0	FALSE	FALSE	FALSE	FALSE
	Prevotellaceae	-0.69	0	FALSE	FALSE	FALSE	FALSE
	Staphylococcus	-0.46	0	FALSE	FALSE	FALSE	FALSE
	Campylobacter	-0.50	0	FALSE	FALSE	FALSE	FALSE
	Propionimicrobium	-0.70	0	FALSE	FALSE	FALSE	FALSE
	Mobiluncus	-0.77	0	FALSE	FALSE	FALSE	FALSE
	Sutterella	-0.60	0	FALSE	FALSE	FALSE	FALSE
	Lawsonella	-1.64	0	FALSE	FALSE	FALSE	FALSE
	Atopobium parvulum	**0.26**	**Inf**	**TRUE**	**TRUE**	**TRUE**	**TRUE**
	Coriobacteriales Inc Se	**0.43**	**Inf**	**TRUE**	**TRUE**	**TRUE**	**TRUE**
	Acinetobacter	**-0.06**	**Inf**	**TRUE**	**TRUE**	**TRUE**	**TRUE**
	Cutibacterium	**0.31**	**Inf**	**TRUE**	**TRUE**	**TRUE**	**TRUE**
	Enterobacter	**0.02**	**Inf**	**TRUE**	**TRUE**	**TRUE**	**TRUE**
	Micrococcus	**0.00**	**Inf**	**TRUE**	**TRUE**	**TRUE**	**TRUE**
	Murdochiella	**0.17**	**Inf**	**TRUE**	**TRUE**	**TRUE**	**TRUE**
	Pseudoclavibacter	**0.17**	**Inf**	**TRUE**	**TRUE**	**TRUE**	**TRUE**
	Ruminococcaceae UCG014	**0.21**	**Inf**	**TRUE**	**TRUE**	**TRUE**	**TRUE**
	Lactobacillus crispatus	**0.11**	**Inf**	**TRUE**	**TRUE**	**TRUE**	**TRUE**
	Lactobacillus gasseri	**0.13**	**Inf**	**TRUE**	**TRUE**	**TRUE**	**TRUE**
	Lactobacillus jensenii	**0.13**	**Inf**	**TRUE**	**TRUE**	**TRUE**	**TRUE**
	Prevotella buccalis	**0.07**	**Inf**	**TRUE**	**TRUE**	**TRUE**	**TRUE**
	Finegoldia	0.61	0	FALSE	FALSE	FALSE	FALSE
	Prevotella bivia	1.53	0	FALSE	FALSE	FALSE	FALSE
	Prevotella disiens	1.68	0	FALSE	FALSE	FALSE	FALSE
	Anaerococcus	0.68	0	FALSE	FALSE	FALSE	FALSE
	Peptoniphilus	0.38	0	FALSE	FALSE	FALSE	FALSE
	Prevotella spp. unclassified	0.57	0	FALSE	FALSE	FALSE	FALSE
	Dialister	1.25	0	FALSE	FALSE	FALSE	FALSE
	Prevotella timonensis	0.89	0	FALSE	FALSE	FALSE	FALSE

Table S4. Analysis of Composition of Microbiomes (ANCOM) of longitudinal vaginal, cutaneous penile and urethral specimens from couples that were cured *vs* couples who recurred[a]

**Urethral microbiota**	*Corynebacterium*	-0.99	0	FALSE	FALSE	FALSE	FALSE
	Pseudomonas	1.08	0	FALSE	FALSE	FALSE	FALSE
	Gemella	1.46	0	FALSE	FALSE	FALSE	FALSE
	Porphyromonas	0.53	0	FALSE	FALSE	FALSE	FALSE
	Fenollaria	0.37	0	FALSE	FALSE	FALSE	FALSE
	Veillonella	0.00	0	FALSE	FALSE	FALSE	FALSE
	Neisseria	0.28	0	FALSE	FALSE	FALSE	FALSE
	Haemophilus	0.30	0	FALSE	FALSE	FALSE	FALSE
	Gardnerella	0.56	0	FALSE	FALSE	FALSE	FALSE
	Aerococcus	0.21	0	FALSE	FALSE	FALSE	FALSE
	Prevotella melaninogenica	0.37	0	FALSE	FALSE	FALSE	FALSE
	Peptostreptococcus	0.28	0	FALSE	FALSE	FALSE	FALSE
	Actinomyces	0.46	0	FALSE	FALSE	FALSE	FALSE
	Prevotellaceae	-0.10	0	FALSE	FALSE	FALSE	FALSE
	Rothia	-0.04	0	FALSE	FALSE	FALSE	FALSE
	Sutterella	0.26	0	FALSE	FALSE	FALSE	FALSE
	Campylobacter	-0.15	0	FALSE	FALSE	FALSE	FALSE
	Granulicatella	0.15	0	FALSE	FALSE	FALSE	FALSE
	Fusobacterium	0.27	0	FALSE	FALSE	FALSE	FALSE
	Prevotella nanceiensis	0.03	0	FALSE	FALSE	FALSE	FALSE
	Negativicoccus	0.10	0	FALSE	FALSE	FALSE	FALSE
	Muribaculaceae	-0.07	0	FALSE	FALSE	FALSE	FALSE
	Staphylococcus	-1.94	0	FALSE	FALSE	FALSE	FALSE
	Prevotella corporis	-0.05	0	FALSE	FALSE	FALSE	FALSE
	Atopobium deltae	-0.17	1	FALSE	FALSE	FALSE	FALSE
	Mobiluncus	-0.44	0	FALSE	FALSE	FALSE	FALSE
	Streptococcus	-1.10	0	FALSE	FALSE	FALSE	FALSE
	Dermabacter	0.04	0	FALSE	FALSE	FALSE	FALSE
	Peptococcus	-0.22	0	FALSE	FALSE	FALSE	FALSE
	Enterococcus	-0.96	0	FALSE	FALSE	FALSE	FALSE
	Bergeyella	-0.30	0	FALSE	FALSE	FALSE	FALSE
	Howardella	0.03	0	FALSE	FALSE	FALSE	FALSE
	Alloprevotella	-0.38	0	FALSE	FALSE	FALSE	FALSE
	Atopobium spp. unclassified	-0.32	0	FALSE	FALSE	FALSE	FALSE
	Actinotignum	-0.48	0	FALSE	FALSE	FALSE	FALSE
	Ezakiella	-0.52	0	FALSE	FALSE	FALSE	FALSE
	Solobacterium	-0.26	0	FALSE	FALSE	FALSE	FALSE
	Facklamia	-0.35	0	FALSE	FALSE	FALSE	FALSE
	Enhydrobacter	-0.43	0	FALSE	FALSE	FALSE	FALSE
	Alloscardovia	-0.52	0	FALSE	FALSE	FALSE	FALSE
	Propionimicrobium	-0.05	0	FALSE	FALSE	FALSE	FALSE
	Prevotella bergensis	-0.71	0	FALSE	FALSE	FALSE	FALSE
	Ureaplasma	-0.79	0	FALSE	FALSE	FALSE	FALSE
	Lawsonella	0.17	0	FALSE	FALSE	FALSE	FALSE
	Arcanobacterium	-0.85	1	FALSE	FALSE	FALSE	FALSE
	Lactobacillus iners	-1.25	0	FALSE	FALSE	FALSE	FALSE
	Varibaculum	-1.04	0	FALSE	FALSE	FALSE	FALSE
	Escherichia/Shigella	-2.00	0	FALSE	FALSE	FALSE	FALSE

CLR, centre log-ratio transformed relative abundance; Inf = indicates a taxa that was identified as a structural zero

[a]Specimens collected at day-0 and day-8 have been excluded from this analysis. Recurrence was defined as 3-4 Amsel criteria and NS=4-10 during the follow-up period

[b]CLR-mean difference represents the difference in the mean-CLR transformed abundance of a taxon between recurrence and cure cases

[c]W statistic - the number of comparisons in which the null hypothesis as rejected for that taxon

[d]A value of TRUE indicates that the null hypothesis was rejected in ≥90% of comparisons for that taxon

[e]A value of TRUE indicates that the null hypothesis was rejected in ≥80% of comparisons for that taxon

[f]A value of TRUE indicates that the null hypothesis was rejected in ≥70% of comparisons for that taxon

[g]A value of TRUE indicates that the null hypothesis was rejected in ≥60% of comparisons for that taxon
