# Supplementary material for: A Prospective, Open-Label Pilot Study of Concurrent Male Partner Treatment for Bacterial Vaginosis
Source: mBio. 2021 Oct 19;12(5):e02323-21. doi: 10.1128/mBio.02323-21 (PMC8524345; doi:10.1128/mBio.02323-21)
Supplement: TABLE S5 [file mbio.02323-21-st005.pdf]

Table S5. Correlation of specific bacterial taxa between the genital microbiota of sexual partners longitudinally, stratified by BV recurrence status

	**Cure cases longitudinal[a]**				**Recurrence cases longitudinal[b]**			
	**Vaginal/Penile[c]**		**Vaginal/Urethral**		**Vaginal/Penile[c]**		**Vaginal/Urethral**	
**Taxon**	**Corr**	**P-value**	**Corr**	**P-value**	**Corr**	**P-value**	**Corr**	**P-value**
Aerococcus	-	-	-	-	0.678	0.1102	0.6474	0.1172
Anaerococcus	0.121	0.3387	-0.2141	0.0719	-0.6097	0.3806	-0.3777	0.5534
Atopobium vaginae	0.0588	0.4216	0.1783	0.033	-	-	-	-
Candidatus Lachnocurva vaginae	-	-	-	-	-	-	-	-
BVAB-2	-0.0204	0.7812	0.2487	0.008	0.1949	0.6603	-	-
Corynebacterium	**0.339**	**0.004**	-0.1708	0.1748	-	-	-	-
Dialister	0.1966	0.0919	0.1051	0.3706	0.4554	0.4795	0.6013	0.2867
Enterobacter	-	-	-	-	-	-	-	-
Enterococcus	**0.3297**	**0.001**	0.2139	0.032	0.143	0.7596	0.0017	0.9961
Escherichia/Shigella	0.2481	0.025	0.0527	0.6344	0.173	0.7478	0.4172	0.4103
Finegoldia	-0.0087	0.9461	-0.104	0.3936	-0.4885	0.4475	-0.0069	0.9899
Gardnerella	**0.3495**	**0.003**	0.2976	0.009	-0.7743	0.5634	-0.9361	0.5374
Lactobacillus crispatus	**0.4677**	**0.001**	**0.3299**	**0.003**	-	-	-	-
Lactobacillus gasseri	**0.413**	**0.001**	0.2178	0.026	-	-	-	-
Lactobacillus iners	**0.3967**	**0.001**	**0.3152**	**0.014**	-0.3783	0.5474	**-0.9563**	**0.0399**
Lactobacillus jensenii	**0.3752**	**0.001**	0.1316	0.0809	-	-	-	-
Megasphaera	0.0076	0.9091	-	-	-	-	-	-
Peptoniphilus	0.0824	0.5035	-0.1825	0.1449	-0.4508	0.4685	-0.0386	0.946
Prevotella unclassified spp.	0.1942	0.1159	0.1075	0.3696	0.3471	0.5824	0.8418	0.0729
Prevotella bivia	0.1384	0.2398	-0.0051	0.976	0.6383	0.2607	0.7131	0.1778
Prevotella disiens	0.299	0.012	0.1426	0.2008	0.0182	0.9829	0.7521	0.1328
Prevotella timonensis	**0.3751**	**0.001**	0.2322	0.03	0.8648	0.0608	**0.9662**	**0.0079**
Pseudomonas	-	-	-	-	-	-	-	-
Sneathia unclassified spp.	0.018	0.7952	0.0805	0.2827	-	-	0.6504	0.1029
Sneathia amnii	0.0983	0.2318	**0.3371**	**0.005**	0.6107	0.2266	0.8521	0.0579
Sneathia sanguinegens	0.1509	0.044	0.2266	0.014	-0.0249	0.9698	-0.0793	0.9099
Staphylococcus	0.2773	0.019	-0.0039	0.969	0.6512	0.131	0.7155	0.1121
Streptococcus	0.2151	0.0649	0.0497	0.6893	-0.0853	0.8613	0.7438	0.0611
Ureaplasma	0.116	0.2957	0.1738	0.1828	0.0866	0.8809	0.1245	0.8549

Corr, SparCC correlation coefficient; - indicates the taxa was not detected in one or more specimen type for that study time point. The 10 ten most abundant bacteria at each site and 8 bacteria previously associated with BV are presented in this table. Correlations with an absolute correlation coefficient >0.3 and P<0.05 were considered significant and have been bolded in this table

[a] Includes 63 vaginal, 63 cutaneous penile and 63 urethral specimens from 23 couples where the female was cured

[b] Includes 5 vaginal, 5 cutaneous penile and 5 urethral specimens from 4 couples where the female experienced BV recurrence during the follow-up period

[c] Penile refers to cutaneous penile specimens
