# Supplementary material for: A Prospective, Open-Label Pilot Study of Concurrent Male Partner Treatment for Bacterial Vaginosis
Source: mBio. 2021 Oct 19;12(5):e02323-21. doi: 10.1128/mBio.02323-21 (PMC8524345; doi:10.1128/mBio.02323-21)
Supplement: TABLE S6 [file mbio.02323-21-st006.pdf]

Table S6. List of controls processed alongside biological specimens

Control type	Details
Reagent/Extraction Controls	PBS<br>AssayAssure® Genelock (SierraMolecular, USA)<br>Ultrapure water
PCR negative controls	
Positive control	BEI resources HM-276D Genomic DNA from Microbial Mock Community B<br>ZymoBIOMICS™ Microbial Community Standard Catalog No. D6300
