# Supplementary material for: A Prospective, Open-Label Pilot Study of Concurrent Male Partner Treatment for Bacterial Vaginosis
Source: mBio. 2021 Oct 19;12(5):e02323-21. doi: 10.1128/mBio.02323-21 (PMC8524345; doi:10.1128/mBio.02323-21)
Supplement: TABLE S7 [file mbio.02323-21-st007.pdf]

Table S7. ASVs identified as potential contaminants

ASV no.		Phylum	Class	Order	Family	Genus	Total prevalence (%)	Total read count	Vaginal prevalence (%)	Vaginal read count	Cutaneous penile prevalence (%)	Cutaneous penile read count	Urine prevalence (%)	Urine read count	Negative control prevalence (%)	Negative control read count	Notes	Sequence
ASV2	Bacteria	Proteobacteria	Gammaproteobacteria	Burkholderiales	Burkholderiaceae	Burkholderia-Caballeronia-Paraburkholderia	36	737907	6	85	17	6176	87	630598	51	101048	*Excluded as identified as highly abundant in AssayAssure® Genelock (urine stabilisation medium)	TGGGGAATTTTGGACAATGGGCGAAAGCCTGATCCAGCAATGCCGCGTGTGTGAAGAAGGCCTTCGGGTTGTAAAGCACTTTTGTCCGGAAAGAAATCCTTGGCTCTAATACAGTCGGGGGATGACGGTACCGGAAGAATAAGCACCGGCTAACTACGTGCCAGCAGCCGCGGTAATACGTAGGGTGCGAGCGTTAATCGGAATTACTGGGCGTAAAGCGTGCGCAGGCGGTTTGCTAAGACCGATGTGAAATCCCCGGGCTCAACCTGGGAACTGCATTGGTGACTGGCAGGCTAGAGTATGGCAGAGGGGGGTAGAATTCCACGTGTAGCAGTGAAATGCGTAGAGATGTGGAGGAATACCGATGGCGAAGGCAGCCCCCTGGGCCAATACTGACGCTCATGCACGAAAGCGTGGGGAGCAAACA
ASV5	Bacteria	Proteobacteria	Gammaproteobacteria	Pseudomonadales	Pseudomonadaceae	Pseudomonas	48	446900	22	376	45	46620	68	45644	95	354260		TGGGGAATATTGGACAATGGGCGAAAGCCTGATCCAGCCATGCCGCGTGTGTGAAGAAGGTCTTCGGATTGTAAAGCACTTTAAGTTGGGAGGAAGGGCAGTAAATTAATACTTTGCTGTTTTGACGTTACCGACAGAATAAGCACCGGCTAACTCTGTGCCAGCAGCCGCGGTAATACAGAGGGTGCAAGCGTTAATCGGAATTACTGGGCGTAAAGCGCGCGTAGGTGGTTCGTTAAGTTGGATGTGAAATCCCCGGGCTCAACCTGGGAACTGCATTCAAAACTGACGAGCTAGAGTATGGTAGAGGGTGGTGGAATTTCCTGTGTAGCGGTGAAATGCGTAGATATAGGAAGGAACACCAGTGGCGAAGGCGACCACCTGGACTGATACTGACACTGAGGTGCGAAAGCGTGGGGAGCAAACA
ASV44	Bacteria	Proteobacteria	Alphaproteobacteria	Rhizobiales	Rhizobiaceae	Allorhizobium-Neorhizobium-Pararhizobium-Rhizobium	17	47528	0	0	0	0	55	32894	12	14634	*Excluded as identified as highly abundant in AssayAssure® Genelock (urine stabilisation medium)	TGGGGAATATTGGACAATGGGCGCAAGCCTGATCCAGCCATGCCGCGTGAGTGATGAAGGCCCTAGGGTTGTAAAGCTCTTTCACCGGAGAAGATAATGACGGTATCCGGAGAAGAAGCCCCGGCTAACTTCGTGCCAGCAGCCGCGGTAATACGAAGGGGGCTAGCGTTGTTCGGAATTACTGGGCGTAAAGCGCACGTAGGCGGATCGATCAGTCAGGGGTGAAATCCCAGGGCTCAACCCTGGAACTGCCTTTGATACTGTCGATCTGGAGTATGGAAGAGGTGAGTGGAATTCCGAGTGTAGAGGTGAAATTCGTAGATATTCGGAGGAACACCAGTGGCGAAGGCGGCTCACTGGTCCATTACTGACGCTGAGGTGCGAAAGCGTGGGGAGCAAACA
ASV121	Bacteria	Actinobacteriota	Actinobacteria	Micrococcales	Microbacteriaceae	Leifsonia	13	13937	0	0	0	0	43	9901	10	4036	*Excluded as identified as highly abundant in AssayAssure® Genelock (urine stabilisation medium)	TGGGGAATATTGCACAATGGGCGCAAGCCTGATGCAGCAACGCCGCGTGAGGGATGACGGCCTTCGGGTTGTAAACCTCTTTTAGTAGGGAAGAAGCGAAAGTGACGGTACCTGCAGAAAAAGCACCGGCTAACTACGTGCCAGCAGCCGCGGTAATACGTAGGGTGCGAGCGTTGTCCGGAATTATTGGGCGTAAAGAGCTCGTAGGCGGTCTGTCGCGTCTGCTGTGAAAACCCGAGGCTCAACCTCGGGCCTGCAGTGGGTACGGGCAGACTAGAGTGCGGTAGGGGAGAATGGAATTCCTGGTGTAGCGGTGGAATGCGCAGATATCAGGAGGAACACCGATGGCGAAGGCAGTTCTCTGGGCCGTAACTGACGCTGAGGAGCGAAAGCGTGGGGAGCGAACA
ASV26	Bacteria	Proteobacteria	Gammaproteobacteria	Pseudomonadales	Pseudomonadaceae	Pseudomonas	25	96086	2	351	25	34426	36	20065	85	41244		TGGGGAATATTGGACAATGGGCGAAAGCCTGATCCAGCCATGCCGCGTGTGTGAAGAAGGTCTTCGGATTGTAAAGCACTTTAAGTTGGGAGGAAGGGTTGTAGATTAATACTCTGCAATTTTGACGTTACCGACAGAATAAGCACCGGCTAACTCTGTGCCAGCAGCCGCGGTAATACAGAGGGTGCAAGCGTTAATCGGAATTACTGGGCGTAAAGCGCGCGTAGGTGGTTTGTTAAGTTGGATGTGAAATCCCCGGGCTCAACCTGGGAACTGCATTCAAAACTGACTGACTAGAGTATGGTAGAGGGTGGTGGAATTTCCTGTGTAGCGGTGAAATGCGTAGATATAGGAAGGAACACCAGTGGCGAAGGCGACCACCTGGACTAATACTGACACTGAGGTGCGAAAGCGTGGGGAGCAAACA
ASV120	Bacteria	Proteobacteria	Alphaproteobacteria	Sphingomonadales	Sphingomonadaceae	Novosphingobium	17	13813	0	0	13	1419	31	1783	51	10611		TGGGGAATATTGGACAATGGGCGCAAGCCTGATCCAGCAATGCCGCGTGAGTGATGAAGGCCTTCGGGTCGTAAAGCTCTTTTACCAGGGATGATAATGACAGTACCTGGAGAATAAGCTCCGGCTAACTCCGTGCCAGCAGCCGCGGTAATACGGAGGGAGCTAGCGTTGTTCGGAATTACTGGGCGTAAAGCGCACGTAGGCGGTTACTCAAGTCAGAGGTGAAAGCCCGGGGCTCAACCCCGGAACTGCCTTTGAAACTAGGTGACTGGAATCTTGGAGAGGCGAGTGGAATTCCGAGTGTAGAGGTGAAATTCGTAGATATTCGGAAGAACACCAGTGGCGAAGGCGACTCGCTGGACAAGTATTGACGCTGAGGTGCGAAAGCGTGGGGAGCAAACA
ASV100	Bacteria	Firmicutes	Bacilli	Bacillales	Bacillaceae	Bacillus	19	16653	0	0	16	1167	26	2187	83	13299		TAGGGAATCTTCCGCAATGGACGAAAGTCTGACGGAGCAACGCCGCGTGAGTGATGAAGGTTTTCGGATCGTAAAGCTCTGTTGTTAGGGAAGAACAAGTGCCGTTTGAATAAGGCGGCACCTTGACGGTACCTAACCAGAAAGCCACGGCTAACTACGTGCCAGCAGCCGCGGTAATACGTAGGTGGCAAGCGTTGTCCGGAATTATTGGGCGTAAAGCGCGCGCAGGCGGTCTCTTAAGTCTGATGTGAAAGCCCACGGCTCAACCGTGGAGGGTCATTGGAAACTGGGAGACTTGAGTACAGAAGAGGAGAGTGGAATTCCACGTGTAGCGGTGAAATGCGTAGATATGTGGAGGAACACCAGTGGCGAAGGCGACTCTCTGGTCTGTAACTGACGCTGAGGCGCGAAAGCGTGGGGAGCAAACA
ASV110	Bacteria	Proteobacteria	Gammaproteobacteria	Enterobacterales	Yersiniaceae	Serratia	13	15471	1	9	15	2211	17	1130	39	12121		TGGGGAATATTGCACAATGGGCGCAAGCCTGATGCAGCCATGCCGCGTGTGTGAAGAAGGCCTTCGGGTTGTAAAGCACTTTCAGCGAGGAGGAAGGGTTCAGTGTTAATAGCACTGTTCATTGACGTTACTCGCAGAAGAAGCACCGGCTAACTCCGTGCCAGCAGCCGCGGTAATACGGAGGGTGCAAGCGTTAATCGGAATTACTGGGCGTAAAGCGCACGCAGGCGGTTTGTTAAGTCAGATGTGAAATCCCCGCGCTTAACGTGGGAACTGCATTTGAAACTGGCAAGCTAGAGTCTTGTAGAGGGGGGTAGAATTCCAGGTGTAGCGGTGAAATGCGTAGAGATCTGGAGGAATACCGGTGGCGAAGGCGGCCCCCTGGACAAAGACTGACGCTCAGGTGCGAAAGCGTGGGGAGCAAACA
ASV227	Bacteria	Proteobacteria	Gammaproteobacteria	Burkholderiales	Comamonadaceae	Curvibacter	10	5564	0	0	8	436	16	262	44	4866		TGGGGAATTTTGGACAATGGGCGCAAGCCTGATCCAGCCATGCCGCGTGCAGGATGAAGGCCTTCGGGTTGTAAACTGCTTTTGTACGGAACGAAAAGGTTTGACTTAATACGTTGGACTCATGACGGTACCGTAAGAATAAGCACCGGCTAACTACGTGCCAGCAGCCGCGGTAATACGTAGGGTGCGAGCGTTAATCGGAATTACTGGGCGTAAAGCGTGCGCAGGCGGTTATGTAAGACAGATGTGAAATCCCCGGGCTCAACCTGGGAACTGCATTAGTGACTGCATAGCTGGAGTACGGCAGAGGGGGATGGAATTCCGCGTGTAGCAGTGAAATGCGTAGATATGCGGAGGAACACCGATGGCGAAGGCAATCCCCTGGGCCTGTACTGACGCTCATGCACGAAAGCGTGGGGAGCAAACA
ASV257	Bacteria	Bacteroidota	Bacteroidia	Cytophagales	Spirosomaceae	Flectobacillus	8	4795	0	0	7	725	14	467	22	3603		TAGGGAATATTGGGCAATGGACGGAAGTCTGACCCAGCCATGCCGCGTGCAGGATGAAGGCGTTATGCGTTGTAAACTGCTTTTTTATAGGAAGAAGGAGACCTTGCGAGGTTTGGTGACGGTACTATAAGAATAAGCACCGGCTAACTCCGTGCCAGCAGCCGCGGTAATACGGAGGGTGCGAGCGTTGTCCGGATTTATTGGGTTTAAAGGGTGCGTAGGCGGTTTATTAAGTCAGTGGTGAAAGACGGTCGCTCAACGATTGCAGTGCCATTGATACTGGTAGACTTGAGTTCGTATGAGGTAGCTGGAATGGATAGTGTAGCGGTGAAATGCATAGATATTATCCAGAACTCCAATTGCGTAGGCAGGTTACTAATACGATACTGACGCTGATGCACGAAAGTGTGGGGATCAAACA
ASV205	Bacteria	Bacteroidota	Bacteroidia	Flavobacteriales	Flavobacteriaceae	Flavobacterium	8	6467	0	0	6	278	14	708	29	5481		TGAGGAATATTGGTCAATGGTCGCAAGACTGAACCAGCCATGCCGCGTGCAGGATGAAGCATCTATGGTGTGTAAACTGCTTTTGTACGGGAAGAAACACTCCTACGAGTAGGAGCTTGACGGTACCGTAAGAATAAGGATCGGCTAACTCCGTGCCAGCAGCCGCGGTAATACGGAGGATCCAAGCGTTATCCGGAATCATTGGGTTTAAAGGGTCCGTAGGCGGCTTTATAAGTCAGTGGTGAAATCCGGCAGCTTAACTGTCGAATTGCCATTGATACTGTAGGGCTTGAATTGTTGTGAAGTAACTAGAATATGTAGTGTAGCGGTGAAATGCTTAGATATTACATGGAATACCAATTGCGAAGGCAGGTTACTAACAATACATTGACGCTGATGGACGAAAGCGTGGGTAGCGAACA
ASV354	Bacteria	Proteobacteria	Alphaproteobacteria	Paracaedibacterales	Paracaedibacteraceae	Candidatus_Finniella	7	3056	1	2	2	164	14	489	29	2401		TGGGGAATATTGGACAATGGGGGCAACCCTGATCCAGCAATGCCGCGTGAGTGATGAAGGCCTTCGGGTTGTAAAGCTCTTTTGTCAGGGACGATAATGACGGTACCTGACGAATAAGCTCCGGCTAACTTCGTGCCAGCAGCCGCGGTAATACGAAGGGAGCTAGCGTTGTTCGGAATTACTGGGCGTAAAGGGCGCGTAGGCGGTCTTATAAGTTTGGTGTGAAAGCCCTGGGCTTAACCTAGGAACAGCATTGAATACTGTAAGACTTGAGACCGAGAGAGGGAAATGGAATTGCGAGTGTAGAGGTGAAATTCGTAGATATTCGCAAGAACACCAGTGGCGAAGGCGATTTCCTGGCTCGGTACTGACGCTAAGGCGCGAAAGCGTGGGGAGCAAACA
ASV202	Bacteria	Firmicutes	Bacilli	Bacillales	Bacillaceae	Bacillus	7	6841	0	0	3	173	12	380	34	6288		TAGGGAATCTTCCACAATGGACGAAAGTCTGATGGAGCAACGCCGCGTGAGCGATGAAGGCCTTCGGGTCGTAAAGCTCTGTTGTTAGGGAAGAACAAGTGCGAGAGTAACTGCTCGCACCTTGACGGTACCTAACCAGAAAGCCACGGCTAACTACGTGCCAGCAGCCGCGGTAATACGTAGGTGGCAAGCGTTGTCCGGAATTATTGGGCGTAAAGCGCGCGCAGGCGGTTTCTTAAGTCTGATGTGAAAGCCCACGGCTCAACCGTGGAGGGTCATTGGAAACTGGGAGACTTGAGTGCAGAAGAGGAGAGTGGAATTCCACGTGTAGCGGTGAAATGCGTAGAGATGTGGAGGAACACCAGTGGCGAAGGCGACTCTCTGGTCTGTAACTGACGCTGAGGCGCGAAAGCGTGGGGAGCAAACA
ASV141	Bacteria	Proteobacteria	Gammaproteobacteria	Pseudomonadales	Pseudomonadaceae	Pseudomonas	9	11465	0	0	10	1129	10	704	37	9632		TGGGGAATATTGGACAATGGGCGAAAGCCTGATCCAGCCATGCCGCGTGTGTGAAGAAGGTCTTCGGATTGTAAAGCACTTTAAGTTGGGAGGAAGGGCAGTTACCTAATACGTGATTGTTTTGACGTTACCGACAGAATAAGCACCGGCTAACTCTGTGCCAGCAGCCGCGGTAATACAGAGGGTGCAAGCGTTAATCGGAATTACTGGGCGTAAAGCGCGCGTAGGTGGTTTGTTAAGTTGGATGTGAAATCCCCGGGCTCAACCTGGGAACTGCATTCAAAACTGACTGACTAGAGTATGGTAGAGGGTGGTGGAATTTCCTGTGTAGCGGTGAAATGCGTAGATATAGGAAGGAACACCAGTGGCGAAGGCGACCACCTGGACTAATACTGACACTGAGGTGCGAAAGCGTGGGGAGCAAACA
ASV583	Bacteria	Proteobacteria	Alphaproteobacteria	SAR11_clade	Clade_I	Clade_Ia	12	1110	16	377	8	171	9	399	24	163		TGGGGAATCTTGCACAATGGAGGAAACTCTGATGCAGCGATGCCGCGTGAGTGAAGAAGGCCCTTGGGTTGTAAAGCTCTTTCGTCGGGGAAGAAAATGACTGTACCCGAATAAGAAGGTCCGGCTAACTTCGTGCCAGCAGCCGCGGTAATACGAAGGGACCTAGCGTAGTTCGGAATTACTGGGCTTAAAGAGTTCGTAGGTGGTTGAAAAAGTTAGTGGTGAAATCCCAGAGCTTAACTCTGGAACTGCCATTAAAACTTTTCAGCTAGAGTATGATAGAGGAAAGCAGAATTTCTAGTGTAGAGGTGAAATTCGTAGATATTAGAAAGAATACCAATTGCGAAGGCAGCTTTCTGGATCATTACTGACACTGAGGAACGAAAGCATGGGTAGCGAAGA

Table S7. ASVs identified as potential contaminants

ASV no.		Phylum	Class	Order	Family	Genus	Total prevalence (%)	Total read count	Vaginal prevalence (%)	Vaginal read count	Cutaneous penile prevalence (%)	Cutaneous penile read count	Urine prevalence (%)	Urine read count	Negative control prevalence (%)	Negative control read count	Notes	Sequence
ASV321	Bacteria	Proteobacteria	Gammaproteobacteria	Enterobacterales	Yersiniaceae	Serratia	7	3548	1	6	7	400	9	364	24	2778		TGGGGAATATTGCACAATGGGCGCAAGCCTGATGCAGCCATGCCGCGTGTGTGAAGAAGGCCTTCGGGTTGTAAAGCACTTTCAGCGAGGAGGAAGGGCAGTGTGTTAATAGCACATTGCATTGACGTTACTCGCAGAAGAAGCACCGGCTAACTCCGTGCCAGCAGCCGCGGTAATACGGAGGGTGCAAGCGTTAATCGGAATTACTGGGCGTAAAGCGCACGCAGGCGGTTTGTTAAGTCAGATGTGAAATCCCCGCGCTTAACGTGGGAACTGCATTTGAAACTGGCAAGCTAGAGTCTTGTAGAGGGGGGTAGAATTCCAGGTGTAGCGGTGAAATGCGTAGAGATCTGGAGGAATACCGGTGGCGAAGGCGGCCCCCTGGACAAAGACTGACGCTCAGGTGCGAAAGCGTGGGGAGCAAACA
ASV277	Bacteria	Firmicutes	Bacilli	Erysipelotrichales	Erysipelotrichaceae	Erysipelothrix	7	4345	0	0	5	222	9	289	37	3834		TAGGGAATTTTCGGCAATGGGGGAAACCCTGACCGAGCAACGCCGCGTGAGTGAAGAAGGCCTTCGGGTTGTAAAGCTCTGTTGTAAGGGAAGAACGATAGTTAGAGGGAATGCTAGCTATATGACGGTACCTTACCAGAAAGCCACGGCTAACTACGTGCCAGCAGCCGCGGTAATACGTAGGTGGCAAGCGTTATCCGGAATTATTGGGCGTAAAGGGAGCGCAGGCGGTTTATTAAGTTTATGGTGAAAGTTCGGGGCTCAACCCCGTGATGCCATAGAAACTGGTAGACTTGAGTACAGGAGAGGTTAGTGGAATTCCATGTGTAGCGGTAAAATGCGTAGATATATGGAGGAACACCAGTGGCGAAGGCGGCTAACTGGCCTGTAACTGACGCTGAGGCTCGAAAGCGTGGGGAGCAAATA
ASV926	Bacteria	Proteobacteria	Gammaproteobacteria	SAR86_clade	NA	NA	8	423	10	173	5	60	8	158	10	32		TGGGGAATATTGGACAATGGGCGCAAGCCTGATCCAGCCATACCGCGTGTGTGAAGAAGGCCTTCGGGTTGTAAAGCACTTTAAGCAGGGAGAAAAAGTTATAAGTTAATACCTTATAACCCTGATGTTACCTGCAGAATAAGCACCGGCTAATTCCGTGCCAGCAGCCGCGGTAATACGGAAGGTGCAAGCGTTAATCGGAATTACTGGGCGTAAAGCGCGCGTAGGTGGTTTGTTAAGTTGGATGTGAAAGCCCTGGGCTCAACCTAGGAACTGCATCCAAAACTAACTCACTAGAGTACGATAGAGGGAGGTAGAATTCATAGTGTAGCGGTGGAATGCGTAGATATTATGAAGAATACCAGTGGCGAAGGCGGCCTCCTGGATCTGTACTGACACTGAGGTGCGAAAGCGTGGGTAGCGAACA
ASV1183	Bacteria	Bacteroidota	Bacteroidia	Flavobacteriales	Flavobacteriaceae	Formosa	6	235	3	32	5	59	6	101	15	43		TGAGGAATATTGGACAATGGAGGCAACTCTGATCCAGCCATGCCGCGTGCAGGAAGACTGCCCTATGGGTTGTAAACTGCTTTTATATAGGAAGAAACCTACTTACGTGTAAGTAGCTGACGGTACTATAAGAATAAGGATCGGCTAACTCCGTGCCAGCAGCCGCGGTAATACGGAGGATCCAAGCGTTATCCGGAATCATTGGGTTTAAAGGGTCCGTAGGTGGATAATTAAGTCAGAGGTGAAATCTTGCAGCTCAACTGTAAAATTGCCTTTGATACTGGTTATCTTGAGTTATTATGAAGTAGTTAGAATATGTAGTGTAGCGGTGAAATGCATAGATATTACATAGAATACCAATTGCGAAGGCAGATTACTAATAATCAACTGACACTGATGGACGAAAGCGTGGGTAGCGAACA
ASV143	Bacteria	Proteobacteria	Alphaproteobacteria	Rhizobiales	Rhizobiaceae	Allorhizobium-Neorhizobium-Pararhizobium-Rhizobium	6	11444	2	14	8	1047	6	744	17	9639		TGGGGAATATTGGACAATGGGCGCAAGCCTGATCCAGCCATGCCGCGTGAGTGATGAAGGCCTTAGGGTTGTAAAGCTCTTTCACCGGAGAAGATAATGACGGTATCCGGAGAAGAAGCCCCGGCTAACTTCGTGCCAGCAGCCGCGGTAATACGAAGGGGGCTAGCGTTGTTCGGAATTACTGGGCGTAAAGCGCACGTAGGCGGATATTTAAGTCAGGGGTGAAATCCCAGAGCTCAACTCTGGAACTGCCTTTGATACTGGGTATCTTGAGTATGGAAGAGGTAAGTGGAATTCCGAGTGTAGAGGTGAAATTCGTAGATATTCGGAGGAACACCAGTGGCGAAGGCGGCTTACTGGTCCATTACTGACGCTGAGGTGCGAAAGCGTGGGGAGCAAACA
ASV438	Bacteria	Proteobacteria	Gammaproteobacteria	Oceanospirillales	Halomonadaceae	Halomonas	4	2091	0	0	5	380	5	100	15	1611		TGGGGAATATTGGACAATGGGCGCAAGCCTGATCCAGCCATGCCGCGTGTGTGAAGAAGGCCCTCGGGTTGTAAAGCACTTTCAGTGGGGAAGAAAGCCTGGTGGTTAATACCCGTCAGGAAGGACATCACCCACAGAAGAAGCACCGGCTAACTCCGTGCCAGCAGCCGCGGTAATACGGAGGGTGCGAGCGTTAATCGGAATTACTGGGCGTAAAGCGCGCGTAGGTGGCTTGATAAGCCGGTTGTGAAAGCCCCGGGCTCAACCTGGGAACGGCATCCGGAACTGTCAGGCTAGAGTGCAGGAGAGGAAGGTAGAATTCCCGGTGTAGCGGTGAAATGCGTAGAGATCGGGAGGAATACCAGTGGCGAAGGCGGCCTTCTGGACTGACACTGACACTGAGGTGCGAAAGCGTGGGTAGCAAACA
ASV251	Bacteria	Proteobacteria	Gammaproteobacteria	Enterobacterales	Yersiniaceae	Serratia	5	4967	0	0	5	3066	5	297	20	1604		TGGGGAATATTGCACAATGGGCGCAAGCCTGATGCAGCCATGCCGCGTGTGTGAAGAAGGCCTTCGGGTTGTAAAGCACTTTCAGCGAGGAGGAAGGGTAGTGTGTTAATAGCACATTGCATTGACGTTACTCGCAGAAGAAGCACCGGCTAACTCCGTGCCAGCAGCCGCGGTAATACGGAGGGTGCAAGCGTTAATCGGAATTACTGGGCGTAAAGCGCACGCAGGCGGTTTGTTAAGTCAGATGTGAAATCCCCGCGCTTAACGTGGGAACTGCATTTGAAACTGGCAAGCTAGAGTCTTGTAGAGGGGGGTAGAATTCCAGGTGTAGCGGTGAAATGCGTAGAGATCTGGAGGAATACCGGTGGCGAAGGCGGCCCCCTGGACAAAGACTGACGCTCAGGTGCGAAAGCGTGGGGAGCAAACA
ASV1164	Bacteria	Proteobacteria	Alphaproteobacteria	Rhodobacterales	Rhodobacteraceae	Ascidiaceihabitans	5	245	6	74	3	36	5	99	12	36		TGGGGAATCTTAGACAATGGGCGCAAGCCTGATCTAGCCATGCCGCGTGAGTGATGAAGGCCTTAGGGTCGTAAAGCTCTTTCGCCTGTGATGATAATGACAGTAGCAGGTAAAGAAACCCCGGCTAACTCCGTGCCAGCAGCCGCGGTAATACGGAGGGGGTTAGCGTTGTTCGGAATTACTGGGCGTAAAGCGCACGTAGGCGGATTGGAAAGTTGGGGGTGAAATCCCGGGGCTCAACCCCGGAACTGCCTCCAAAACTATCAGTCTAGAGTTCGAGAGAGGTGAGTGGAATTCCAAGTGTAGAGGTGAAATTCGTAGATATTTGGAGGAACACCAGTGGCGAAGGCGGCTCACTGGCTCGATACTGACGCTGAGGTGCGAAAGTGTGGGGAGCAAACA
ASV692	Bacteria	Patescibacteria	Saccharimonadia	Saccharimonadales	NA	NA	3	811	0	0	2	21	5	267	7	523		TGAGGAATCTTCCACAATGGGCGAAAGCCTGATGGAGCAACGCCGCGTGCAGGATGAAGGCCCTCGGGTCGTAAACTGCTTTTATAAGTGAAGAATATGACGGTAACTTATGAATAAGCACCGGCTAACTACGTGCCAGCAGCCGCGGTCATACGTAGGGTGCAAGCATTATCCGGAGTGACTGGGCGTAAAGAGTTGCGTAGGTGGTTTGTTAAGTAGGTAGTGAAATCTGGCGGCTCAACCGTACAGGCTATTACCTAAACTGGCAAACTCGAGAATGGTAGAGGTAACTGGAATTTCTTGTGTAGGAGTGAAATCCGTAGATATAAGAAGGAACACCAATGGCGTAGGCAGGTTACTGGACCATTTCTGACACTAAGGCACGAAAGCGTGGGGAGCGAACG
ASV611	Bacteria	Proteobacteria	Gammaproteobacteria	Pseudomonadales	Pseudomonadaceae	Pseudomonas	3	1016	0	0	1	61	5	210	12	745		TGGGGAATATTGGACAATGGGCGAAAGCCTGATCCAGCCATGCCGCGTGTGTGAAGAAGGTCTTCGGATTGTAAAGCACTTTAAGTTGGGAGGAAGGGCAGTAAGCGAATACCTTGCTGTTTTGACGTTACCGACAGAATAAGCACCGGCTAACTCTGTGCCAGCAGCCGCGGTAATACAGAGGGTGCAAGCGTTAATCGGAATTACTGGGCGTAAAGCGCGCGTAGGTGGTTCGTTAAGTTGGATGTGAAATCCCCGGGCTCAACCTGGGAACTGCATCCAAAACTGGCGAGCTAGAGTAGGGCAGAGGGTGGTGGAATTTCCTGTGTAGCGGTGAAATGCGTAGATATAGGAAGGAACACCAGTGGCGAAGGCGACCACCTGGGCTCATACTGACACTGAGGTGCGAAAGCGTGGGGAGCAAACA
ASV476	Bacteria	Proteobacteria	Gammaproteobacteria	Oceanospirillales	Halomonadaceae	Halomonas	3	1738	1	2	0	0	5	165	17	1571		TGGGGAATATTGGACAATGGGCGCAAGCCTGATCCAGCCATGCCGCGTGTGTGAAGAAGGCCCTCGGGTTGTAAAGCACTTTCAGTGGGGAAGAAAGCCTTCCGGTTAATACCCGGGAGGAAGGACATCACCCACAGAAGAAGCACCGGCTAACTCCGTGCCAGCAGCCGCGGTAATACGGAGGGTGCGAGCGTTAATCGGAATTACTGGGCGTAAAGCGCGCGTAGGTGGCTTGATAAGCCGGTTGTGAAAGCCCCGGGCTCAACCTGGGAACGGCATCCGGAACTGTCAGGCTAGAGTGCAGGAGAGGAAGGTAGAATTCCCGGTGTAGCGGTGAAATGCGTAGAGATCGGGAGGAATACCAGTGGCGAAGGCGGCCTTCTGGACTGACACTGACACTGAGGTGCGAAAGCGTGGGTAGCAAACA
ASV231	Bacteria	Actinobacteriota	Actinobacteria	Corynebacteriales	Nocardiaceae	Rhodococcus	6	5718	0	0	13	1006	5	1127	15	3585		TGGGGAATATTGCACAATGGGCGAAAGCCTGATGCAGCGACGCCGCGTGAGGGATGACGGCCTTCGGGTTGTAAACCTCTTTCAGCAGGGACGAAGCGCAAGTGACGGTACCTGCAGAAGAAGCACCGGCTAACTACGTGCCAGCAGCCGCGGTAATACGTAGGGTGCAAGCGTTGTCCGGAATTACTGGGCGTAAAGAGTTCGTAGGCGGTTTGTCGCGTCGTTTGTGAAAACCAGCAGCTCAACTGCTGGCTTGCAGGCGATACGGGCAGACTTGAGTACTGCAGGGGAGACTGGAATTCCTGGTGTAGCGGTGAAATGCGCAGATATCAGGAGGAACACCGGTGGCGAAGGCGGGTCTCTGGGCAGTAACTGACGCTGAGGAACGAAAGCGTGGGTAGCGAACA
ASV1099	Bacteria	Proteobacteria	Alphaproteobacteria	Rhodobacterales	Rhodobacteraceae	Planktomarina	4	292	2	17	5	93	5	155	10	27		TAGGGAATCTTAGACAATGGGGGCAACCCTGATCTAGCCATGCCGCGTGTGTGATGAAGGCCCTAGGGTCGTAAAGCACTTTCGCCAGAGATGATAATGACAGTATCTGGTAAAGAAACCCCGGCTAACTCCGTGCCAGCAGCCGCGGTAATACGGAGGGGGTTAGCGTTGTTCGGAATTACTGGGCGTAAAGCGCACGTAGGCGGATTAGTAAGTTAGAGGTGAAATCCCAGGGCTCAACCCTGGAACTGCCTTTAATACTGCTAGTCTTGAGTTCGAGAGAGGTAAGTGGAATTCCGAGTGTAGAGGTGAAATTCGTAGATATTCGGAGGAACACCAGTGGCGAAGGCGGCTTACTGGCTCGATACTGACGCTGAGGTGCGAAAGTGTGGGGAGCAAACA
ASV522	Bacteria	Proteobacteria	Alphaproteobacteria	Rhizobiales	Beijerinckiaceae	Methylobacterium-Methylorubrum	3	1360	0	0	4	122	5	115	12	1123		TGGGGAATATTGGACAATGGGCGCAAGCCTGATCCAGCCATGCCGCGTGAGTGATGAAGGCCTTAGGGTTGTAAAGCTCTTTTATCCGGGACGATAATGACGGTACCGGAGGAATAAGCCCCGGCTAACTTCGTGCCAGCAGCCGCGGTAATACGAAGGGGGCTAGCGTTGCTCGGAATCACTGGGCGTAAAGGGCGCGTAGGCGGCGTTTTAAGTCGGGGGTGAAAGCCTGTGGCTCAACCACAGAATGGCCTTCGATACTGGGACGCTTGAGTATGGTAGAGGTTGGTGGAACTGCGAGTGTAGAGGTGAAATTCGTAGATATTCGCAAGAACACCGGTGGCGAAGGCGGCCAACTGGACCATTACTGACGCTGAGGCGCGAAAGCGTGGGGAGCAAACA
ASV1452	Bacteria	Bacteroidota	Bacteroidia	Flavobacteriales	Flavobacteriaceae	NS4_marine_group	5	144	7	64	4	26	5	39	7	15		TAAGGAATATTGGACAATGGACGAAAGTCTGATCCAGCCATGCCGCGTGCAGGATGACAGCCCTACGGGTTGTAAACTGCTTTTATACAGGAAGAAACACTCCGACGTGTCGGAGCTTGACGGTACTGTAAGAATAAGGATCGGCTAACTCCGTGCCAGCAGCCGCGGTAATACGGAGGATCCAAGCGTTATCCGGAATCATTGGGTTTAAAGGGTCCGCAGGCTGTTTTTTAAGTCAGAGGTGAAATCCTACAGCTCAACTGTAGAACTGCCTTTGATACTGGAAAACTTGAGTTATTGTGAAGTGGTTAGAATGTGTGGTGTAGCGGTGAAATGCATAGATATCACACAGAATACCGATTGCGAAGGCAGATCACTAACAATATACTGACGCTCATGGACGAAAGCGTGGGGAGCGAACA

Table S7. ASVs identified as potential contaminants

ASV no.		Phylum	Class	Order	Family	Genus	Total prevalence (%)	Total read count	Vaginal prevalence (%)	Vaginal read count	Cutaneous penile prevalence (%)	Cutaneous penile read count	Urine prevalence (%)	Urine read count	Negative control prevalence (%)	Negative control read count	Notes	Sequence
ASV168	Bacteria	Proteobacteria	Gammaproteobacteria	Pseudomonadales	Pseudomonadaceae	Pseudomonas	5	8725	1	13	6	1137	4	881	29	6694		TGGGGAATATTGGACAATGGGCGAAAGCCTGATCCAGCCATGCCGCGTGTGTGAAGAAGGTCTTCGGATTGTAAAGCACTTTAAGTTGGGAGGAAGGGCAGTAAATTAATACTTTGCTGTTTTGACGTTACCGACAGAATAAGCACCGGCTAACTCTGTGCCAGCAGCCGCGGTAATACAGAGGGTGCAAGCGTTAATCGGAATTACTGGGCGTAAAGCGCGCGTAGGTGGTTCGTTAAGTTGGATGTGAAATCCCCGGGCTCAACCTGGGAACTGCATTCAAAACTGTCGAGCTAGAGTATGGTAGAGGGTGGTGGAATTTCCTGTGTAGCGGTGAAATGCGTAGATATAGGAAGGAACACCAGTGGCGAAGGCGACCACCTGGACTGATACTGACACTGAGGTGCGAAAGCGTGGGGAGCAAACA
ASV208	Bacteria	Proteobacteria	Gammaproteobacteria	Burkholderiales	Comamonadaceae	Delftia	4	6530	1	2	5	4480	4	191	17	1857		TGGGGAATTTTGGACAATGGGCGAAAGCCTGATCCAGCAATGCCGCGTGCAGGATGAAGGCCTTCGGGTTGTAAACTGCTTTTGTACGGAACGAAAAAGCTTCTCCTAATACGAGAGGCCCATGACGGTACCGTAAGAATAAGCACCGGCTAACTACGTGCCAGCAGCCGCGGTAATACGTAGGGTGCGAGCGTTAATCGGAATTACTGGGCGTAAAGCGTGCGCAGGCGGTTATGTAAGACAGATGTGAAATCCCCGGGCTCAACCTGGGAACTGCATTTGTGACTGCATGGCTAGAGTACGGTAGAGGGGGATGGAATTCCGCGTGTAGCAGTGAAATGCGTAGATATGCGGAGGAACACCGATGGCGAAGGCAATCCCCTGGACCTGTACTGACGCTCATGCACGAAAGCGTGGGGAGCAAACA
ASV332	Bacteria	Proteobacteria	Gammaproteobacteria	Burkholderiales	Alcaligenaceae	Advenella	1	3290	0	0	0	0	4	349	2	2941		TGGGGAATTTTGGACAATGGGGGAAACCCTGATCCAGCCATCCCGCGTGTGCGATGAAGGCCTTCGGGTTGTAAAGCACTTTTGTCAGGGAAGAAAAGGTTTCGGATAATACCTGGAACTGATGACGGTACCTGAAGAATAAGCACCGGCTAACTACGTGCCAGCAGCCGCGGTAATACGTAGGGTGCAAGCGTTAATCGGAATTACTGGGCGTAAAGCGTGCGCAGGCGGTTCGGAAAGAAAGATGTGAAATCCCAGGGCTCAACCTTGGAACTGCATTTTTAACTCCCGAACTAGAGTATGTCAGAGGGGGGTGGAATTCCACGTGTAGCAGTGAAATGCGTAGATATGTGGAGGAACACCGATGGCGAAGGCAGCCCCCTGGGATAATACTGACGCTCATGCACGAAAGCGTGGGGAGCAAACA
ASV1434	Bacteria	Proteobacteria	Gammaproteobacteria	Alteromonadales	Alteromonadaceae	Glaciecola	3	144	2	21	5	66	3	47	5	10		TGGGGAATATTGCACAATGGGGGAAACCCTGATGCAGCCATGCCGCGTGTGTGAAGAAGGCCTTCGGGTTGTAAAGCACTTTCAGTTGTGAGGAAAGTTTAGTAGTTAATACCTGCTAGATGTGACGTTAGCAACAGAAGAAGCACCGGCTAACTCCGTGCCAGCAGCCGCGGTAATACGGAGGGTGCGAGCGTTAATCGGAATTACTGGGCGTAAAGCGCACGCAGGCGGTCTGTTAAGCTAGATGTGAAAGCCCCGCGCTCAACGTGGGAGGGTCATTTAGAACTGGCAGACTAGAGTCTTGGAGAGGGGAGTGGAATTCCAGGTGTAGCGGTGAAATGCGTAGAGATCTGGAGGAACATCAATGGCGAAGGCAACTCCCTGGCCAAAGACTGACGCTCATGTGCGAAAGTGTGGGTAGCGAACA
ASV567	Bacteria	Proteobacteria	Gammaproteobacteria	Oceanospirillales	Halomonadaceae	Halomonas	3	1187	0	0	1	16	3	90	17	1081		TGGGGAATATTGGACAATGGGCGCAAGCCTGATCCAGCCATGCCGCGTGTGTGAAGAAGGCCCTCGGGTTGTAAAGCACTTTCAGTGGGGAAGAACGCCTGGTGGTTAATACCCGTCAGGAAGGACATCACCCACAGAAGAAGCACCGGCTAACTCCGTGCCAGCAGCCGCGGTAATACGGAGGGTGCGAGCGTTAATCGGAATTACTGGGCGTAAAGCGCGCGTAGGTGGCTTGATAAGCCGGTTGTGAAAGCCCCGGGCTCAACCTGGGAACGGCATCCGGAACTGTCAGGCTAGAGTGCAGGAGAGGAAGGTAGAATTCCCGGTGTAGCGGTGAAATGCGTAGAGATCGGGAGGAATACCAGTGGCGAAGGCGGCCTTCTGGACTGACACTGACACTGAGGTGCGAAAGCGTGGGTAGCAAACA
ASV429	Bacteria	Proteobacteria	Gammaproteobacteria	Pseudomonadales	Pseudomonadaceae	Pseudomonas	3	2165	0	0	5	397	3	163	17	1605		TGGGGAATATTGGACAATGGGCGAAAGCCTGATCCAGCCATGCCGCGTGTGTGAAGAAGGTCTTCGGATTGTAAAGCACTTTAAGTTGGGAGGAAGGGCAGTTACCTAATACGTAATTGTTTTGACGTTACCGACAGAATAAGCACCGGCTAACTCTGTGCCAGCAGCCGCGGTAATACAGAGGGTGCAAGCGTTAATCGGAATTACTGGGCGTAAAGCGCGCGTAGGTGGTTTGTTAAGTTGGATGTGAAATCCCCGGGCTCAACCTGGGAACTGCATTCAAAACTGACTGACTAGAGTATGGTAGAGGGTGGTGGAATTTCCTGTGTAGCGGTGAAATGCGTAGATATAGGAAGGAACACCAGTGGCGAAGGCGACCACCTGGACTAATACTGACACTGAGGTGCGAAAGCGTGGGGAGCAAACA
ASV242	Bacteria	Proteobacteria	Gammaproteobacteria	Enterobacterales	Enterobacteriaceae	Escherichia/Shigella	4	5114	0	0	4	555	3	59	24	4500		TGGGGAATATTGCACAATGGGCGCAAGCCTGATGCAGCCATGCCGCGTGTATGAAGAAGGCCTTCGGGTTGTAAAGTACTTTCAGCGGGGATGAAGGGAGTAAAGTTAATACCTTTGCTCATTGACGTTACCCGCAGAAGAAGCACCGGCTAACTCCGTGCCAGCAGCCGCGGTAATACGGAGGGTGCAAGCGTTAATCGGAATTACTGGGCGTAAAGCGCACGCAGGCGGTTTGTTAAGTCAGATGTGAAATCCCCGGGCTCAACCTGGGAACTGCATCTGATACTGGCAAGCTTGAGTCTCGTAGAGGGGGGTAGAATTCCAGGTGTAGCGGTGAAATGCGTAGAGATCTGGAGGAATACCGGTGGCGAAGGCGGCCCCCTGGACGAAGACTGACGCTCAGGTGCGAAAGCGTGGGGAGCAAACA
ASV665	Bacteria	Proteobacteria	Gammaproteobacteria	Burkholderiales	Neisseriaceae	NA	3	875	1	27	3	260	3	46	7	542		TGGGGAATTTTGGACAATGGGGGAAACCCTGATCCAGCCATGCCGCGTGTATGAAGAAGGCCTTAGGGTTGTAAAGTACTTTTGTTAGGGAAGAAAAGCTAGTTTTTAATAAAAATTAGTGATGACGGTACCTAAAGAATAAGCACCGGCTAACTACGTGCCAGCAGCCGCGGTAATACGTAGGGTGCAAGCGTTAATCGGAATTATTGGGCGTAAAGCGAGTGCAGACGGTTACTTAAGCCAGATGTGAAATCCCCAAGCTTAACTTGGGACGTGCATTTGGAACTGGGTGACTAGAGTGTGTCAGAGGGAGGTAGAATTCCACATGTAGCGGTGGAATGCGTAGAGATGTGGAGGAATACCGATGGCGAAGGCAGCCTCCTGGGATAACACTGACGTTGAGGCTCGAAAGCGTGGGGAGCAAACA
ASV1205	Bacteria	Bacteroidota	Bacteroidia	Flavobacteriales	Cryomorphaceae	NA	2	245	0	0	3	88	3	135	5	22		TGAGGAATATTGGTCAATGGAGGAAACTCTGAACCAGCCATGCCGCGTGAAGGATGACGGCCCTATGGGTTGTAAACTTCTTTTATAGAGGAAGAAACTTGTCTACGTGTAGACAACTGACGGTACTCTACGAATAAGGATCGGCTAACTCCGTGCCAGCAGCCGCGGTAATACGGAGGATCCAAGCGTTATCCGGATTCATTGGGTTTAAAGGGTCCGTAGGCGGGTCTTTAAGTCAGTGGTGAAAGCCGACAGCTCAACTGTCGAACTGCCATTGATACTGGAGATCTTGAGTACAAATGAAGTAGGCGGAATGAGTCATGTAGCGGTGAAATGCATAGATATGACTCAGAACACCGATTGCGAAGGCAGCTTACTAACATGTAACTGACGCTGAGGGACGAAAGCGTGGGGAGCGAACA
ASV1326	Bacteria	Proteobacteria	Alphaproteobacteria	Puniceispirillales	SAR116_clade	Candidatus_Puniceispirillum	3	174	3	42	3	44	3	66	5	22		TGGGGAATATTGGACAATGGGGGCAACCCTGATCCAGCAATGCCGCGTGTGTGATGAAGGCCTTAGGGTTGTAAAGCACTTTCACTGGTGAAGATGATGACGGTAACCAGAGAAGAAGCCCCGGCTAACTTCGTGCCAGCAGCCGCGGTAATACGAAGGGGGCGAGCGTTGTTCGGAATTACTGGGCGTAAAGGGCGCGTAGGCGGTCTTTTAAGTTAGGCGTGAAAGCCCCGGGCTCAACCCGGGAACTGCGCTTAAGACTGGAAGACTAGAAAACGGAAGAGGGTAGTGGAATTCCCAGTGTAGAGGTGAAATTCGTAGATATTGGGAAGAACACCAGTGGCGAAAGCGGCTACCTGGTCCGATTTTGACGCTGAGGCGCGAAAGCGTGGGGAGCAAACA
ASV335	Bacteria	Proteobacteria	Gammaproteobacteria	Pseudomonadales	Pseudomonadaceae	Pseudomonas	3	3238	0	0	2	226	3	60	17	2952		TGGGGAATATTGGACAATGGGCGAAAGCCTGATCCAGCCATGCCGCGTGTGTGAAGAAGGTCTTCGGATTGTAAAGCACTTTAAGTTGGGAGGAAGGGCAGTTACCTAATACGTGATTGTTTTGACGTTACCGACAGAATAAGCACCGGCTAACTCTGTGCCAGCAGCCGCGGTAATACAGAGGGTGCAAGCGTTAATCGGAATTACTGGGCGTAAAGCGCGCGTAGGTGGTTTGTTAAGTTGGATGTGAAAGCCCCGGGCTCAACCTGGGAACTGCATCCAAAACTGGCAAGCTAGAGTATGGTAGAGGGTGGTGGAATTTCCTGTGTAGCGGTGAAATGCGTAGATATAGGAAGGAACACCAGTGGCGAAGGCGACCACCTGGACTGATACTGACACTGAGGTGCGAAAGCGTGGGGAGCAAACA
ASV556	Bacteria	Firmicutes	Bacilli	Bacillales	Planococcaceae	Lysinibacillus	2	1281	0	0	2	108	3	153	12	1020		TAGGGAATCTTCCACAATGGGCGAAAGCCTGATGGAGCAACGCCGCGTGAGTGAAGAAGGATTTCGGTTCGTAAAACTCTGTTGTAAGGGAAGAACAAGTACAGTAGTAACTGGCTGTACCTTGACGGTACCTTATTAGAAAGCCACGGCTAACTACGTGCCAGCAGCCGCGGTAATACGTAGGTGGCAAGCGTTGTCCGGAATTATTGGGCGTAAAGCGCGCGCAGGTGGTTTCTTAAGTCTGATGTGAAAGCCCACGGCTCAACCGTGGAGGGTCATTGGAAACTGGGAGACTTGAGTGCAGAAGAGGATAGTGGAATTCCAAGTGTAGCGGTGAAATGCGTAGAGATTTGGAGGAACACCAGTGGCGAAGGCGACTATCTGGTCTGTAACTGACACTGAGGCGCGAAAGCGTGGGGAGCAAACA
ASV764	Bacteria	Bacteroidota	Bacteroidia	Flavobacteriales	Cryomorphaceae	NA	5	667	5	70	5	96	2	29	7	472		TGAGGAATATTGGTCAATGGAGGCAACTCTGAACCAGCCATGCCGCGTGAAGGATGACGGCCCTATGGGTTGTAAACTTCTTTTATAGAGGAAGAAACTTGTCTACGTGTAGACAACTGACGGTACTCTACGAATAAGGATCGGCTAACTCCGTGCCAGCAGCCGCGGTAATACGGAGGATCCAAGCGTTATCCGGATTCATTGGGTTTAAAGGGTCCGTAGGCGGGTCTTTAAGTCAGTGGTGAAAGCCGACAGCTCAACTGTCGAACTGCCATTGATACTGGAGACCTTGAGTACAAATGAAGTAGGCGGAATGAGTCATGTAGCGGTGAAATGCATAGATATGACTCAGAACACCGATTGCGAAGGCAGCTTACTAACATGTAACTGACGCTGAGGGACGAAAGCGTGGGGAGCGAACA
ASV262	Bacteria	Firmicutes	Bacilli	Lactobacillales	Carnobacteriaceae	Carnobacterium	3	4665	0	0	4	820	2	198	17	3647		TAGGGAATCTTCCGCAATGGACGAAAGTCTGACGGAGCAACGCCGCGTGAGTGAAGAAGGTTTTCGGATCGTAAAACTCTGTTGTTAAAGAAGAACAAGGATGAGAGTAACTGCTCATCCCCTGACGGTATTTAACCAGAAAGCCACGGCTAACTACGTGCCAGCAGCCGCGGTAATACGTAGGTGGCAAGCGTTGTCCGGATTTATTGGGCGTAAAGCGAGCGCAGGCGGTTCTTTAAGTCTGATGTGAAAGCCCCCGGCTCAACCGGGGAGGGTCATTGGAAACTGGAGAACTTGAGTGCAGAAGAGGAGAGTGGAATTCCACGTGTAGCGGTGAAATGCGTAGATATGTGGAGGAACACCAGTGGCGAAGGCGACTCTCTGGTCTGTAACTGACGCTGAGGCTCGAAAGCGTGGGGAGCAAACA
ASV340	Bacteria	Firmicutes	Bacilli	Bacillales	Bacillaceae	Bacillus	5	3183	0	0	4	106	2	39	39	3038		TAGGGAATCATCCGCAATGGACGAAAGTCTGACGGTGCAACGCCGCGTGAGTGATGAAGGATTTCGGTTCGTAAAGCTCTGTTGTTAGGGAAGAACAAGTGCCGTTCGAATAGGTCGGCACCTTGACGGTACCTAACCAGAAAGCCACGGCTAACTACGTGCCAGCAGCCGCGGTAATACGTAGGTGGCAAGCGTTGTCCGGAATTATTGGGCGTAAAGCGCGCGCAGGCGGTCTTTTAAGTCTGATGTGAAAGCCCCCGGCTCAACCGGGGAGGGTCATTGGAAACTGGGAGACTTGAGTACAGAAGAGGAGAGTGGAATTCCACGTGTAGCGGTGAAATGCGTAGATATGTGGAGGAACACCAGTGGCGAAGGCGACTCTCTGGTCTGTAACTGACGCTGAGGCGCGAAAGCGTGGGGAGCAAACA

Table S7. ASVs identified as potential contaminants

ASV no.		Phylum	Class	Order	Family	Genus	Total prevalence (%)	Total read count	Vaginal prevalence (%)	Vaginal read count	Cutaneous penile prevalence (%)	Cutaneous penile read count	Urine prevalence (%)	Urine read count	Negative control prevalence (%)	Negative control read count	Notes	Sequence
ASV309	Bacteria	Proteobacteria	Gammaproteobacteria	Aeromonadales	Aeromonadaceae	Aeromonas	3	3810	0	0	3	337	2	329	17	3144		TGGGGAATATTGCACAATGGGGGAAACCCTGATGCAGCCATGCCGCGTGTGTGAAGAAGGCCTTCGGGTTGTAAAGCACTTTCAGCGAGGAGGAAAGGTCAGTAGCTAATATCTGCTGGCTGTGACGTTACTCGCAGAAGAAGCACCGGCTAACTCCGTGCCAGCAGCCGCGGTAATACGGAGGGTGCAAGCGTTAATCGGAATTACTGGGCGTAAAGCGCACGCAGGCGGTTGGATAAGTTAGATGTGAAAGCCCCGGGCTCAACCTGGGAATTGCATTTAAAACTGTCCAGCTAGAGTCTTGTAGAGGGGGGTAGAATTCCAGGTGTAGCGGTGAAATGCGTAGAGATCTGGAGGAATACCGGTGGCGAAGGCGGCCCCCTGGACAAAGACTGACGCTCAGGTGCGAAAGCGTGGGGAGCAAACA
ASV160	Bacteria	Proteobacteria	Gammaproteobacteria	Xanthomonadales	Xanthomonadaceae	Stenotrophomonas	2	9717	0	0	3	7661	2	1843	10	213		TGGGGAATATTGGACAATGGGCGCAAGCCTGATCCAGCCATACCGCGTGGGTGAAGAAGGCCTTCGGGTTGTAAAGCCCTTTTGTTGGGAAAGAAATCCAGCCGGCTAATACCTGGTTGGGATGACGGTACCCAAAGAATAAGCACCGGCTAACTTCGTGCCAGCAGCCGCGGTAATACGAAGGGTGCAAGCGTTACTCGGAATTACTGGGCGTAAAGCGTGCGTAGGTGGTCGTTTAAGTCCGTTGTGAAAGCCCTGGGCTCAACCTGGGAACTGCAGTGGATACTGGGCGACTAGAGTGTGGTAGAGGGTAGCGGAATTCCTGGTGTAGCAGTGAAATGCGTAGAGATCAGGAGGAACATCCATGGCGAAGGCAGCTACCTGGACCAACACTGACACTGAGGCACGAAAGCGTGGGGAGCAAACA
ASV766	Bacteria	Proteobacteria	Alphaproteobacteria	Sphingomonadales	Sphingomonadaceae	Sphingomonas	2	674	0	0	1	15	2	41	10	618		TGGGGAATATTGGACAATGGGCGAAAGCCTGATCCAGCAATGCCGCGTGAGTGATGAAGGCCTTAGGGTTGTAAAGCTCTTTTACCCGGGAAGATAATGACTGTACCGGGAGAATAAGCCCCGGCTAACTCCGTGCCAGCAGCCGCGGTAATACGGAGGGGGCTAGCGTTGTTCGGAATTACTGGGCGTAAAGCGCACGTAGGCGGCTTTGTAAGTCAGAGGTGAAAGCCTGGAGCTCAACTCCAGAACTGCCTTTGAGACTGCATCGCTTGAATCCAGGAGAGGTGAGTGGAATTCCGAGTGTAGAGGTGAAATTCGTAGATATTCGGAAGAACACCAGTGGCGAAGGCGGCTCACTGGACTGGTATTGACGCTGAGGTGCGAAAGCGTGGGGAGCAAACA
ASV825	Bacteria	Firmicutes	Bacilli	Bacillales	Bacillaceae	Bacillus	2	575	0	0	1	58	2	81	10	436		TAGGGAATCATCCGCAATGGACGAAAGTCTGACGGTGCAACGCCGCGTGAGTGATGAAGGATTTCGGTTCGTAAAGCTCTGTTGTTAGGGAAGAAACCGTGCCGAACGAACTAGGTCGGCACCTTGACGGTACCTAACCAGAAAGCCACGGCTAACTACGTGCCAGCAGCCGCGGTAATACGTAGGTGGCAAGCGTTGTCCGGAATTATTGGGCGTAAAGCGCGCGCAGGCGGTCTTTTAAGTCTGATGTGAAAGCCCCCGGCTCAACCGGGGAGGGTCATTGGAAACTGGGAGACTTGAGTACAGAAGAGGAGAGTGGAATTCCACGTGTAGCGGTGAAATGCGTAGATATGTGGAGGAACACCAGTGGCGAAGGCGACTCTCTGGTCTGTAACTGACGCTGAGGCGCGAAAGCGTGGGGAGCAAACA
ASV1031	Bacteria	Proteobacteria	Gammaproteobacteria	Xanthomonadales	Xanthomonadaceae	Stenotrophomonas	1	352	0	0	1	20	2	25	2	307		TGGGGAATATTGGACAATGGGCGCAAGCCTGATCCAGCCATACCGCGTGGGTGAAGAAGGCCTTCGGGTTGTAAAGCCCTTTTGTTGGGAAAGAAAAGCAGTCGGTTAATACCCGATTGTTCTGACGGTACCCAAAGAATAAGCACCGGCTAACTTCGTGCCAGCAGCCGCGGTAATACGAAGGGTGCAAGCGTTACTCGGAATTACTGGGCGTAAAGCGTGCGTAGGTGGTTGTTTAAGTCTGTCGTGAAAGCCCTGGGCTCAACCTGGGAATTGCGATGGAAACTGGGCGACTAGAGTGTGGCAGAGGGTAGTGGAATTCCTGGTGTAGCAGTGAAATGCGTAGAGATCAGGAGGAACATCCGTGGCGAAGGCGACTGCCTGGGCCAACACTGACACTGAGGCACGAAAGCGTGGGGAGCAAACA
ASV11	Bacteria	Proteobacteria	Gammaproteobacteria	Burkholderiales	Burkholderiaceae	Ralstonia	13	214042	21	2243	15	29623	1	53	20	182123		TGGGGAATTTTGGACAATGGGCGAAAGCCTGATCCAGCAATGCCGCGTGTGTGAAGAAGGCCTTCGGGTTGTAAAGCACTTTTGTCCGGAAAGAAATGGCTCTGGTTAATACCTGGGGTCGATGACGGTACCGGAAGAATAAGGACCGGCTAACTACGTGCCAGCAGCCGCGGTAATACGTAGGGTCCAAGCGTTAATCGGAATTACTGGGCGTAAAGCGTGCGCAGGCGGTTGTGCAAGACCGATGTGAAATCCCCGAGCTTAACTTGGGAATTGCATTGGTGACTGCACGGCTAGAGTGTGTCAGAGGGGGGTAGAATTCCACGTGTAGCAGTGAAATGCGTAGAGATGTGGAGGAATACCGATGGCGAAGGCAGCCCCCTGGGATAACACTGACGCTCATGCACGAAAGCGTGGGGAGCAAACA
ASV180	Bacteria	Proteobacteria	Gammaproteobacteria	Pseudomonadales	Pseudomonadaceae	Pseudomonas	3	7865	0	0	3	1088	1	218	20	6559		TGGGGAATATTGGACAATGGGCGAAAGCCTGATCCAGCCATGCCGCGTGTGTGAAGAAGGTCTTCGGATTGTAAAGCACTTTAAGTTGGGAGGAAGAGCAGTAAATTAATACTTTGCTGTTTTGACGTTACCGACAGAATAAGCACCGGCTAACTCTGTGCCAGCAGCCGCGGTAATACAGAGGGTGCAAGCGTTAATCGGAATTACTGGGCGTAAAGCGCGTAGGTGGTTCGTTAAGTTGGATGTGAAATCCCCGGGCTCAACCTGGGAACTGCATTCAAAACTGTCGAGCTAGAGTATGGTAGAGGGTGGTGGAATTTCCTGTGTAGCGGTGAAATGCGTAGATATAGGAAGGAACACCAGTGGCGAAGGCGACCACCTGGACTGATACTGACACTGAGGTGCGAAAGCGTGGGGAGCAAACA
ASV378	Bacteria	Proteobacteria	Gammaproteobacteria	Xanthomonadales	Xanthomonadaceae	Stenotrophomonas	2	2713	0	0	3	2308	1	50	5	355		TGGGGAATATTGGACAATGGGCGCAAGCCTGATCCAGCCATACCGCGTGGGTGAAGAAGGCCTTCGGGTTGTAAAGCCCTTTTGTTGGGAAAGAAATCCAGCTGGCTAATACCCGGTTGGGATGACGGTACCCAAAGAATAAGCACCGGCTAACTTCGTGCCAGCAGCCGCGGTAATACGAAGGGTGCAAGCGTTACTCGGAATTACTGGGCGTAAAGCGTGCGTAGGTGGTCGTTTAAGTCCGTTGTGAAAGCCCTGGGCTCAACCTGGGAACTGCAGTGGATACTGGACGACTAGAGTGTGGTAGAGGGTAGCGGAATTCCTGGTGTAGCAGTGAAATGCGTAGAGATCAGGAGGAACATCCATGGCGAAGGCAGCTACCTGGACCAACACTGACACTGAGGCACGAAAGCGTGGGGAGCAAACA
ASV950	Bacteria	Firmicutes	Bacilli	Staphylococcales	Staphylococcaceae	Staphylococcus	1	416	0	0	2	174	1	48	2	194		TAGGGAATCTTCCGCAATGGGCGAAAGCCTGACGGAGCAACGCCGCGTGAGTGATGAAGGGTTTCGGCTCGTAAAACTCTGTTATTAGGGAAGAACAAACGTGTAAGTAACTGTGCACGTCTTGACGGTACCTAATCAGAAAGCCACGGCTAACTACGTGCCAGCAGCCGCGGTAATACGTAGGTGGCAAGCGTTATCCGGAATTATTGGGCGTAAAGCGCGCGTAGGCGGTTTCTTAAGTCTGATGTGAAAGCCCACGGCTCAACCGTGGAGGGTCATTGGAAACTGGGAAACTTGAGTGCAGAAGAGGAAAGTGGAATTCCATGTGTAGCGGTGAAATGCGCAGAGATATGGAGGAACACCAGTGGCGAAGGCGACTTTCTGGTCTGTAACTGACGCTGATGTGCGAAAGCGTGGGGATCAAACA
ASV624	Bacteria	Proteobacteria	Alphaproteobacteria	Rhizobiales	Rhizobiaceae	Ochrobactrum	2	973	0	0	1	81	1	21	10	871		TGGGGAATATTGGACAATGGGCGCAAGCCTGATCCAGCCATGCCGCGTGAGTGATGAAGGCCCTAGGGTTGTAAAGCTCTTTCACCGGTGAAGATAATGACGGTAACCGGAGAAGAAGCCCCGGCTAACTTCGTGCCAGCAGCCGCGGTAATACGAAGGGGGCTAGCGTTGTTCGGATTTACTGGGCGTAAAGCGCACGTAGGCGGACTTTTAAGTCAGGGGTGAAATCCCAGAGCTCAACTCTGGAACTGCCTTTGATACTGGAAGTCTTGAGTATGGTAGAGGTGAGTGGAATTCCGAGTGTAGAGGTGAAATTCGTAGATATTCGGAGGAACACCAGTGGCGAAGGCGGCTCACTGGACCATTACTGACGCTGAGGTGCGAAAGCGTGGGGAGCAAACA
ASV1502	Bacteria	Bacteroidota	Bacteroidia	Flavobacteriales	Cryomorphaceae	NA	2	130	3	59	1	14	1	34	5	23		TGAGGAATATTGGTCAATGGAGGAAACTCTGAACCAGCCATGCCGCGTGAAGGATGACGGCCCTATGGGTTGTAAACTTCTTTTATATGGGAAGAAACTTATCTACGTGTAGATAACTGACGGTACCATACGAATAAGGATCGGCTAACTCCGTGCCAGCAGCCGCGGTAATACGGAGGATCCAAGCGTTATCCGGATTCATTGGGTTTAAAGGGTCCGTAGGCGGATTTTTAAGTCAGTGGTGAAAGCCGACAGCTCAACTGTCGAACTGCCATTGATACTGGAAATCTTGAGTACAAATGAAGTAGGCGGAATGAGTCATGTAGCGGTGAAATGCATAGATATGACTCAGAACACCGATTGCGAAGGCAGCTTACTAACATGTAACTGACGCTGAGGGACGAAAGCGTGGGGAGCGAACA
ASV491	Bacteria	Proteobacteria	Alphaproteobacteria	Rhizobiales	Rhizobiaceae	Pseudochrobactrum	1	1603	0	0	1	17	1	37	2	1549		TGGGGAATATTGGACAATGGGCGCAAGCCTGATCCAGCCATGCCGCGTGAGTGATGAAGGCCCTAGGGTTGTAAAGCTCTTTCACCGGTGAAGATAATGACGGTAACCGGAGAAGAAGCCCCGGCTAACTTCGTGCCAGCAGCCGCGGTAATACGAAGGGGGCTAGCGTTGTTCGGATTTACTGGGCGTAAAGCGCACGTAGGCGGACTTTTAAGTCAGGGGTGAAATCCCGGGGCTCAACCCCGGAACTGCCTTTGATACTGGAAGTCTTGAGTATGGAAGAGGTAAGTGGAATTGCGAGTGTAGAGGTGAAATTCGTAGATATTCGCAGGAACACCAGTGGCGAAGGCGGCTTACTGGTCCATTACTGACGCTGAGGTGCGAAAGCGTGGGGAGCAAACA
ASV734	Bacteria	Firmicutes	Bacilli	Bacillales	Bacillaceae	Anaerobacillus	1	712	0	0	1	3	1	8	10	701		TAGGGAATCTTCCGCAATGGACGAAAGTCTGACGGAGCAACGCCGCGTGAACGATGAAGGCCTTCGGGTCGTAAAGTTCTGTTGTTAGGGAAGAACAAGTACCGTTCAAATAGGGCGGTACCTTGACGGTACCTAACCAGAAAGCCACGGCTAACTACGTGCCAGCAGCCGCGGTAATACGTAGGTGGCAAGCGTTGTCCGGAATTATTGGGCGTAAAGCGCGCGCAGGCGGTCTCTTAAGTCTGATGTGAAATCTCGCGGCTCAACCGCGAGCGGCCATTGGAAACTGGGAGGCTTGAGTGCAGAAGAGGAGAGTGGAATTCCATGTGTAGCGGTGAAATGCGTAGATATATGGAGGAACACCAGTGGCGAAGGCGACTCTCTGGTCTGTAACTGACGCTGAGGCGCGAAAGCGTGGGGAGCAAACA
ASV793	Bacteria	Firmicutes	Clostridia	Clostridiales	Clostridiaceae	Clostridium_sensu_stricto_13	1	640	0	0	1	40	1	33	10	567		TGGGGAATATTGCGCAATGGGGGAAACCCTGACGCAGCAACGCCGCGTGAATGAAGAAGGCCTTAGGGTTGTAAAGTTCTGTTTTCTGGGACGATAATGACGGTACCAGAGGAGGAAGCCACGGCTAACTACGTGCCAGCAGCCGCGGTAATACGTAGGTGGCAAGCGTTGTCCGGATTTACTGGGCGTAAAGGATGCGTAGGCGGATGTTTAAGTGAGATGTGAAATACCCGAGCTCAACTTGGGTGCTGCATTTCAAACTGGATATCTAGAGTGCAGGAGAGGAAAGCGGAATTCCTAGTGTAGCGGTGAAATGCGTAGAGATTAGGAAGAACACCAGTGGCGAAGGCGGCTTTCTGGACTGTAACTGACGCTGAGGCATGAAAGCGTGGGGAGCAAACA
ASV877	Bacteria	Proteobacteria	Gammaproteobacteria	Burkholderiales	Oxalobacteraceae	Herbaspirillum	1	503	0	0	1	27	1	17	5	459		TGGGGAATTTTGGACAATGGGGGCAACCCTGATCCAGCAATGCCGCGTGAGTGAAGAAGGCCTTCGGGTTGTAAAGCTCTTTTGTCAGGGAAGAAACGGTAGTAGCTAATATCTATTACTAATGACGGTACCTGAAGAATAAGCACCGGCTAACTACGTGCCAGCAGCCGCGGTAATACGTAGGGTGCAAGCGTTAATCGGAATTACTGGGCGTAAAGCGTGCGCAGGCGGTTGTGTAAGTCAGATGTGAAATCCCCGGGCTCAACCTGGGAATTGCATTTGAGACTGCACGGCTAGAGTGTGTCAGAGGGGGGTAGAATTCCACGTGTAGCAGTGAAATGCGTAGATATGTGGAGGAATACCGATGGCGAAGGCAGCCCCCTGGGATAACACTGACGCTCATGCACGAAAGCGTGGGGAGCAAACA

Table S7. ASVs identified as potential contaminants

ASV no.		Phylum	Class	Order	Family	Genus	Total prevalence (%)	Total read count	Vaginal prevalence (%)	Vaginal read count	Cutaneous penile prevalence (%)	Cutaneous penile read count	Urine prevalence (%)	Urine read count	Negative control prevalence (%)	Negative control read count	Notes	Sequence
ASV1415	Bacteria	Proteobacteria	Gammaproteobacteria	Enterobacterales	Morganellaceae	Proteus	1	158	0	0	1	29	1	20	2	109		TGGGGAATATTGCACAATGGGCGCAAGCCTGATGCAGCCATGCCGCGTGTATGAAGAAGGCCTTAGGGTTGTAAAGTACTTTCAGCGGGGAGGAAGGTGATAAAGTTAATACCTTTATCAATTGACGTTACCCGCAGAAGAAGCACCGGCTAACTCCGTGCCAGCAGCCGCGGTAATACGGAGGGTGCAAGCGTTAATCGGAATTACTGGGCGTAAAGCGCACGCAGGCGGTCAATTAAGTCAGATGTGAAAGCCCCGAGCTTAACTTGGGAATTGCATCTGAAACTGGTTGGCTAGAGTCTTGTAGAGGGGGGTAGAATTCCACGTGTAGCGGTGAAATGCGTAGAGATGTGGAGGAATACCGGTGGCGAAGGCGGCCCCCTGGACAAAGACTGACGCTCAGGTGCGAAAGCGTGGGGAGCAAACA
ASV1398	Bacteria	Proteobacteria	Alphaproteobacteria	Caulobacterales	Caulobacteraceae	Brevundimonas	1	162	0	0	1	137	1	13	2	12		TGGGGAATCTTGCGCAATGGGCGAAAGCCTGACGCAGCCATGCCGCGTGAATGATGAAGGTCTTAGGATTGTAAAATTCTTTCACCGGGGACGATAATGACGGTACCCGGAGAAGAAGCCCCGGCTAACTTCGTGCCAGCAGCCGCGGTAATACGAAGGGGGCTAGCGTTGCTCGGAATTACTGGGCGTAAAGGGCGCGTAGGCGGATCGTTAAGTCAGAGGTGAAATCCCAGGGCTCAACCCTGGAACTGCCTTTGATACTGGCGATCTTGAGTATGAGAGAGGTATGTGGAACTCCGAGTGTAGAGGTGAAATTCGTAGATATTCGGAAGAACACCAGTGGCGAAGGCGACATACTGGCTCATTACTGACGCTGAGGCGCGAAAGCGTGGGGAGCAAACA
ASV785	Bacteria	Actinobacteriota	Actinobacteria	Micrococcales	Micrococcaceae	Nesterenkonia	1	650	0	0	0	0	1	51	2	599		TGGGGAATATTGCACAATGGGCGCAAGCCTGATGCAGCGACGCCGCGTGCGGGATGACGGCCTTCGGGTTGTAAACCGCTTTCAGCAGGGAAGAAGCTTTTGTGACGGTACCTGCAGAAGAAGCGCCGGCTAACTACGTGCCAGCAGCCGCGGTAATACGTAGGGCGCGAGCGTTATCCGGAATTATTGGGCGTAAAGAGCTCGTAGGCGGTTTGCCGCGTCTGCTGTGAAAGCCCGGGGCTTAACTCCGGGTGTGCAGTGGGTACGGGCAGACTAGAGTGCAGTAGGGGAGACTGGAATTCCTGGTGTAGCGGTGAAATGCGCAGATATCAGGAGGAACACCGATGGCGAAGGCAGGTCTCTGGGCTGTTACTGACGCTGAGGAGCGAAAGCATGGGGAGCGAACA
ASV679	Bacteria	Actinobacteriota	Actinobacteria	Corynebacteriales	Dietziaceae	Dietzia	2	839	0	0	5	296	1	56	2	487		TGGGGAATATTGCACAATGGGCGAAAGCCTGATGCAGCGACGCCGCGTGGGGGATGACGGTCTTCGGATTGTAAACCCCTTTCAGTAGGGACGAAGCGCAAGTGACGGTACCTGCAGAAGAAGCACCGGCCAACTACGTGCCAGCAGCCGCGGTAATACGTAGGGTGCGAGCGTTGTCCGGAATTACTGGGCGTAAAGAGCTCGTAGGCGGTTTGTCGCGTCGTCCGTGAAAACTCGGAGCTTAACTCCGAGCTTGCGGGCGATACGGGCAGACTTGAGTACTACAGGGGAGACTGGAATTCCTGGTGTAGCGGTGAAATGCGCAGATATCAGGAGGAACACCGGTGGCGAAGGCGGGTCTCTGGGTAGTAACTGACGCTGAGGAGCGAAAGCATGGGTAGCGAACA
ASV529	Bacteria	Actinobacteriota	Actinobacteria	Micrococcales	Micrococcaceae	Micrococcus	2	1400	0	0	5	767	1	204	2	429		TGGGGAATATTGCACAATGGGCGGAAGCCTGATGCAGCGACGCCGCGTGAGGGATGACGGCCTTCGGGTTGTAAACCTCTTTCAGTAGGGAAGAAGCGAAAGTGACGGTACCTGCAGAAGAAGCACCGGCTAACTACGTGCCAGCAGCCGCGGTAATACGTAGGGTGCGAGCGTTATCCGGAATTATTGGGCGTAAAGAGCTCGTAGGCGGTTTGTCGCGTCTGTCGTGAAAGTCCGGGGCTTAACCCCGGATCTGCGGTGGGTACGGGCAGACTAGAGTGCAGTAGGGGAGACTGGAATTCCTGGTGTAGCGGTGGAATGCGCAGATATCAGGAGGAACACCGATGGCGAAGGCAGGTCTCTGGGCTGTAACTGACGCTGAGGAGCGAAAGCATGGGGAGCGAACA
ASV895	Bacteria	Proteobacteria	Gammaproteobacteria	Pseudomonadales	Moraxellaceae	Acinetobacter	2	484	0	0	3	133	1	56	7	295		TGGGGAATATTGGACAATGGGGGGAACCCTGATCCAGCCATGCCGCGTGTGTGAAGAAGGCCTTATGGTTGTAAAGCACTTTAAGCGAGGAGGAGGCTACTGAGACTAATACTCTTGGATAGTGGACGTTACTCGCAGAATAAGCACCGGCTAACTCTGTGCCAGCAGCCGCGGTAATACAGAGGGTGCGAGCGTTAATCGGATTTACTGGGCGTAAAGCGTGCGTAGGCGGCTTTTTAAGTCGGATGTGAAATCCCCGAGCTTAACTTGGGAATTGCATTCGATACTGGGAAGCTAGAGTATGGGAGAGGATGGTAGAATTCCAGGTGTAGCGGTGAAATGCGTAGAGATCTGGAGGAATACCGATGGCGAAGGCAGCCATCTGGCCTAATACTGACGCTGAGGTACGAAAGCATGGGGAGCAAACA
ASV466	Bacteria	Proteobacteria	Gammaproteobacteria	Aeromonadales	Aeromonadaceae	Aeromonas	1	1826	0	0	1	139	1	7	7	1680		TGGGGAATATTGCACAATGGGGGAAACCCTGATGCAGCCATGCCGCGTGTGTGAAGAAGGCCTTCGGGTTGTAAAGCACTTTCAGCGAGGAGGAAAGGTCAGTAGCTAATACCTGCTGGCTGTGACGTTACTCGCAGAAGAAGCACCGGCTAACTCCGTGCCAGCAGCCGCGGTAATACGGAGGGTGCAAGCGTTAATCGGAATTACTGGGCGTAAAGCGCACGCAGGCGGTTGGATAAGTTAGATGTGAAAGCCCCGGGCTCAACCTGGGAATTGCATTTAAAACTGTCCAGCTAGAGTCTTGTAGAGGGGGGTAGAATTCCAGGTGTAGCGGTGAAATGCGTAGAGATCTGGAGGAATACCGGTGGCGAAGGCGGCCCCCTGGACAAAGACTGACGCTCAGGTGCGAAAGCGTGGGGAGCAAACA
ASV835	Bacteria	Proteobacteria	Gammaproteobacteria	Enterobacterales	Enterobacteriaceae	Citrobacter	1	567	0	0	1	22	1	27	7	518		TGGGGAATATTGCACAATGGGCGCAAGCCTGATGCAGCCATGCCGCGTGTATGAAGAAGGCCTTCGGGTTGTAAAGTACTTTCAGCGAGGAGGAAGGCGTTGTGGTTAATAACCGCAACGATTGACGTTACTCGCAGAAGAAGCACCGGCTAACTCCGTGCCAGCAGCCGCGGTAATACGGAGGGTGCAAGCGTTAATCGGAATTACTGGGCGTAAAGCGCACGCAGGCGGTCTGTCAAGTCGGATGTGAAATCCCCGGGCTCAACCTGGGAACTGCATCCGAAACTGGCAGGCTAGAGTCTTGTAGAGGGGGGTAGAATTCCAGGTGTAGCGGTGAAATGCGTAGAGATCTGGAGGAATACCGGTGGCGAAGGCGGCCCCCTGGACAAAGACTGACGCTCAGGTGCGAAAGCGTGGGGAGCAAACA
ASV1321	Bacteria	Proteobacteria	Gammaproteobacteria	Pseudomonadales	Pseudomonadaceae	Pseudomonas	1	171	0	0	1	139	1	16	2	16		TGGGGAATATTGGACAATGGGCGAAAGCCTGATCCAGCCATGCCGCGTGTGTGAAGAAGGTCTTCGGATTGTAAAGCACTTTAAGTTGGGAGGAAGGGCAGTAAGCTAATACCTTGCTGTTTTGACGTTACCGACAGAATAAGCACCGGCTAACTTCGTGCCAGCAGCCGCGGTAATACGAAGGGTGCAAGCGTTAATCGGAATTACTGGGCGTAAAGCGCGCGTAGGTGGTTCGTTAAGTTGGATGTGAAAGCCCCGGGCTCAACCTGGGAACTGCATCCAAAACTGGCGAGCTAGAGTACGGTAGAGGGTGGTGGAATTTCCTGTGTAGCGGTGAAATGCGTAGATATAGGAAGGAACACCAGTGGCGAAGGCGACCACCTGGACTGATACTGACACTGAGGTGCGAAAGCGTGGGGAGCAAACA
ASV413	Bacteria	Proteobacteria	Gammaproteobacteria	Pseudomonadales	Pseudomonadaceae	Pseudomonas	2	2337	0	0	0	0	1	6	22	2331		TGGGGAATATTGGACAATGGGCGAAAGCCTGACCCAGCCATGCCGCGTGTGTGAAGAAGGTCTTCGGATTGTAAAGCACTTTAAGTTGGGAGGAAGGGCAGTAAATTAATACTTTGCTGTTTTGACGTTACCGACAGAATAAGCACCGGCTAACTCTGTGCCAGCAGCCGCGGTAATACAGAGGGTGCAAGCGTTAATCGGAATTACTGGGCGTAAAGCGCGCGTAGGTGGTTCGTTAAGTTGGATGTGAAATCCCCGGGCTCAACCTGGGAACTGCATTCAAAACTGACGAGCTAGAGTATGGTAGAGGGTGGTGGAATTTCCTGTGTAGCGGTGAAATGCGTAGATATAGGAAGGAACACCAGTGGCGAAGGCGACCACCTGGACTGATACTGACACTGAGGTGCGAAAGCGTGGGGAGCAAACA
ASV912	Bacteria	Bacteroidota	Bacteroidia	Flavobacteriales	Weeksellaceae	Cloacibacterium	0	458	0	0	0	0	1	58	2	400		TGAGGAATATTGGTCAATGGGTGCAAGCCTGAACCAGCCATCCCGCGTGAAGGACGACTGCCCTATGGGTTGTAAACTTCTTTTGTATAGGGATAAACCTACCCTCGTGAGGGTAGCTGAAGGTACTATACGAATAAGCACCGGCTAACTCCGTGCCAGCAGCCGCGGTAATACGGAGGGTGCAAGCGTTATCCGGATTTATTGGGTTTAAAGGGTCCGTAGGCGGACTTATAAGTCAGTGGTGAAATCCTGTCGCTTAACGATAGAACTGCCATTGATACTGTAAGTCTTGAGTATATTTGAGGTAGCTGGAATAAGTAGTGTAGCGGTGAAATGCATAGATATTACTTAGAACACCAATTGCGAAGGCAGGTTACCAAGATATAACTGACGCTGAGGGACGAAAGCGTGGGGAGCGAACA
ASV973	Bacteria	Proteobacteria	Alphaproteobacteria	Rhizobiales	Rhizobiaceae	Pseudochrobactrum	0	394	0	0	0	0	1	32	2	362		TGGGGAATATTGGACAATGGGCGCAAGCCTGATCCAGCCATGCCGCGTGAGTGATGAAGGCCCTAGGGTTGTAAATCTCTTTCACCGGTGAAGATAATGACGGTAACCGGAGAAGAAGCCCCGGCTAACTTCGTGCCAGCAGCCGCGGTAATACGAAGGGGGCTAGCGTTGTTCGGATTTACTGGGCGTAAAGCGCACGTAGGCGGACTTTTAAGTCAGGGGTGAAATCCCGGGGCTCAACCCCGGAACTGCCTTTGATACTGGAAGTCTTGAGTATGGAAGAGGTAAGTGGAATTGCGAGTGTAGAGGTGAAATTCGTAGATATTCGCAGGAACACCAGTGGCGAAGGCGGCTTACTGGTCCATTACTGACGCTGAGGTGCGAAAGCGTGGGGAGCAAACA
ASV992	Bacteria	Firmicutes	Bacilli	Bacillales	Bacillaceae	Anaerobacillus	0	376	0	0	0	0	1	23	2	353		TAGGGAATCTTCCGCAATGGACGAAAGTCTGACGGAGCAACGCCGCGTGAACGATGAAGGCCTTCGGGTCGTAAAGTTCTGTTGTTAGGGAAGAACAAGTACCGTTCAAATAGGGCGGTACCTTGACGGTACCTAACCAGAAAGCCACGGCTAACTACGTGCCAGCAGCCGCGGTAATACGTAGGTGGCAAGCGTTGTCCGGAATTATTGGGCGTAAAGCGCGCGCAGGCGGTCTCTTAAGTCTGATGTGAAATCTCGCGGCTCAACCGCGAGCGGTCATTGGAAACTGGGAGACTTGAGTGCAGAAGAGGAGAGTGGAATTCCATGTGTAGCGGTGAAATGCGTAGATATATGGAGGAACACCAGTGGCGAAGGCGACTCTCTGGTCTGTAACTGACGCTGAGGCGCGAAAGCGTGGGGAGCAAACA
ASV1004	Bacteria	Proteobacteria	Alphaproteobacteria	Rhizobiales	Rhizobiaceae	Phyllobacterium	2	366	0	0	3	310	0	0	7	56		TGGGGAATATTGGACAATGGGCGCAAGCCTGATCCAGCCATGCCGCGTGAGTGATGAAGGCCCTAGGGTTGTAAAGCTCTTTCACCGGTGAAGATAATGACGGTAACCGGAGAAGAAGCCCCGGCTAACTTCGTGCCAGCAGCCGCGGTAATACGAAGGGGGCTAGCGTTGTTCGGATTTACTGGGCGTAAAGCGCACGTAGGCGGACTATTAAGTCAGGGGTGAAATCCCGGGGCTCAACCCCGGAACTGCCTTTGATACTGGTAGTCTTGAGTTCGAGAGAGGTGAGTGGAATTCCGAGTGTAGAGGTGAAATTCGTAGATATTCGGAGGAACACCAGTGGCGAAGGCGGCTCACTGGCTCGATACTGACGCTGAGGTGCGAAAGCGTGGGGAGCAAACA
ASV1168	Bacteria	Proteobacteria	Gammaproteobacteria	Pseudomonadales	Pseudomonadaceae	Pseudomonas	1	260	0	0	2	152	0	0	2	108		TGGGGAATATTGGACAATGGGCGAAAGCCTGATCCAGCCATGCCGCGTGTGTGAAGAAGGTCTTCGGATTGTAAAGCACTTTAAGTTGGGAGGAAGGGCATTAACCTAATACGTTAGTGTTTTGACGTTACCGACAGAATAAGCACCGGCTAACTTCGTGCCAGCAGCCGCGGTAATACGAAGGGTGCAAGCGTTAATCGGAATTACTGGGCGTAAAGCGCGCGTAGGTGGTTCGTTAAGTTGGATGTGAAAGCCCCGGGCTCAACCTGGGAACTGCATCCAAAACTGGCGAGCTAGAGTACGGTAGAGGGTGGTGGAATTTCCTGTGTAGCGGTGAAATGCGTAGATATAGGAAGGAACACCAGTGGCGAAGGCGACCACCTGGACTGATACTGACACTGAGGTGCGAAAGCGTGGGGAGCAAACA

Table S7. ASVs identified as potential contaminants

ASV no.		Phylum	Class	Order	Family	Genus	Total prevalence (%)	Total read count	Vaginal prevalence (%)	Vaginal read count	Cutaneous penile prevalence (%)	Cutaneous penile read count	Urine prevalence (%)	Urine read count	Negative control prevalence (%)	Negative control read count	Notes	Sequence
ASV1181	Bacteria	Proteobacteria	Gammaproteobacteria	Burkholderiales	Alcaligenaceae	Achromobacter	1	255	0	0	2	244	0	0	2	11		TGGGGAATTTTGGACAATGGGGGAAACCCTGATCCAGCCATCCCGCGTGTGCGATGAAGGCCTTCGGGTTGTAAAGCACTTTTGGCAGGAAAGAAACGTCATGGGTTAATACCCCGTGAAACTGACGGTACCTGCAGAATAAGCACCGGCTAACTACGTGCCAGCAGCCGCGGTAATACGTAGGGTGCAAGCGTTAATCGGAATTACTGGGCGTAAAGCGTGCGCAGGCGGTTCGGAAAGAAAGATGTGAAATCCCAGAGCTTAACTTTGGAACTGCATTTTTAACTACCGGGCTAGAGTGTGTCAGAGGGAGGTGGAATTCCGCGTGTAGCAGTGAAATGCGTAGATATGCGGAGGAACACCGATGGCGAAGGCAGCCTCCTGGGATAACACTGACGCTCATGCACGAAAGCGTGGGGAGCAAACA
ASV720	Bacteria	Proteobacteria	Gammaproteobacteria	Burkholderiales	Comamonadaceae	Pelomonas	1	736	0	0	1	52	0	0	12	684		TGGGGAATTTTGGACAATGGGCGCAAGCCTGATCCAGCCATGCCGCGTGCGGGAAGAAGGCCTTCGGGTTGTAAACCGCTTTTGTCAGGGAAGAAAAGGTTCTGGTTAATACCTGGGACTCATGACGGTACCTGAAGAATAAGCACCGGCTAACTACGTGCCAGCAGCCGCGGTAATACGTAGGGTGCAAGCGTTAATCGGAATTACTGGGCGTAAAGCGTGCGCAGGCGGTTATGCAAGACAGAGGTGAAATCCCCGGGCTCAACCTGGGAACTGCCTTTGTGACTGCATAGCTAGAGTACGGTAGAGGGGGATGGAATTCCGCGTGTAGCAGTGAAATGCGTAGATATGCGGAGGAACACCGATGGCGAAGGCAATCCCCTGGACCTGTACTGACGCTCATGCACGAAAGCGTGGGGAGCAAACA
ASV1176	Bacteria	Proteobacteria	Gammaproteobacteria	Burkholderiales	Oxalobacteraceae	Herbaspirillum	1	133	0	0	1	73	0	0	5	60		TGGGGAATTTTGGACAATGGGGGCAACCCTGATCCAGCAATGCCGCGTGAGTGAAGAAGGCCTTCGGGTTGTAAAGCTCTTTTGTCAGGGAAGAAACGGTAGTAGCGAATAACTATTACTAATGACGGTACCTGAAGAATAAGCACCGGCTAACTACGTGCCAGCAGCCGCGGTAATACGTAGGGTGCAAGCGTTAATCGGAATTACTGGGCGTAAAGCGTGCGCAGGCGGTTGTGTAAGTCAGATGTGAAATCCCCGGGCTCAACCTGGGAATTGCATTTGAGACTGCACGGCTAGAGTGTGTCAGAGGGGGGTAGAATTCCACGTGTAGCAGTGAAATGCGTAGATATGTGGAGGAATACCGATGGCGAAGGCAGCCCCCTGGGATAACACTGACGCTCATGCACGAAAGCGTGGGGAGCAAACA
ASV507	Bacteria	Proteobacteria	Gammaproteobacteria	Burkholderiales	Alcaligenaceae	Achromobacter	1	1470	0	0	1	3	0	0	10	1467		TGGGGAATTTTGGACAATGGGGGAAACCCTGATCCAGCCATCCCGCGTGTGCGATGAAGGCCTTCGGGTTGTAAAGCACTTTTGGCAGGAAAGAAACGTCATGGGCTAATACCCCGTGAAACTGACGGTACCTGCAGAATAAGCACCGGCTAACTACGTGCCAGCAGCCGCGGTAATACGTAGGGTGCAAGCGTTAATCGGAATTACTGGGCGTAAAGCGTGCGCAGGCGGTTCGGAAAGAAAGATGTGAAATCCCAGAGCTTAACTTTGGAACTGCATTTTTAACTACCGAGCTAGAGTGTGTCAGAGGGAGGTGGAATTCCGCGTGTAGCAGTGAAATGCGTAGATATGCGGAGGAACACCGATGGCGAAGGCAGCCTCCTGGGATAACACTGACGCTCATGCACGAAAGCGTGGGGAGCAAACA
ASV494	Bacteria	Proteobacteria	Gammaproteobacteria	Burkholderiales	Oxalobacteraceae	Duganella	1	1589	0	0	1	141	0	0	7	1448		TGGGGAATTTTGGACAATGGGGGCAACCCTGATCCAGCAATGCCGCGTGAGTGAAGAAGGCCTTCGGGTTGTAAAGCTCTTTTGTCAGGGAAGAAACGGTGAGGGCTAATATCTCTTGCTAATGACGGTACCTGAAGAATAAGCACCGGCTAACTACGTGCCAGCAGCCGCGGTAATACGTAGGGTGCAAGCGTTAATCGGAATTACTGGGCGTAAAGCGTGCGCAGGCGGTTTTGTAAGACTGTCGTGAAATCCCCGGGCTCAACCTGGGAATGGCGATGGTGACTGCAAGGCTAGAGTTTGGCAGAGGGGGGTAGAATTCCACGTGTAGCAGTGAAATGCGTAGATATGTGGAGGAACACCGATGGCGAAGGCAGCCCCCTGGGTCAAAACTGACGCTCATGCACGAAAGCGTGGGGAGCAAACA
ASV810	Bacteria	Patescibacteria	Gracilibacteria	Candidatus_Peribacteria	NA	NA	0	608	0	0	1	83	0	0	2	525		TTAGGAATCTTCCACAATGGGCGCAAGCCTGATGGAGCGACACCGCGTGAAGGATGAAGCCCTTTCTGGGTGTAAACTTCTGTTCTGAGGGACGAAATTTTTGACGGTACCTCAGGAGAAAGCACCGGCTAATTCTGTGCCAGCAGCCGCGGTAAGACAGAAGGTGCAAGCGTTACTCGGAATTACTGGGCGTAAAGCGTCTGTAGGTGTCTTTCCACGTCTGGCAGTAAATCACGGGGCTCAACCCCGTATCTCTGCCGGAAACGAGAAAGATCGAGTCATTCAGAGGCATCTGGAATGTCGTGTGTAGGGGTAAAATCCGTAGATACACGATGGAACGCCAAAAGCGAAGGCAGGATGCTAGGAATGTACTGACACTCAGAGACGAAAGCGTGGGGAGCAAAGG
ASV1034	Bacteria	Firmicutes	Bacilli	Bacillales	Bacillaceae	Bacillus	1	336	0	0	1	19	0	0	7	317		TAGGGAATCTTCCACAATGGACGAAAGTCTGATGGAGCAACGCCGCGTGAGCGATGAAGGCCTTCGGGTCGTAAAGCTCTGTTGTTAGGGAAGAACAAGTGCGAGAGTAACTGCTCGCGCCTTGACGGTACCTAACCAGAAAGCCACGGCTAACTACGTGCCAGCAGCCGCGGTAATACGTAGGTGGCAAGCGTTGTCCGGAATTATTGGGCGTAAAGCGCGCGCAGGCGGTTTCTTAAGTCTGATGTGAAAGCCCACGGCTCAACCGTGGAGGGTCATTGGAAACTGGGAGACTTGAGTGCAGAAGAGGAGAGTGGAATTCCACGTGTAGCGGTGAAATGCGTAGAGATGTGGAGGAACACCAGTGGCGAAGGCGACTCTCTGGTCTGTAACTGACGCTGAGGCGCGAAAGCGTGGGGAGCAAACA
ASV1094	Bacteria	Proteobacteria	Gammaproteobacteria	Burkholderiales	Burkholderiaceae	Ralstonia	0	300	0	0	1	31	0	0	2	269		TGGGGAATTTTGGACAATGGGGGCAACCCTGATCCAGCAATGCCGCGTGTGTGAAGAAGGCCTTCGGGTTGTAAAGCACTTTTGTCCGGAAAGAAATCGCACTTACTAATATTAGGTGTGGATGACGGTACCGGAAGAATAAGGACCGGCTAACTACGTGCCAGCAGCCGCGGTAATACGTAGGGTCCAAGCGTTAATCGGAATTACTGGGCGTAAAGCGTGCGCAGGCGGTTGTGCAAGACCGATGTGAAATCCCCGGGCTTAACCTGGGAATTGCATTGGTGACTGCACGGCTAGAGTGTGTCAGAGGGGGGTAGAATTCCACGTGTAGCAGTGAAATGCGTAGAGATGTGGAGGAATACCGATGGCGAAGGCAGCCCCCTGGGATAACACTGACGCTCATGCACGAAAGCGTGGGGAGCAAACA
ASV1276	Bacteria	Proteobacteria	Gammaproteobacteria	Burkholderiales	Comamonadaceae	Pelomonas	0	204	0	0	1	6	0	0	2	198		TGGGGAATTTTGGACAATGGGCGCAAGCCTGATCCAGCCATGCCGCGTGCGGGAAGAAGGCCTTCGGGTTGTAAACCGCTTTTGTCAGGGAAGAAAAGGTTCTGGCTAATACCTGGGACTCATGACGGTACCTGAAGAATAAGCACCGGCTAACTACGTGCCAGCAGCCGCGGTAATACGTAGGGTGCAAGCGTTAATCGGAATTACTGGGCGTAAAGCGTGCGCAGGCGGTTATGCAAGACAGAGGTGAAATCCCCGGGCTCAACCTGGGAACTGCCTTTGTGACTGCATGGCTAGAGTACGGTAGAGGGGGATGGAATTCCGCGTGTAGCAGTGAAATGCGTAGATATGCGGAGGAACACCGATGGCGAAGGCAATCCCCTGGACCTGTACTGACGCTCATGCACGAAAGCGTGGGGAGCAAACA
ASV1156	Bacteria	Proteobacteria	Gammaproteobacteria	Burkholderiales	Oxalobacteraceae	Herbaspirillum	0	235	0	0	1	66	0	0	2	169		TGGGGAATTTTGGACAATGGGGGCAACCCTGATCCAGCAATGCCGCGTGAGTGAAGAAGGCCTTCGGGTTGTAAAGCTCTTTTGTCAGGGAAGAAACGGTAGTAGCGAATAACTACTACTAATGACGGTACCTGAAGAATAAGCACCGGCTAACTACGTGCCAGCAGCCGCGGTAATACGTAGGGTGCAAGCGTTAATCGGAATTACTGGGCGTAAAGCGTGCGCAGGCGGTTGTGTAAGTCAGATGTGAAATCCCCGGGCTCAACCTGGGAATTGCATTTGAGACTGCACGGCTAGAGTGTGTCAGAGGGGGGTAGAATTCCACGTGTAGCAGTGAAATGCGTAGATATGTGGAGGAATACCGATGGCGAAGGCAGCCCCCTGGGATAACACTGACGCTCATGCACGAAAGCGTGGGGAGCAAACA
ASV407	Bacteria	Proteobacteria	Gammaproteobacteria	Legionellales	Legionellaceae	Legionella	0	2399	0	0	0	0	0	0	5	2399		TGGGGAATATTGGACAATGGGGGCAACCCTGATCCAGCAATGCCGCGTGTGTGAAGAAGGCCTGAGGGTTGTAAAGCACTTTCATTGGGGAGGAGGATTGATTGGTTAAGAGCTGATTGATTGGACGTTACCCAGATAAGAAGCACCGGCTAACTCCGTGCCAGCAGCCGCGGTAATACGGAGGGTGCAAGCGTTAATCGGAATTACTGGGCGTAAAGGGTTCGTAGGTGGTTTGTTAAGTTAACTGTGAAATCCCTGGGCTTAACCTGGGGCGGTCAGTTAAGACTGATAGACTTGAGTATGGGAGAGGGTAGTGGAATTTCCGGTGTAGCGGTGAAATGCGTAGAGATCGGAAGGAACACCAGTGGCGAAGGCGGCTACCTGGCCTGATACTGACACTGAGGAACGAAAGCGTGGGGAGCAAACA
ASV564	Bacteria	Firmicutes	Bacilli	Bacillales	Bacillaceae	Bacillus	0	1218	0	0	0	0	0	0	2	1218		TAGGGAATCATCCGCAATGGACGAAAGTCTGACGGTGCAACGCCGCGTGAGTGATGAAGGATTTCGGTTCGTAAAGCTCTGTTGTTAGGGAAGAACAAGTGCCGTTCGAATAGGTCGGCACCTTGACGGTACCTAACCAGAAAGCCACGGCTAACTACGTGCCAGCAGCCGCGGTAATACGTAGGTGGCAAGCGTTGTCCGGAATTATTGGGCGTAAAGCGCGCGCAGGCGGTCTTTTAAATCTGATGTGAAAGCCCCCGGCTCAACCGGGGAGGGTCATTGGAAACTGGGAGACTTGAGTACAGAAGAGGAGAGTGGAATTCCACGTGTAGCGGTGAAATGCGTAGATATGTGGAGGAACACCAGTGGCGAAGGCGACTCTCTGGTCTGTAACTGACGCTGAGGCGCGAAAGCGTGGGGAGCAAACA
ASV585	Bacteria	Firmicutes	Bacilli	Bacillales	Bacillaceae	Bacillus	0	1142	0	0	0	0	0	0	2	1142		TAGGGAATCTTCCACAATGGACGAAAGTCTGATGGAGCAACGCCGCGTGAGCGATGAAGGCCTTCGGGTCGTAAAGCTCTGTTGTTAGGGAAGAACAAGTGCGAGAGTAACTGCTCGCACCTTGACGGTACCTAACCAGAAAGCCACGGCTAACTACGTGCCAGCAGCCGCGGTAATACGTAGGTGGCAAGCGTTGTCCGGAATTATTGGGCGTAAAGCGCGCGCAGGCGGTTTCTTAAGTCTGATGTGAAAGCCCACGGCTCAACCGTGGAGGGTCATTGGAAACTGGGAGACTTGAGTGCAGAAGAGGAGAGTGGAATTCCACGTGTAGAGGTGAAATGCGTAGAGATGTGGAGGAACACCAGTGGCGAAGGCGACTCTCTGGTCTGTAACTGACGCTGAGGCGCGAAAGCGTGGGGAGCAAACA
ASV598	Bacteria	Proteobacteria	Gammaproteobacteria	Pseudomonadales	Pseudomonadaceae	Pseudomonas	0	1073	0	0	0	0	0	0	2	1073		TGGGGAATATTGGACAATGGGCGAAAGCCTGATCCAGCCATGCCGCGTGTGTGAAGAAGGTCTTCGGATTGTAAAGCACTTTAAGTTGGGAGGAAGGGCAGTAAATTAATACTTTGCTGTTTTGACGTTACCGACAGAATAAGCACCGGCTAACTCTGTGCCAGCAGCCGCGGTAATACAGAGGGTGCAAGCGTTAATCGGAATTACTGGGCGTAAAGCGCGCGTAGGTGGTTCGTTAAGTTGGATGTGAAATCCCCGGACTCAACCTGGGAACTGCATTCAAAACTGACGAGCTAGAGTATGGTAGAGGGTGGTGGAATTTCCTGTGTAGCGGTGAAATGCGTAGATATAGGAAGGAACACCAGTGGCGAAGGCGACCACCTGGACTGATACTGACACTGAGGTGCGAAAGCGTGGGGAGCAAACA
ASV737	Bacteria	Proteobacteria	Gammaproteobacteria	Burkholderiales	Oxalobacteraceae	Duganella	0	708	0	0	0	0	0	0	5	708		TGGGGAATTTTGGACAATGGGGGCAACCCTGATCCAGCAATGCCGCGTGAGTGAAGAAGGCCTTCGGGTTGTAAAGCTCTTTTGTCAGGGAAGAAACGGTGAGGGCTAATATCCCTTGCTAATGACGGTACCTGAAGAATAAGCACCGGCTAACTACGTGCCAGCAGCCGCGGTAATACGTAGGGTGCAAGCGTTAATCGGAATTACTGGGCGTAAAGCGTGCGCAGGCGGTTTTGTAAGACTGTCGTGAAATCCCCGGGCTCAACCTGGGAATGGCGATGGTGACTGCAAGGCTAGAGTTTGGCAGAGGGGGGTAGAATTCCACGTGTAGCAGTGAAATGCGTAGATATGTGGAGGAACACCGATGGCGAAGGCAGCCCCCTGGGTCAAAACTGACGCTCATGCACGAAAGCGTGGGGAGCAAACA

Table S7. ASVs identified as potential contaminants

ASV no.		Phylum	Class	Order	Family	Genus	Total prevalence (%)	Total read count	Vaginal prevalence (%)	Vaginal read count	Cutaneous penile prevalence (%)	Cutaneous penile read count	Urine prevalence (%)	Urine read count	Negative control prevalence (%)	Negative control read count	Notes	Sequence
ASV1201	Bacteria	Bacteroidota	Bacteroidia	Bacteroidales	Muribaculaceae	NA	0	247	0	0	0	0	0	0	5	247		TGAGGAATATTGGTCAATGGGCGCGAGCCTGAACCAGCCAAGTCGCGTGAGGGAGGACGGCCCTACGGGTTGTAAACCTCTTTTGCCGGGGAGCAACGGGCGTCACGTGTGGCGCCACTGAGAGTACCCGGAGAAAAAGCATCGGCTAACTCCGTGCCAGCAGCCGCGGTAATACGGAGGATGCGAGCGTTATCCGGATTTATTGGGTTTAAAGGGTGCGTAGGCGGATCGTTAAGTCAGTGGTCAAATTGAGGGGCTCAACCCCTTCCCGCCATTGAAACTGGCGTTCTTGAGTGGAAGAGAAGTATGCGGAATGCGTGGTGTAGCGGTGAAATGCATAGATATCACGCAGAACCCCGATTGCGAAGGCAGCATGCCGGCTTCCGACTGACGCTGAAGCACGAAAGCGTGGGGATCGAACA
ASV1268	Bacteria	Firmicutes	Bacilli	Bacillales	Bacillaceae	Anaerobacillus	0	207	0	0	0	0	0	0	5	207		TAGGGAATCTTCCGCAATGGACGAAAGTCTGACGGAGCAACGCCGCGTGAACGATGAAGGCCTTCGGGTCGTAAAGTTCTGTTGTTAGGGAAGAACAAGTACCGTTCAAATAGGTCGGTACCTTGACGGTACCTAACCAGAAAGCCACGGCTAACTACGTGCCAGCAGCCGCGGTAATACGTAGGTGGCAAGCGTTGTCCGGAATTATTGGGCGTAAAGCGCGCGCAGGCGGTCTCTTAAGTCTGATGTGAAAGCCCACGGCTCAACCGTGGAGGGCCATTGGAAACTGGGAGACTTGAGTGCAGAAGAGGAGAGTGGAATTCCATGTGTAGCGGTGAAATGCGTAGATATATGGAGGAACACCAGTGGCGAAGGCGACTCTCTGGTCTGTAACTGACGCTGAGGCGCGAAAGCGTGGGGAGCAAACA
